# Allosteric Regulation of Glycogen Phosphorylase by Order/Disorder Transition of the 250′ and 280s Loops

**DOI:** 10.1021/acs.biochem.2c00671

**Published:** 2023-03-29

**Authors:** Monika Kish, Sivaraman Subramanian, Victoria Smith, Natasha Lethbridge, Lindsay Cole, Frank Vollmer, Nicholas. J. Bond, Jonathan J. Phillips

**Affiliations:** † Living Systems Institute, Department of Biosciences, University of Exeter, Stocker Road, Exeter, EX4 4QD, U.K.; ‡ Living Systems Institute, Department of Physics, University of Exeter, Stocker Road, Exeter, EX4 6QD, U.K.; § CPI, Darlington, DL1 1GL, U.K.; ∥ Applied Photophysics Ltd, Leatherhead, KT227BA, U.K.; ⊥ Analytical Sciences, Biopharmaceutical Development, BioPharmaceuticals R&D, AstraZeneca, Milstein Building, Granta Park, Cambridge, CB21 6GH, U.K.; # Alan Turing Institute, British Library, London, NW1 2DB, U.K.

## Abstract

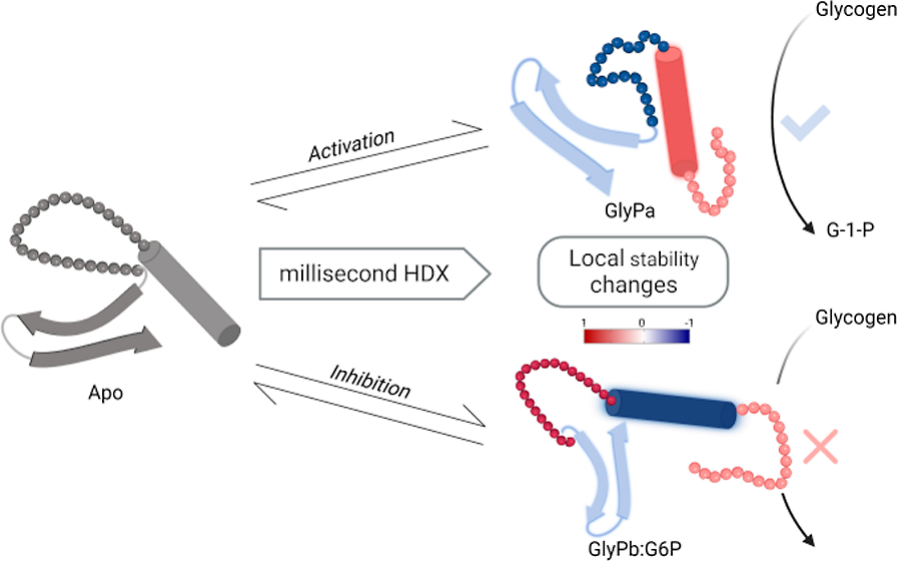

Allostery is a fundamental mechanism of protein activation, yet the precise dynamic changes that underlie functional regulation of allosteric enzymes, such as glycogen phosphorylase (GlyP), remain poorly understood. Despite being the first allosteric enzyme described, its structural regulation is still a challenging problem: the key regulatory loops of the GlyP active site (250′ and 280s) are weakly stable and often missing density or have large b-factors in structural models. This led to the longstanding hypothesis that GlyP regulation is achieved through gating of the active site by (dis)order transitions, as first proposed by Barford and Johnson. However, testing this requires a quantitative measurement of weakly stable local structure which, to date, has been technically challenging in such a large protein. Hydrogen–deuterium-exchange mass spectrometry (HDX-MS) is a powerful tool for studying protein dynamics, and millisecond HDX-MS has the ability to measure site-localized stability differences in weakly stable structures, making it particularly valuable for investigating allosteric regulation in GlyP. Here, we used millisecond HDX-MS to measure the local structural perturbations of glycogen phosphorylase b (GlyPb), the phosphorylated active form (GlyPa), and the inhibited glucose-6 phosphate complex (GlyPb:G6P) at near-amino acid resolution. Our results support the Barford and Johnson hypothesis for GlyP regulation by providing insight into the dynamic changes of the key regulatory loops.

Almost 60 years ago, the term “allostery” was coined by Monod and Jacob to describe the structural and functional regulation of proteins by “nonsteric” means.^1−4^ The archetypal allosteric enzyme is glycogen phosphorylase (GlyP), and much insight has been gained from high-resolution structures of trapped *R*/*T*-states of GlyP (*T*—tense GlyPb inactive state and *R*—relaxed GlyPa state), with seminal work by Barford and co-workers^5−7^ but also from low-resolution studies of enzyme kinetics.^8,9^ An open question regarding functional control of GlyP is reflected across much of enzymology: What are the quantitative changes in local stability (ΔΔ*G*) between *R*/*T*-states in solution that underpin allosteric conformer selection?^10−13^ Although GlyP was the first allosteric enzyme to be identified, this is a pivotal and timely question as it is an important and proven therapeutic target for patients with type II diabetes,^14,15^ cancers,^16^ and neurodegenerative diseases.^17^ Structural biology efforts have revealed a great deal of detail of the alternative postures adopted by GlyP: significantly, some crystallographic models indicated missing density in the 280s loop in the *R*-state (9GPB.pdb), which condenses to a defined conformation in the *T*-state (1GPB.pdb).^18−20^ This has been interpreted qualitatively as the critical structural feature to gate catalytic activity—sterically regulating substrate access to the active site. Recent HDX-MS work described the structural dynamics of the proteins;^21^ however, we provide direct stability changes of the key regulatory loops arising from activation/deactivation of GlyPb. Estimates of quantitative local stability measurements, as are presented here, are required to strengthen the longstanding hypothesis for GlyP gating of the active site^9^ and becoming mobile, as first proposed by Barford and Johnson.^5^

In smaller protein systems, it is tractable to measure the local structure and stability. If the structure is fully stable, then different approaches allow high structural resolution of the beginning and end states of transitioning proteins, for example, cryo-EM^22^ and X-ray crystallography.^23^ Several time-dependent approaches have been used, for example, X-ray absorption spectroscopy,^24^ nuclear magnetic resonance (NMR),^25^ and fluorescence spectroscopy.^26^ Hydrogen–deuterium-exchange mass spectrometry (HDX-MS) can also be applied to measure structural dynamics and, employing rapid mixing systems, can observe natively disordered regions, such as the 280s and 250′ loops in GlyP.^27−29^ To this end, we previously developed a fully automated “bottom-up” HDX-MS instrument that expands the time window by 4 orders of magnitude; thus, it is capable of an accurate quantitative assessment of peptide stability in solution, even with no apparent stability^30^ (Kish et al.’s manuscript in press)^100^. Measurement of the structural dynamics and/or local stability in highly dynamic regions within large proteins can be obtained through fully quantitative analysis. Furthermore, by an automated collection of additional time points, a high level of certainty can be attained in determining subtle differences in dynamics, even between similar exchange rates. This represents a significant advancement in the capabilities for protein biophysics research with the particular advantage of yielding information on disordered proteins/regions contributing to the mechanism of protein functional regulation.

In order to quantitatively evaluate local changes in stability within a protein from HDX-MS measurements, it is useful to calculate a protection factor (Pf) relative to a reference value. Absolute Pf, defined as *k*_int_/*k*_exp_, is frequently used as a measure of the reduced H/D exchange brought by the structure of the protein, where *k*_int_ is defined as the rate constant for exchange of the reference peptide state and *k*_exp_ is the rate observed experimentally. Therefore, an accurate estimate of *k*_int_ is ideally available, representing the fastest possible exchange, given no residual protein structure.^28,31^ While not accurate in all cases, the *k*_int_ values determined by Englander and co-workers have proven to be useful in the estimation of Pf^32^ and local stability (Δ*G*)^33^ and agree with values determined by NMR.

Here, we quantitatively describe local structural allostery from measurements of the GlyP dimer in solution. This provides a quantitative map of GlyP stability under allosteric activation and inhibition. Our goal was to quantify the structural switch of GlyP in solution between activated (pSer14) and inhibited (glucose-6-phosphate bound) forms. This shows the estimated changes in local stability in response to allosteric modulation, notably in the order/disorder transition of the 280s loop that gates access to the active site, as proposed from crystal structural models in 1989 by Barford and Johnson.

## Materials and Methods

### Chemicals and Reagents

GlyP b from rabbit muscle and GlyP a from rabbit muscle were purchased from Sigma. Chemicals were purchased as follows: glucose-6-phosphate, tris hydrochloride, tris(2-carboxyethyl) phosphine hydrochloride (TCEP), and dimethyl sulfoxide-d6 (99.96%) from Sigma. Deuterium oxide (99.9% D) was purchased from Goss Scientific. Water, acetonitrile, and formic acid (99.5%) Optima LC/MS Grade were obtained from Fisher Scientific. All other ultrapure water used was purified on a Milli-Q Advantage A10 system (Merck).

### Sample Preparation

The protein was dissolved and diluted in 40 mM Tris hydrochloride and 1 mM TCEP at pH 7.00 to a final concentration of 10 μM. For the GlyP b and inhibitor (glucose-6 phosphate) equilibrium experiments, the same buffers and concentrations were used, with a GlyP b-to-glucose-6-phosphate ratio of 1:200.

### GlyP Activity Assay

The activity of each of the GlyP forms was confirmed by a glycogen phosphorylase assay kit (Colorimetric) (Abcam, ab273271). The assay was performed according to the instructions of the kit without any modifications. In brief, samples were dissolved under identical conditions as for GlyPb, GlyPa, and GlyPb/G6P throughout HDX experiments, comprising 10 μM enzyme and 2 mM G6P. Directions from the assay were followed, and absorbance was measured at 450 nm in kinetic mode for 60 min at 30 °C for three replicates.

### Millisecond Hydrogen–Deuterium Exchange

Protein samples (20 μL) were labeled with the ms2min system, at nine time points (0.05, 0.15, 0.25, 0.35, 0.5, 1, 5, 30, and 300 s), randomly *n* = 3. All labeling experiments were performed at 23 °C, further quenched, and analyzed at 0 °C. A 72 in. long tubing was used to connect the ms2min system to the digestion/separation chamber of the Waters HDX Manager. Protein samples were digested online with a pepsin column (Waters). Buffers used were 40 mM tris hydrochloride, 1 mM TCEP, pH 7.00 in H_2_O, 40 mM Tris hydrochloride, and 1 mM TCEP at pD 7.00 in D_2_0 and 100 mM potassium phosphate, pH 2.50 in H_2_O, as equilibrium, labeling and quench buffers, respectively.

### Back-Exchange Correction

For back-exchange correction of the protein samples,^31^ GlyP b was predigested offline in a fully deuterated control buffer, 20 mM potassium phosphate buffer, at pH 2.55 in D_2_O with pepsin, for 5, 10, and 30 min at RT. The digested fully deuterated peptides were then manually injected, processed, and analyzed using the HDX-MS workflow described above, with the only exception that a narrow-bore union was placed instead of the pepsin column to avoid double digestion.

### Liquid Chromatography–Mass Spectrometry

Waters HDX Manager was used for digestion, desalting, and separation of the peptides with an immobilized pepsin column (Enzymate BEH Pepsin Column 2.1 × 30 mm, 5 μm); C18 trapping column (VanGuard ACQUITY BEH 1.7 μm, 2.1 × 5 mm; Waters), and analytical C18 column (1.7 μm, 1.0 × 100 mm ACQUITY BEH; Waters), correspondingly. Standard mobile phases were used during liquid chromatography, 0.1% formic acid in H_2_O pH 2.50 (A) and 0.1% formic acid in ACN (B). Trapping of the peptides was for 4 min at a flow rate of 100 μL/min. A linear gradient (15–40% over 4 min at a flow rate of 40 μL/min) was used for separation on the analytical column. A quadrupole time-of-flight mass analyzer (Synapt G2-Si HDMS QTOF, Waters) with positive ion electrospray ionization tuned for collision-induced dissociation and lock-mass correction (using the leucine enkephalin peptide, 556.2771 *m*/*z*) was used for detection of the peptides. Waters HDMS^E^ mode (from 50 to 2000 *m*/*z*) for 3D (LC, IM, *m*/*z*) was used for obtaining the mass spectra. Settings of the instruments included a capillary voltage of 3.0 kV, a cone voltage at 50 V, a trap collision energy of 4 V, a traveling wave ion mobility separation with 575 m/s, a 36.5 V wave amplitude, and a 2.75 mbar N_2_. Two collision energy settings were used, a transfer collision energy of 4 V for low-energy scans and four separate ramps between 15 and 55 V for high-energy scans.

### Data Analysis

ProteinLynx Global Server 3.03 (PLGS) (Waters) was used to identify peptides discoverable during analysis. Deuterium incorporation was determined with DynamX 3.0 (Waters), with a manual review of all assignments. The intrinsic chemical exchange rate was calculated and simulated for each peptide.^28,34,35^ Furthermore, an in-house MATLAB code was developed for automatic calculation of the segment averaged protection factors as a measure of the reduced exchange brought by the structure of the protein, Figure S1. The code involves three steps: generating intrinsic uptake curves published using values from Englander and co-workers,^34^ fitting them into one or two stretched exponentials as needed,^36^ given the results from an F-test, and plotting, fitting, and filtering the experimental uptake curves in the same manner,^37^ explained in detail in the Supporting Information. The HDX data summary table and HDX data supplementary table are included in the Supporting Information. All uptake curves and fits are also included in the Supporting Information, Figure S13 and Table S1. Structures were modeled in PyMOL (Schrödinger). Other statistical tests were performed with Prism v5.0 (GraphPad).

### Upper Limit for Protection Factors

Owing to the high degree of protection against H/D afforded by the stable core of GlyP, some peptide fragments have very slow HDX kinetics which cannot be robustly fitted. An upper limit for the obtainable ln(Pf) had to be set for this data set. In brief, deuterium incorporation of three average peptides from the data set was plotted with various ln(Pf) according to eq S5. Upon fitting the simulated curves, *R*^2^ of all the fits was plotted against the ln(Pf) (Figure S2C). The upper limit for the obtainable ln(Pf) was determined as an average from three peptides with *R*^2^ corresponding to 0.95, and for this data set, it was determined to be 10 (Figure S2C, as described in the Supporting Information).

## Results and Discussion

### Establishing GlyP a and Phosphorylase b Activity/Inactivity under Deuterium Labeling Conditions

**Figure 1:**
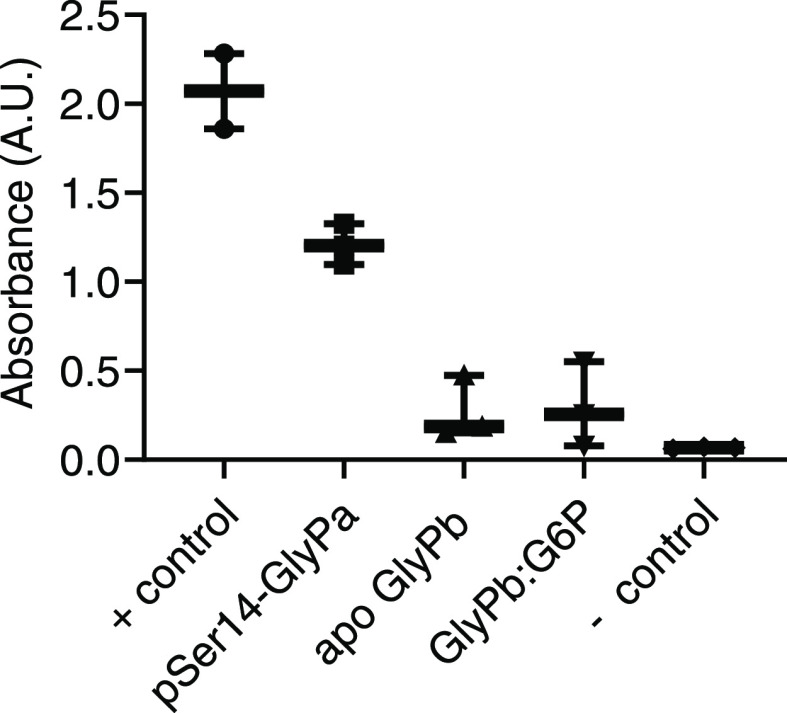
Activity of glycogen phosphorylase states (GlyPa, GlyPb apo, and GlyPb/G6P complex) 50 min from addition of glycogen. Results shown are box plots of absorbance at 450 nm with *n* = 3 presented.

It was crucial that we first establish the activity of GlyPa, GlyPb, and GlyPb-G6P. Only then, under the same conditions, deuterium labeling experiments could inform on the structural dynamics of the protein. A rapid colorimetric assay was used to corroborate the activity/inactivity of both enzymes amid HDX experiment conditions. Compared to the positive control, GlyPa had 60% of its absorbance at 50 min from the start of the reaction (i.e., at saturation), and the apo-GlyPb and inhibited GlyPb/G6P complex had 13 and 14%, respectively (Figure 1). This confirmed that the activity of GlyPa was sufficient under labeling conditions and both GlyPb and GlyPb/G6P complex remained inactive under the same conditions.

### Solution-Phase Local Stability and Structural Perturbation in the Apo-GlyPb Dimer

**Figure 2:**
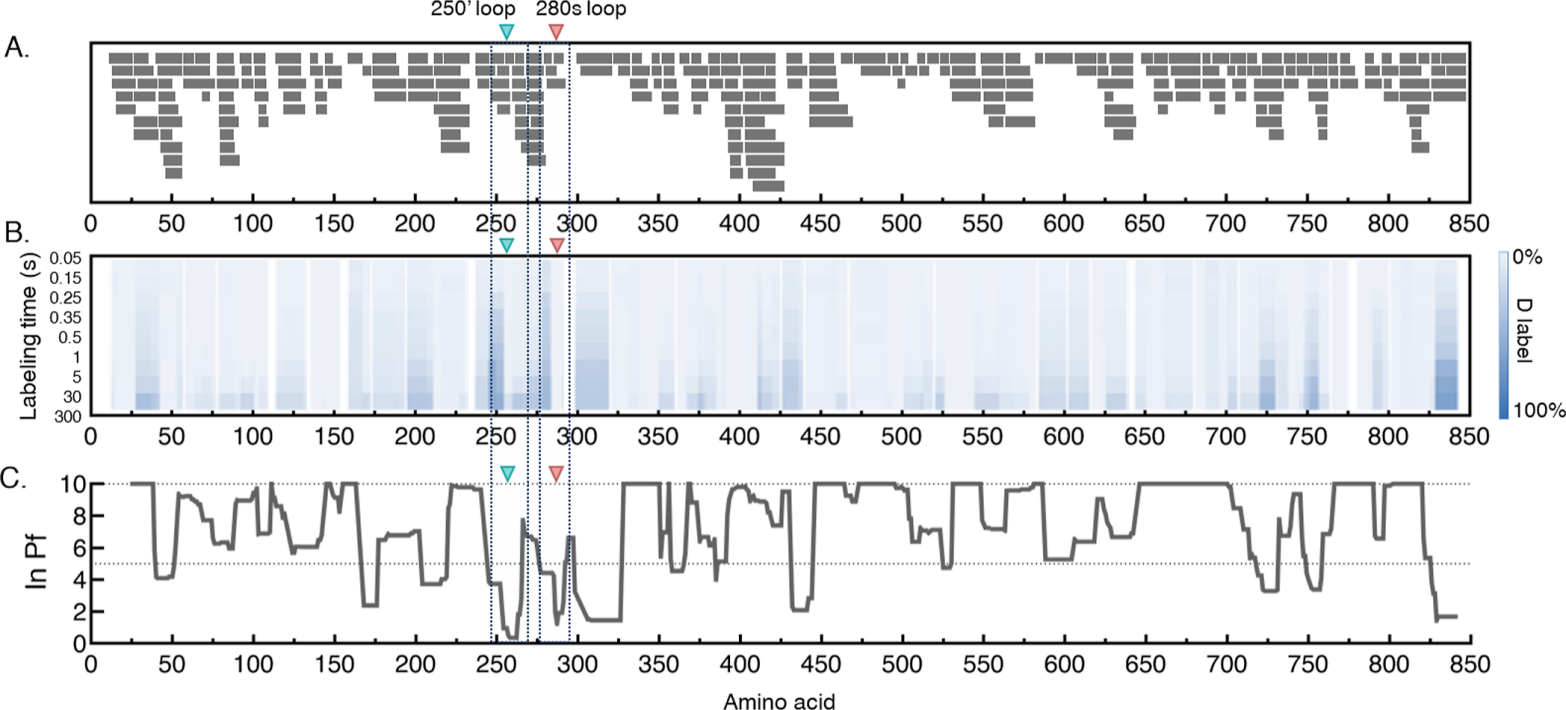
A) Coverage map of GlyP. (B) The heat map indicates the extent of the hydrogen exchange at each labeling time (rows), given as relative fractional uptake of deuterium (%). Peptide-level data has been averaged per amino acid with linear weighting. (C) Average hydrogen-exchange protection factors per amino acid. Up to two protection factors were determined for each peptide (black bars in panel A) and weighted by the fraction of the peptide exchanging under each regime. Gaps in the data were linearly interpolated. Upper limit of quantitation (ln(Pf) = 10).

Upon confirming the activity/inactivity of each state of the enzyme, we sought to determine the localized changes in the stability of the *R*/*T* forms of GlyP in solution. Structurally, GlyP forms a dimer of a large (97 kDa)^8,38^ polypeptide chain which represents a major challenge to quantitatively link local changes in structure and stability in response to its many regulatory influences. Our approach, as established above, stands to provide this link, but is dependent on high data density which results in high structural resolution. 273 unique peptides were identified and monitored with the ms2min system, corresponding to 96.4% linear sequence coverage (Figure 2A). Importantly, coverage was obtained in several significant allosteric regions, including the nucleotide site, catalytic site, tower helix, and both 250′ and 280s loop, Figure 2. Average redundancy reflects the number of overlapping peptides detected and provides high spatial resolution. For this analysis, we achieved 4.08 redundancy, which provided a very high structural resolution. For such a large dataset (three protein conditions each comprising 273 peptide fragments measured at 10 deuterium labeling times, together with simulated uptake data—a total of 10,920 kinetic curves), it is important that all data acquisition and nonlinear regression are automated. Thus, a schedule of ms2min labeling experiments of GlyP in three conditions and from 50 ms to 5 min labeling was acquired and analyzed in a fully automated manner, as explained in the Supporting Information

We characterized the local stability of GlyP in weakly structured regions (i.e., not the folded core which has HDX half-lives > hours and was determined as outside the limit of detection Figure S2C) by measuring the structural dynamics in solution at physiologically relevant pH (7.0). Previously, it was determined that the subunit interface of the dimer has two main contact regions on opposite sides of the enzyme: one between the cap region (residues 35–46) and the α1-α2 loop, β7, and the α2′ helix (47–78) of the opposite subunit.^5^ The other contact is an antiparallel association of the two tower helixes, α7, and the immediately adjacent structural elements. The overall level of exchange in the apo-GlyP T-state is low, consistent with a natively folded and rigid enzyme structure, Figures S8 and S9.

The pattern of HDX protection factors matches well with the crystallographic dimer interface, Figure 2B–C, with no additional large protection factors that would indicate tetramer,^39^Figures S3 and S4. As GlyPb is highly structured and rigid, and this form of analysis has a limit of detection for ln(Pf), a limit had to be established. Since a large percentage of the peptides showed very high ln(Pf), the ln(Pf)limit > 10 was applied prior to describing the structural dynamics.

With a detailed map of local perturbations in the apostate of GlyPb established, we then examined perturbations upon activation/inhibition. The entirely automatic approach to obtain protection factors and free energy of stability was applied to GlyP in two allosterically regulated states: active GlyPa phosphorylated at Ser14 in the *R*-state and inactive GlyPb bound at the nucleotide site to the glucose-6-phosphate inhibitor (GlyP/G6P) in the *T*-state. As GlyPb is highly stable, the largest HDX uptake differences of the two analyzed states were observed at longer time points, >10 s, as was shown previously.^21^ Nevertheless, utilizing the ms2min instrument provided more sensitive quantification of structural differences, and crucially, it allowed calculation of quantitative changes in local stability induced by phosphorylation and inhibitor binding. This was particularly beneficial in several regions such as the 250′ and 280s loops and α8 helix, which undergo large changes in structure and stability between R/T-states, yet have little information content by HDX-MS >10 s.

### Direct Local Stabilization of the Apo/Inactive State by Glucose-6-Phosphate (G6P) Binding to the Nucleotide Site

Binding of G6P to the apo state stabilizes the inactive *T*-state, which is highly populated in the apoprotein; thus, their activities are comparable. Overall, binding of the allosteric inhibitor glucose-6-phosphate^40^ to the nucleotide allosteric site resulted in only local changes in structure and stability, with some exceptions of long-range minor alterations to helices, α11, Figure S5–S7, and S10. The highest protection was observed within residues 38–50 and 305–324, both segments involved with the binding of G6P.

**Figure 3:**
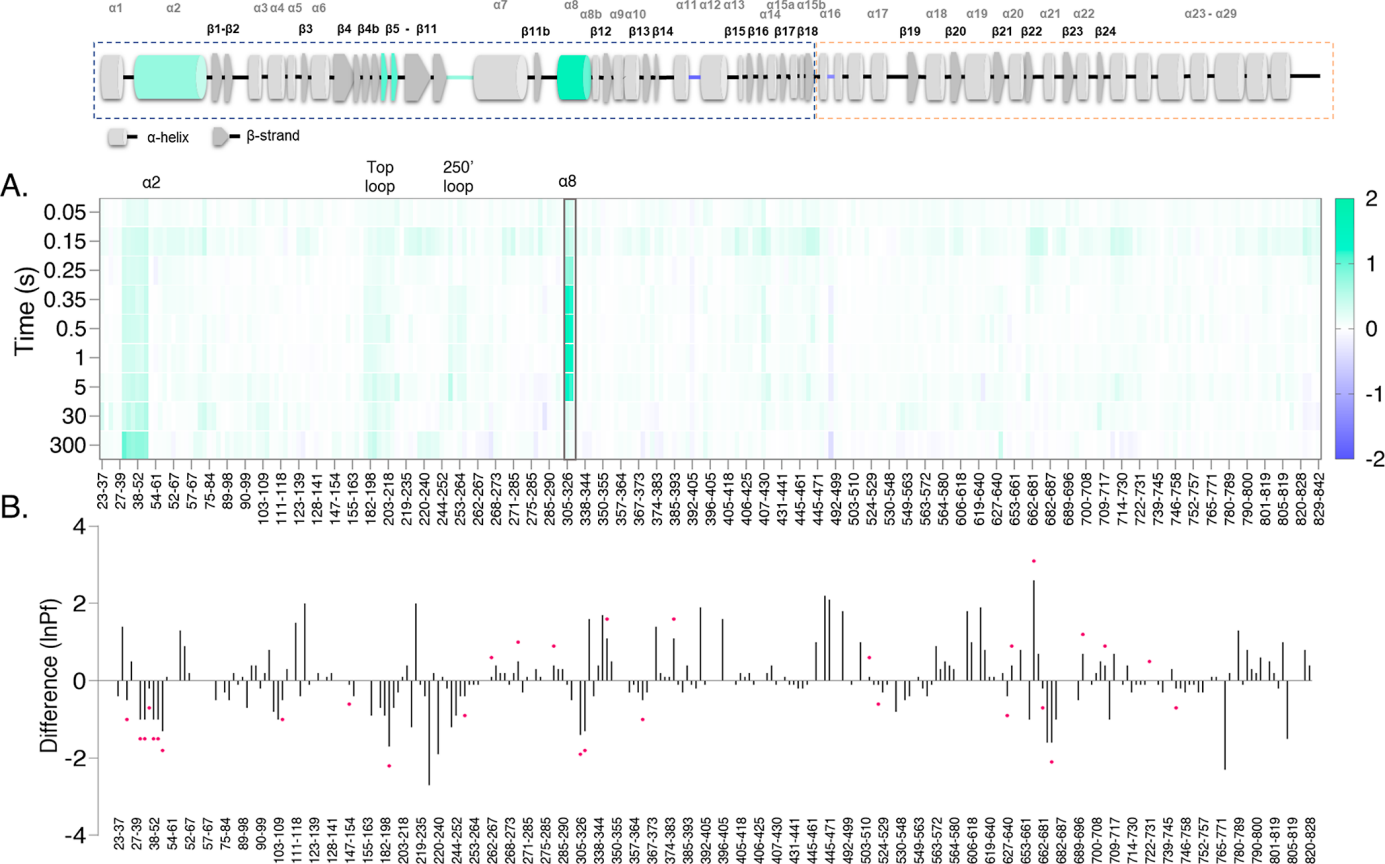
GlyPb and GlyPb/G6P complex-state difference maps. (A) Difference of the observed deuterium uptake between GlyPb and GlyPb/G6P. The difference is represented as relative fractional uptake. The data per time point of the GlyP/G6P complex sample was subtracted from the data for apo *T*-state. Relative protection leads to a more positive value (green); deprotection (e.g., solvent exposure/loss of H-bonding) results in a more negative value (purple). (B) Difference in the calculated ln(Pf) between GlyPb and the GlyPb/G6P complex. Similarly, the calculated protection factor data of the GlyP/G6P complex was subtracted from the data for apo. Relative protection leads to a more negative value (bar below); deprotection results in a more positive value (bar above). The horizontal scale represents each mapped peptide (not all labeled due to limit in space), from the start residue (left) to the end (right). The red asterisks denote a significant difference between the peptides (hybrid significance test methods described in the Supporting Information). Secondary structure assignments from 1GPB.pdb (GlyPb apo) are shown above, with major protection/deprotection mapped by colors purple/green. Note that the *x*-axes for A and B are not identical.

**Figure 4:**
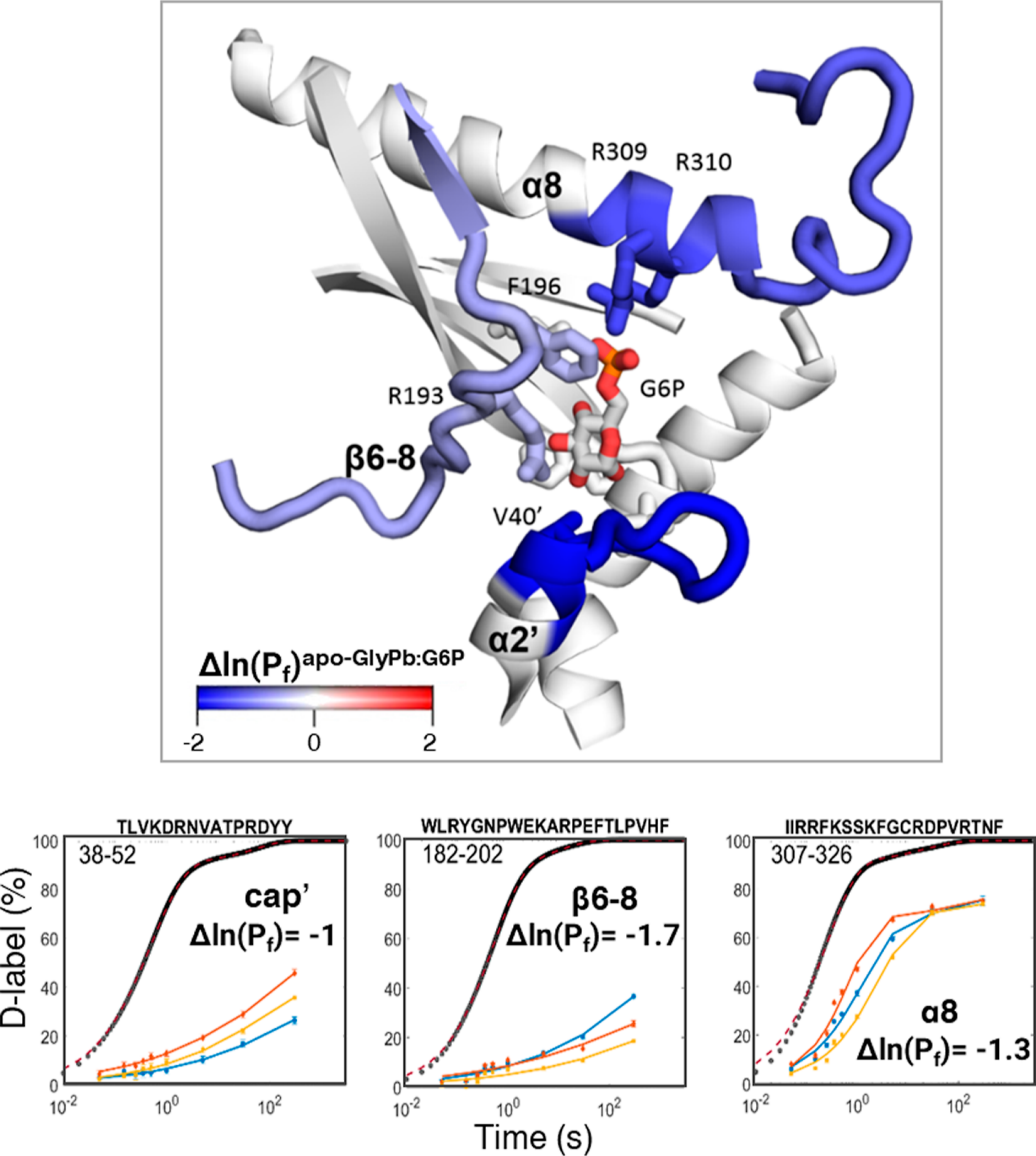
The footprint on the polypeptide chain of bound G6P is discernible from the HDX-MS data yet is more diffuse than might be anticipated. The α1′-α2′ loop is considerably stabilized by Δln(Pf)_peptide_ = −1, even though there is only one contact between G6P and the opposing monomer in the cocrystal structure, Figure S11. All stability values are represented as Δln(Pf)_peptide_, estimated from HDX-MS rates (see the Supporting Information). Plots: apo-GlyPb (red); pSer14-GlyPa (blue); GlyPb/G6P (yellow); theoretical maximum HDX rate for unstructured polypeptide (black traces).

The G6P interface overlaps the AMP binding site^41^ located within the nucleotide allosteric site at a subunit–subunit interface near the C-terminus, Figure 3A–B. The α8 helix and the preceding 280s loop showed the largest changes in stability, with a stabilization of Δln(Pf)_peptide_ = −1.3 of the C-terminus of α8 and the preceding 280s loop, Figure 4. A notable HDX uptake difference of 1.7 Da was identified in the peptide containing residues Arg309 and Arg310, which play a crucial role in the G6P allosteric site interactions, as shown in the crystallographic model of the binding epitope, Figure 3. Despite this, the results showed that the stability of the α8 helix (residues 307–326) remained unchanged within the conventional measurement time frame, in agreement with prior studies.^21^ This suggests that part of the binding epitope for G6P, as determined by crystallography, is not captured in HDX-MS data when measured within the standard time window (i.e., > 10 s).

These findings suggest that the N-terminus of the α8 helix and the preceding 280s loop are highly sensitive to allosteric modulation and that the Arg309 and Arg310 residues play a crucial role in these structural changes.

This may represent direct mediation of allostery from the nucleotide site via the rigid α8 helix to the active site, although it is unclear how the partial release of the 280s entropic gate can manifest as inhibitory. The impact of this change in 280s stability is not immediately clear and warrants further study, in particular by comparison with the effects of AMP, but it is attractive to consider that it may be a necessary conflation of the dual functions of the nucleotide site which binds to activating and inhibiting allosteric regulators.^42−44^ The footprint of bound G6P on the polypeptide chain is discernible from the HDX-MS data, yet it is considerably more diffuse than might be anticipated, based on the crystallographic contacts, Figures S10 and S11. Notably, only one interchain (van der Waals) contact is identified from the cocrystal structure (between the G6P sugar ring around O2 and Val40′ of the opposing subunit), yet there is extensive protection against hydrogen exchange observed throughout the a1′-a2′ loop, stabilizing these amino acids by Δln(Pf)_peptide_ = −1, Figures 4 and S10. This indicates that the loop has considerable conformational flexibility in the apo-form that is significantly constrained by the contacts formed upon ligand binding, namely, van der Waals contacts with G6P and with Ile68 and Trp69. Though perhaps expected, no amide hydrogen perturbation is observed in the beta sheet at the rear of the nucleotide site, or in α3, which both consist of extensive H-bonding networks.

### Phosphorylation at Ser14 Induces the Well-Established Switch in Conformation from the T- to R-State

**Figure 5:**
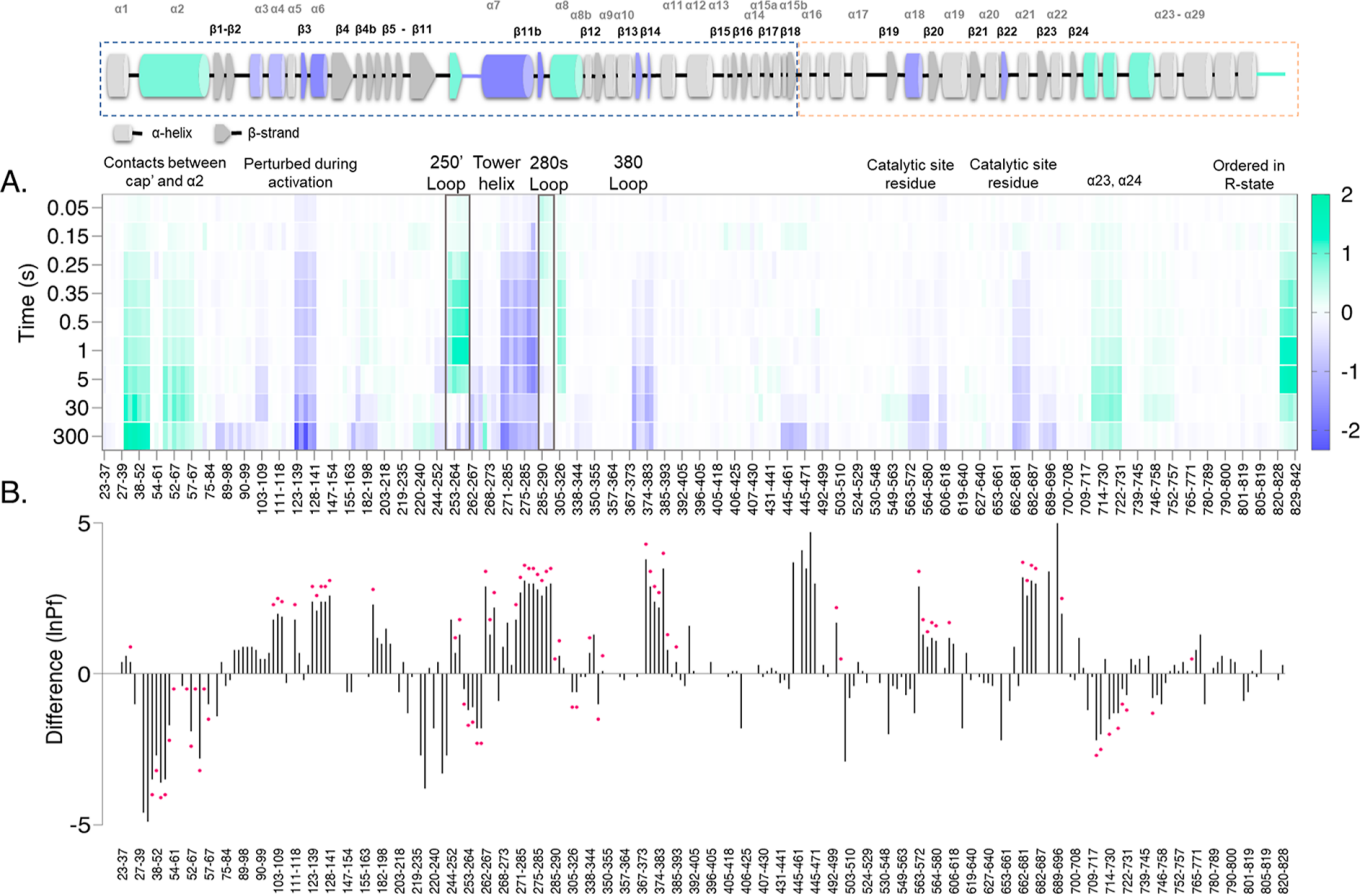
GlyPb and GlyPa complex-state difference maps. (A) Difference in the observed deuterium uptake between GlyPb and GlyPa. The difference is represented as relative fractional uptake. The data per time point of the GlyPa sample was subtracted from the data for the apo *T*-state. Relative protection leads to a more positive value (green); deprotection (e.g., solvent exposure/loss of H-bonding) results in a more negative value (purple). (B) Difference in the calculated ln(Pf) between GlyPb and GlyPa. Similarly, the calculated protection factor data of the GlyPa complex was subtracted from the data for apo. Relative protection leads to a more negative value (bar below); deprotection results in a more positive value (bar above). The horizontal scale represents each mapped peptide (not all labeled due to limit in space), from the start residue (left) to the end (right). The red asterisks denote a significant difference between the peptides (hybrid significance test methods described in the Supporting Information). Secondary structure assignments from 1GPB.pdb (GlyPb apo) are shown above, with major protection/deprotection mapped by colors purple-green. Note *x*-axis for A and B are not identical.

**Figure 6:**
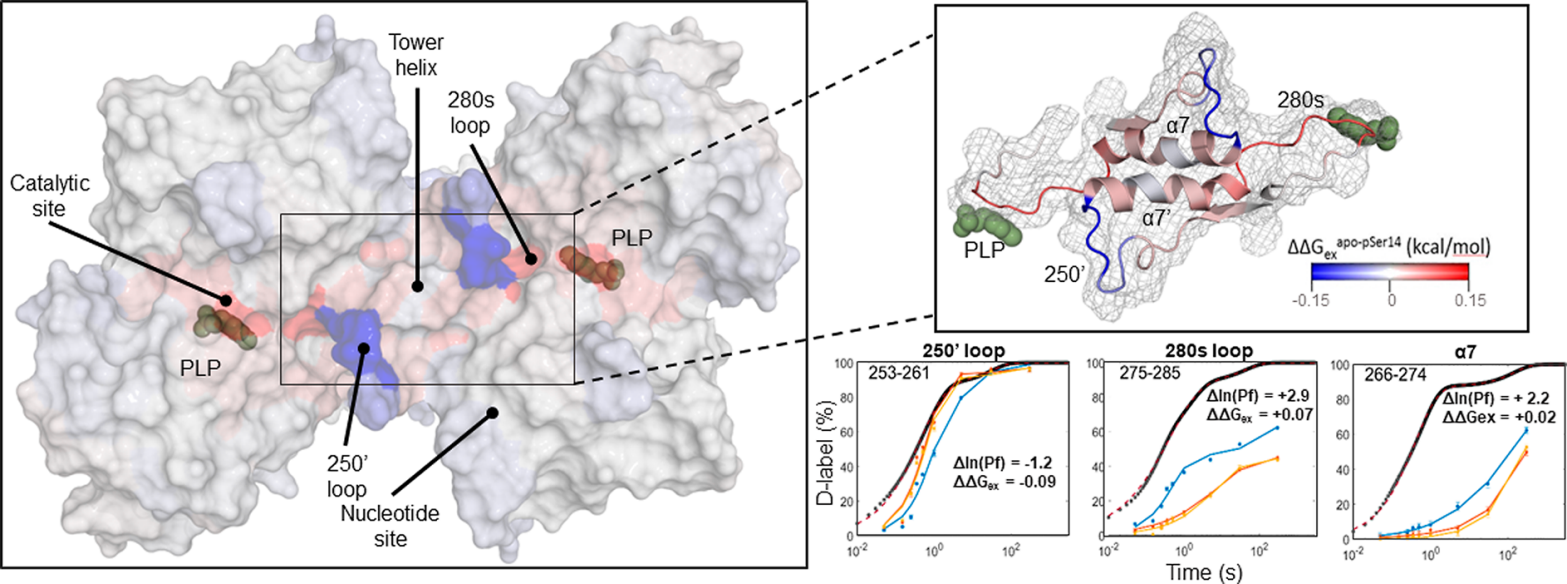
The tower helix region appears to behave as a gate with stabilization in the 250′ loop induced by enzyme activation (here Ser14 phosphorylation), coupled with minor destabilization in the tower helix and large destabilization in the 280s loop at the entrance to the catalytic site. Inset: detail of the tower helix and flanking loops with estimated changes in per amino acid stability upon activation (kcal/mol/aa calculated from HDX-MS rates). Uptake plots: apo-GlyPb (red); pSer14-GlyPa (blue); GlyPb/G6P (yellow); theoretical maximum HDX rate for unstructured polypeptide (black traces), derived automatically from the in-house-developed code.

Phosphorylation at the N-terminus (pSer14) induces the well-established flip in local conformation from the *T*-state to *R*-state.^45^ Crucially, the 280s loop (residues 278–289) was missing electron density in the original crystal structures of the R-state.^5^ This led to the Barford–Johnson hypothesis that an order/disorder transition by the 280s loop directly regulates access to the active site. The active and inactive states have notable structural differences, described in detail previously, with the major changes known to occur in the catalytic site,^46,47^ the nucleotide site,^48^ and the tower helix^6,8^ (Figures 5 and 6; S5–S7). We calculated local stability changes in these regions from millisecond deuterium labeling data for the GlyPb and GlyPa states (Figure 5).

Several significant regions show large deuterium uptake and consequently ln(Pf) differences between the *R*/*T*-states. Overall, these segments include residues 38–52, 52–70, 250–260, and 700–713 which are highly protected upon activation, and residues 128–141, 260–285, 280–290, 370–380, and 570–590 which are deprotected. We describe the solution-phase stability changes in these significant regions in detail below.

Upon activation, the tower helix α7 (residues 261–276) alters its angle upward 10° relative to the long axis of the dimer—increasing solvent accessibility—and unfolds a partial turn into the 250′ loop (residues 248–260). This necessarily breaks certain stable contacts with the tower helix’ of the other subunit.^49^ Stability changes are observed in solution, consistent with this (Figures S8 and S9): the tower helix stabilizes by Δln(Pf)_peptide_ = −1.8 (an HDX uptake difference of 1 Da at <350 ms) equivalent to an estimated change in local stability of ΔΔ*G*_ex_ of −0.1 kcal/mol/aa at the N-terminal end of the helix (Tyr261-Gln263) and destabilizes by Δln(Pf)_peptide_ = 3 (HDX difference of −1 Da), corresponding to an estimated ΔΔ*G*_ex_ of +0.04 kcal/mol/aa at the C-terminal end (Ala272-Ile275). The 250′ loop preceding α7 is found to be natively disordered (unstable) in the apo-form but is significantly stabilized in GlyPa by Δln(Pf)_peptide_ = −1.2, corresponding to a ΔΔ*G*_ex_ of −0.04 kcal/mol/aa. In particular, the 250′ loop shows the difference in the exchange kinetics only during labeling times of 0.25–5 s—not before or after (Figure S13 for residues 257–264). Thus, conventional HDX methodologies may fail to capture this critical regulatory behavior as only a minimal difference in uptake data can be detected within the conventional timeframe (>10 s). The inverse relationship is seen at the other end of the tower helix in the 280s loop. It appears that it was correctly assumed by Johnson and co-workers that “these residues...become mobile” upon transition to the phosphorylated *R*-state. The relatively stable loop in the apo *T*-state is destabilized by +0.07 kcal/mol/aa.^50,51^ The exchange kinetics of the 280s loop provide a comprehensive insight into its structural behavior upon activation. Even at shorter exposures, for example, 50 ms, an HDX uptake difference of 0.5 Da is observed, but no significant difference is observed at labeling times >350 ms, Figure 5A.

This provides direct solution-phase data to support the model that the mechanism by which the 280s loop gates access the catalytic site is by a change in its weakly stable structure. Moreover, it quantifies the relative changes that occur upon enzyme activation. The reciprocal relationship between the stability of the 280s and 250s′ loops supports an extension to the hypothesis, whereby these loops, connected by the rigid yet mobile tower helix, act together upon activation.^12^

GlyPa exhibits extensive destabilization throughout the catalytic site, Figures 5 and 6. Largest differences were observed in α6 (Δln(Pf)_peptide_ = 2.6), β13 (Δln(Pf)_pept3ide_ = 3.8), and the β22-α21 loop (Δln(Pf)_peptide_ = 2), Figure S12.

Several of these affected amino acids make direct contact with the pyridoxal phosphate (PLP) cofactor and so they may contribute to reorientation for productive catalysis. This allosteric effect is long-range: 57 Å from the phosphorylated N-terminus to beyond the PLP. Several of the observed sites within these PLP-contacting loops are shielded from solvent in the crystal model, indicating that a considerable dynamic rearrangement of the PLP-polypeptide interactions must occur to result in hydrogen exchange.

The cap’ (residues 35–46) and α2 (residues 47–78) interface showed smaller changes than elsewhere in the allosteric transition between GlyPb and GlyPa; however, this interface is somewhat stabilized in the active conformation by an average of Δln(Pf)_peptide_ = −3, Figure S12. The cap’ and α2 have been observed to come closer together^49^ and, in line with that, we see the solution-phase HDX-MS protection is increased in those regions, Figures S7–S9.

## Conclusions

It is well established that intrinsically disordered proteins require labeling times in the millisecond range to accurately quantitate structural dynamics differences by HDX. However, here, we show that this is also the case for structural motifs that critically regulate the active site of GlyP, a 195 kDa globular enzyme. Reliable quantitation of exchange rates was achieved by accurate measurement with deuterium labeling times spanning several orders of magnitude—from 50 ms to 5 min.

Activation of GlyP by phosphorylation at Ser14 results in large allosteric perturbations spanning almost 6 nm to the far side of the catalytic site. Multiple loops that contact the PLP cofactor exhibited increased hydrogen exchange. Much of the solvent accessibility of these loops is via the deep catalytic site itself, which requires that these dynamic changes result in a reorganization of PLP orientation in the pocket, with the implication that PLP is held in place somewhat loosely.

The 280s loop is absent in some crystal models of the R-state. This has given rise to the hypothesis that it acts as a gate for access to the active site: ordered/structured in the T-state which blocks glycogen entry and disordered/unstructured in the R-state which permits glycogen to access the deep catalytic pocket. Here, we quantitatively reinforce this hypothesis by calculating the relative local changes in Gibbs free energy of stability from hydrogen/deuterium-exchange measurements of the GlyP dimer in solution. Moreover, we observed data consistent with an extension to this hypothesis, proposing that the tower helix acts as a lever to couple the order/disorder of the 280s loop with disorder/order in the 250′ loop.

Importantly, this experimental approach of using millisecond HDX-MS to measure site-localized stability enables a detailed quantitative understanding of transitional mechanisms in large and complex protein systems. The ability to measure weakly structured local regions should be important in testing other hypotheses for protein allostery in natively folded and intrinsically disordered proteins more broadly.

## Supporting Information Available

Data from this study will be made available upon request. The Supporting Information is available free of charge at https://pubs.acs.org/doi/10.1021/acs.biochem.2c00671.

HDX-MS summary and processed uptake data (XLSX)
Additional method details (HDX-MS data analysis and calculations, code for Pf analysis); flowchart of the in-house MATLAB code developed; prediction of the upper limit for the obtainable Pf; heat maps of differential deuterium uptake; protection coverage maps of plotted log (Pf), difference maps, difference in protection factors; heat maps of the difference in HDX labeling between different states, protection factors per amino acid for GlyP in three states; estimates of the Gibbs free energy of stability Δ*G*_ex_(HDX) per amino acid in GlyP in three states; estimates of the change in free energy of stability ΔΔ*G*_ex_(HDX) per amino acid; interactions of the G6P inhibitor with GlyP; catalytic and allosteric site transitions; HDX analysis of GlyP per peptide; and fitting parameters to the multiphase stretched exponential (PDF)

## Accession Codes

The GlyP accession code is P00489 (Uniprot).
