# Supplementary material for: Allosteric Regulation of Glycogen Phosphorylase by Order/Disorder Transition of the 250′ and 280s Loops
Source: Biochemistry. 2023 Mar 29;62(8):1360–8. doi: 10.1021/acs.biochem.2c00671 (PMC10116597; doi:10.1021/acs.biochem.2c00671)
Supplement: Supplementary file 2 — bi2c00671_si_002.pdf [file bi2c00671_si_002.pdf]

Supporting Information

Allosteric regulation of glycogen phosphorylase by order/disorder transition of the 250' and 280s loops

Monika Kish[1], Sivaraman Subramanian[2], Victoria Smith[3], Natasha Lethbridge[3], Lindsay Cole[4], Frank Vollmer[2], Nicholas. J. Bond[5] and Jonathan J. Phillips[*1,6]

[1]Living Systems Institute, Department of Biosciences, University of Exeter, Stocker Road, Exeter, EX4 4QD, UK

[2]Living Systems Institute, Department of Physics, University of Exeter, Stocker Road, Exeter, EX4 6QD, UK

[3]CPI, Darlington, DL1 1GL, UK

[4]Applied Photophysics Ltd, Leatherhead, KT227BA, UK

[5]Analytical Sciences, Biopharmaceutical Development, BioPharmaceuticals R&D, AstraZeneca, Milstein Building, Granta Park, Cambridge, CB21 6GH, UK

[6]Alan Turing Institute, British Library, London, NW1 2DB, UK

*Correspondence to: jj.phillips@exeter.ac.uk

Table of contents, figures and tables

SI Materials and Methods 2

Data analysis 2

Kinetic analysis 2

SI Results 5

Figure S1. Flow chart of the step-by-step explanation of the in-house Matlab code developed for automatic calculation of the segment averaged protection factors.. 3

Figure S2 Prediction of the upper limit for the obtainable Pf.. 5

Figure S3. Heat maps of differential deuterium uptake for active pSer14-GlyPa, apo T-state and GlyP:G-6-P complex in T-state.. 6

Figure S4. Protection coverage maps of plotted log (Pf) for active pSer14-GlyPa, apo T-state and GlyP:G-6-P complex in T-state.. 7

Figure S5. Difference maps. 8

Figure S6. Difference in protection factors between GlyP in active R-state and apo T-state and between inactive inhibitor-bound state and apo T-state.. 9

Figure S7. Heat maps of the difference in HDX labeling between different states: active pSer14-GlyPa minus apo and GlyP:G6P minus apo.. 10

Figure S8. Protection factors calculated per peptide and plotted per amino acid (from mean average of overlapping peptide segments) for GlyP in three states: apo T GlyP, pSer14 GlyP and GlyP:G6P inactive complex.. 11

Figure S9. Estimates of the Gibbs free energy of stability ΔG_{ex}(HDX) calculated per peptide and plotted per amino acid (from mean average of overlapping peptide segments) in GlyP in three states: apo T-state, pSer14 GlyPa and GlyP:G6P inactive complex.. 12

Figure S10. Estimates of the change in free energy of stability $\Delta\Delta G_{ex}$(HDX) per amino acid in GlyP upon activation by phosphorylation and inhibition by G6P 13

Figure S11. Interactions of G6P inhibitor with GlyP, from 1GPY.pdb. 13

Figure S 12. The catalytic site is extensively remodelled upon transition to the R-state by phosphorylation at Ser14. Perturbations in HDX-MS measured rates were detected in several of the loops that contribute to catalytic activity and bind PLP. 14
Figure S 13. HDX analysis of GlyP per peptide. 14

Table S1 Fitting parameters to multi-phase stretched exponential for hydrogen/deuterium-exchange mass spectrometry of GlyP in apo, activated and inactivated enzyme. 21

SI Materials and Methods

Data analysis. Subsequent data filtering, fitting, normalization, and protection factors calculation was done using in- house programs in MatLab (MathWorks).

Kinetic analysis. An in-house Matlab code was developed for automatic calculation of the segment averaged protection factors (*Pf*) as a measure of the reduced exchange brought by the structure of the protein. The code involves three steps: generating intrinsic uptake curves, fitting them into one- or two stretched exponentials, and plotting and fitting the experimental uptake curves in the same manner. Firstly, the intrinsic chemical amide exchange rates were calculated and simulated for each peptide according to equation 1 as demonstrated previously by Bai et all and adapted from the excel sheet provided by Englander lab (available online here http://hx2.med.upenn.edu/).

$$D(t) = \sum_{i=2}^{n}\left(1 - e^{-k_{int}^{(i)}t}\right)$$

Equation S1

Where n is the number of residues in each peptide, k_{int} is the intrinsic rate constant of chemical exchange for each residue and t is the labeling time. This equation uses the sum of exponentials for each amide in a peptide to provide the degree of deuterium incorporation as a function of a labeling time. At the N-terminus of the peptide the first amide becomes a primary amine after proteolysis, thus the first residue back-exchanges quickly during the LC-MS analysis. As a result, index n starts from the second residue onwards. Proline does not contain an amide hydrogen, and so its rate constant will always be zero. We adapted all the calculations from the excel spreadsheet available on W. Englander's website. From these we generated and simulated the uptake curves for each peptide, at the experimental pH and temperature[1, 2].
The exchange kinetics were quantified as it was described previously, where the theoretical and experimental deuterium uptake were fit to a single- or double-stretched exponential function.

$$D(t) = Q\left[1 - e^{-((k)t)^{\beta}}\right]$$

Equation S2

$$D(t) = Q_1\left[1 - e^{-((k_1)t)^{\beta}}\right] + Q_2\left[1 - e^{-((k_2)t)^{\beta}}\right]$$

Equation S3

Where *D(t)* is the deuterium uptake as a function of the labelling time t, Q is the number of exchangeable amides, k represents the segment-averaged exchange constant, and β is an exponential stretching factor that accounts for the distribution of the exchange rates of the individual amides. The stretched exponential function is used when fitting the H/D kinetics, as it requires less adjustable parameters than the commonly used multi-exponential one. To confirm the suitability and aptness of the stretched exponential models, we performed an F-test on the one- and two-stretched exponential models. If the more complicated (two-stretched exponential) model is correct, then the relative increase in the sum-of-squares would be greater than the relative degrees of freedom ($F > 1$, $p < .0005$). Thus, only when it is statistically necessary the two-stretched exponential model will be selected.

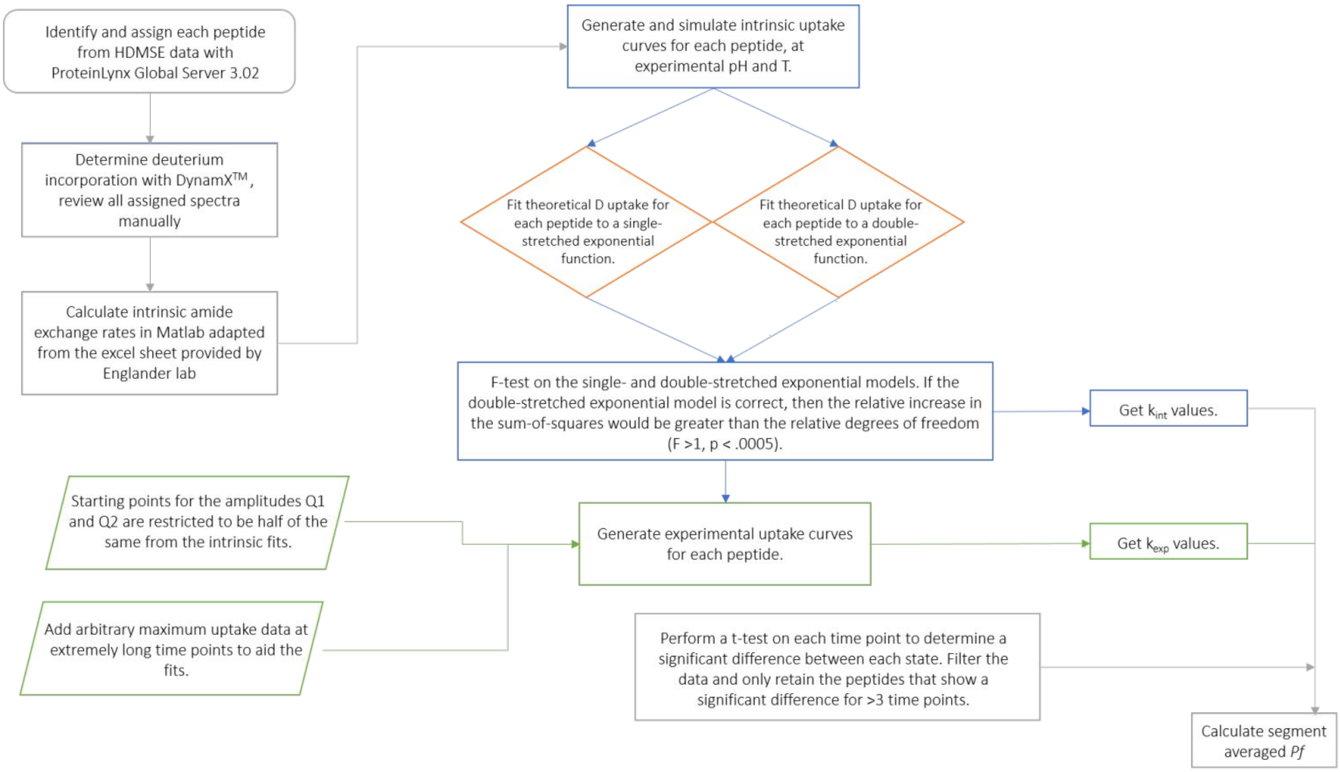

Figure S1. Flow chart of the step-by-step explanation of the in-house Matlab code developed for automatic calculation of the segment averaged protection factors (Pf).The experimental uptake curves were then fitted in the same manner as the intrinsic curves, where the starting points for the amplitudes Q1 and Q2 were restricted to be half of the previously determined Q1 and Q2 from the intrinsic fits. Also, it was necessary to add arbitrary maximum uptake data at extremely long time points to aid the fits. This facilitated the correct fit particularly for the protected regions of the protein that are exchanging predominantly slow. If the double-stretched exponential model was correct the corresponding k was calculated as an average and used for calculating protection factors.

The experimental data was afterwards filtered, thus only the peptides showing significant differences were considered[3]. Then, a t-test was performed for each time point for each peptide, between two selected states. Only when (3) or more time points showed significant difference the peptide would be accepted for further data analysis, as explained in Figure S1.
The segment averaged protection factors can be then estimated by using the ratio of the intrinsic exchange rate constant (k_{int}) to the measured (experimental) rate constant (k_{exp}).

$$Pf = \frac{k_{int}}{k_{exp}}$$

Equation S4

Where *Pf* = protection factor against hydrogen exchange, k_{int} = intrinsic amide hydrogen exchange rate constant from published values[2], k_{exp} = fitted exchange rate constant for back-exchange corrected experimental data using Equation S2 or Equation S3.

An upper limit for the obtainable *Pf* had to be set, and for this data set it was determined to be 10. After analyzing the data set, 3 average peptides, with various averaged k_{int} were chosen. The intrinsic deuterium incorporation was simulated after fitting the data acquired from the excel sheet by Bai et all according to Equation S5. Different curves were simulated with increasing *Pf,* Figure S2A. Only the data points corresponding to the experimental data collected in the GlyP data set were extracted, then fitted with the previously explained method (Equation S2), Figure S2B.

$$D(t) = Q[1 - e^{-((k/Pf)t)^{\beta}}]$$

Equation S5

Where *D(t)* is the deuterium uptake as a function of the labelling time t, Q is the number of exchangeable amides,

k represents the segment-averaged intrinsic exchange constant, β is an exponential stretching factor and *Pf* is idealistic protection factor ranging from 0 to 10^6.
The R^2 of all the fits using only time points equivalent to the GlyP experimental data set were plotted against the *Pf* (Figure 2C), and the upper limit for the obtainable *Pf* was determined as an average from 3 peptides with R^2 corresponding to 0.95.

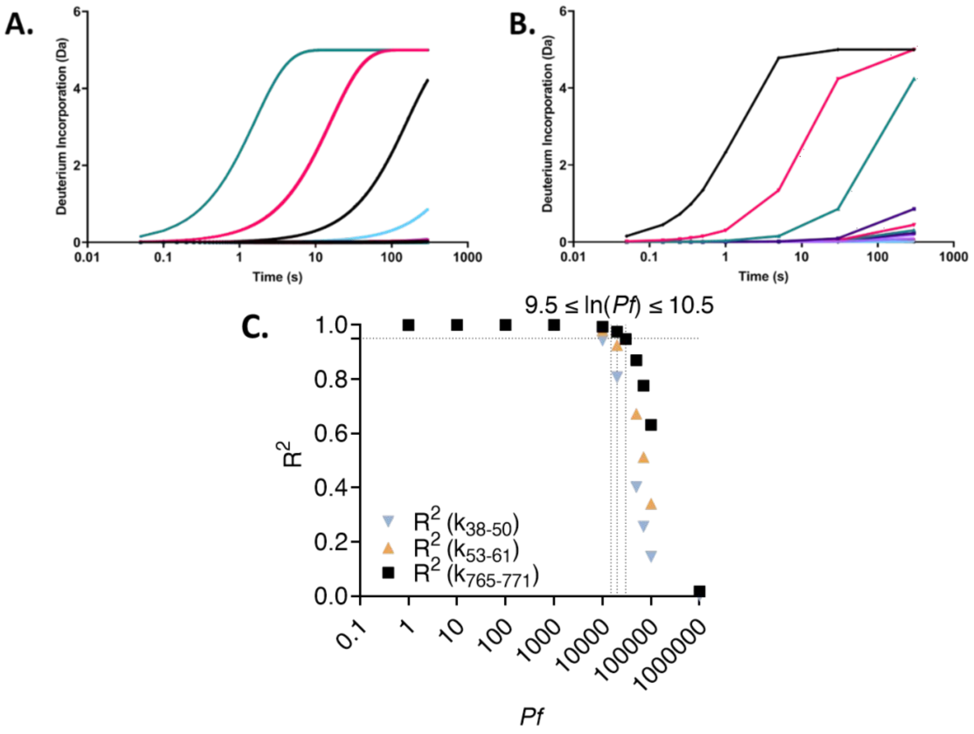

Figure S2 Prediction of the upper limit for the obtainable Pf. A. Simulated intrinsic deuterium incorporation with increasing Pf. B. Extracted data points from the intrinsic deuterium incorporation, corresponding to the experimental data points with the same labelling times as collected for this data set. C. R^2 vs Pf of the stretched exponential fits of the extracted data points for 3 peptide segments for all Pf. The vertical lines correspond to the Pf with R^2=0.95.

Gibbs free energy estimates from HDX-MS data were calculated from the established relationship, Equation S6, for each experimentally observed peptide segment. Below, the figures of *Pf* and Gibbs free energy estimates show non-filtered data for purposes of transparency, but only significantly different peptides were used to calculate $\Delta\Delta G_{ex}$ and are shown on the structural heatmaps (in the manuscript Figures).

$$\Delta G_{ex}(HDX) = -RT\ln\frac{1}{Pf}$$

Equation S6

Where R = gas constant (kcal/K/mol), T = temperature (K) and *Pf* = protection factor from Equation S4.

SI Results

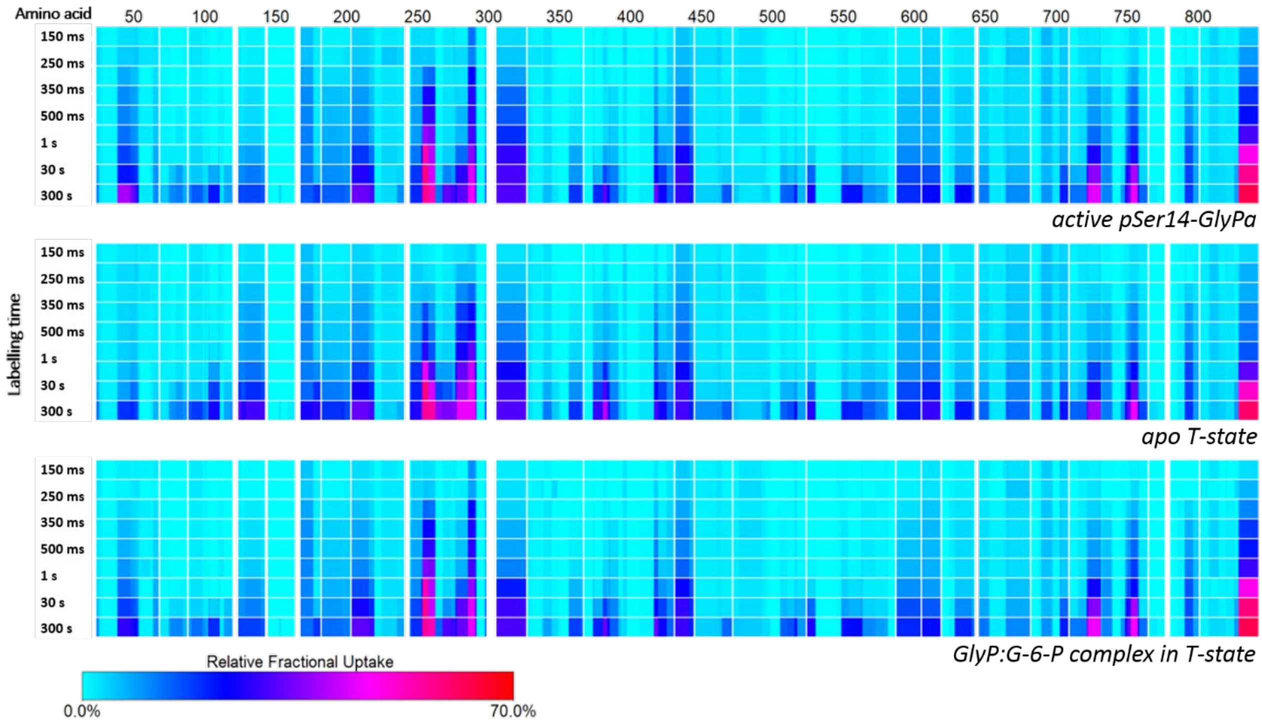

Figure S3. Heat maps of differential deuterium uptake for active pSer14-GlyPa (top), apo T-state (middle) and GlyP:G-6-P complex in T-state (bottom). No back-exchange correction and statistical filtering were performed for the heat map generation, made with DynamX (Waters). The HDX uptake is resolved at amino acid level, at nine time points including 0.05, 0.15, 0.25, 0.35, 0.5, 1, 5, 30, and 300 s from top to bottom. The deuteration level at each time point for each amino acid is color coded shown on left.

Figure S4. Protection coverage maps of plotted log (Pf) for active pSer14-GlyPa (top), apo T-state (middle) and GlyP:G-6-P complex in T-state (bottom). Back-exchange correction and statistical filtering were performed for the coverage map generation, made with in-house developed Matlab code for automatic calculation of protection factors. Secondary structure assignments from 1GPB.pdb are shown above. Note that these may not be precisely located where the x-axis is for assigned peptides, not linear amino acid sequence.

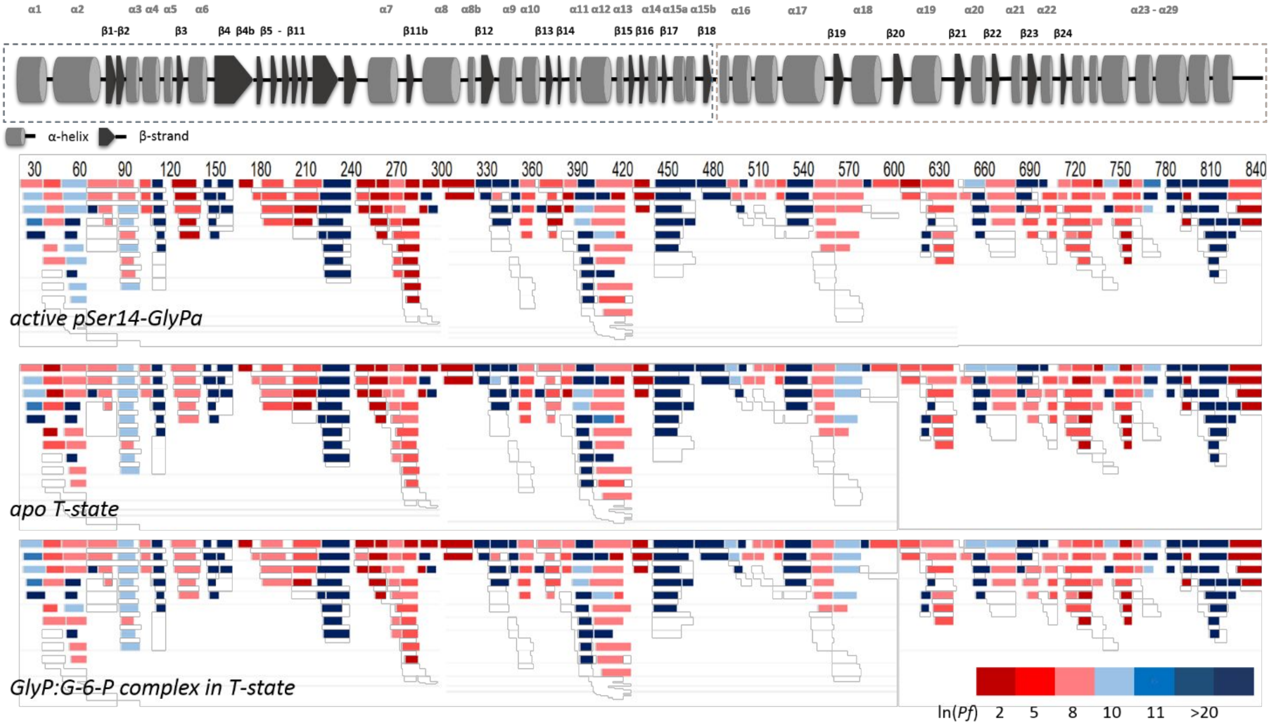

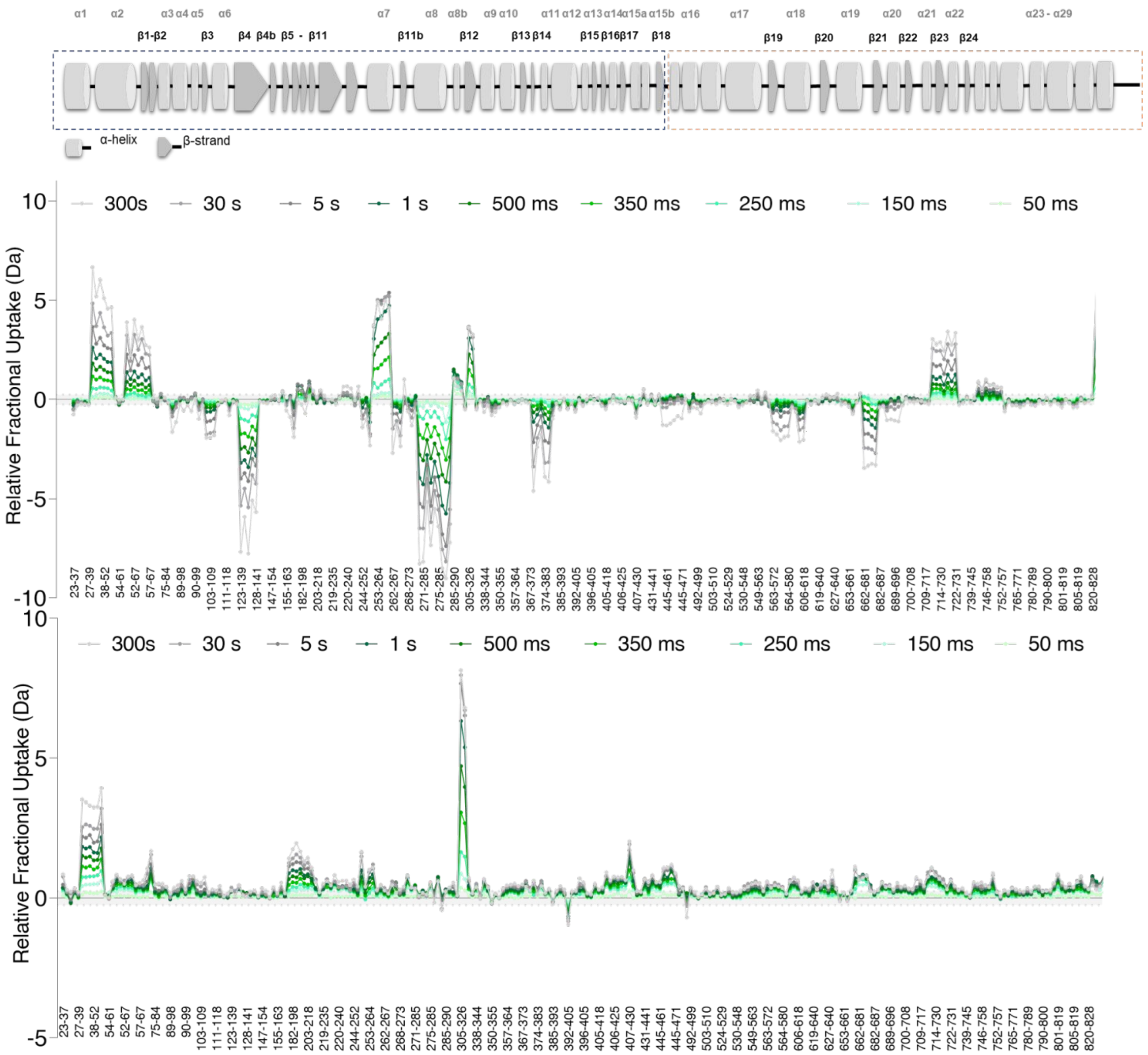

Figure S5. Difference maps. The deuterium uptake data from GlyPa was subtracted from the data for GlyPb in order to create the deuterium uptake difference plot. Relative protection leads to a more positive value (bar on upper side); deprotection (e.g. from an exposed domain interface) results in a more negative value (bar on bottom side) (top). The deuterium uptake data from GlyPb:G6P was subtracted from the data for GlyPb in order to create the deuterium uptake difference plot. Relative protection leads to a more positive value (bar on upper side); deprotection (e.g. from an exposed domain interface) results in a more negative value (bar on bottom side) (top). Grey shaded region on Figure 4B denotes global significant difference as explained previously[3].

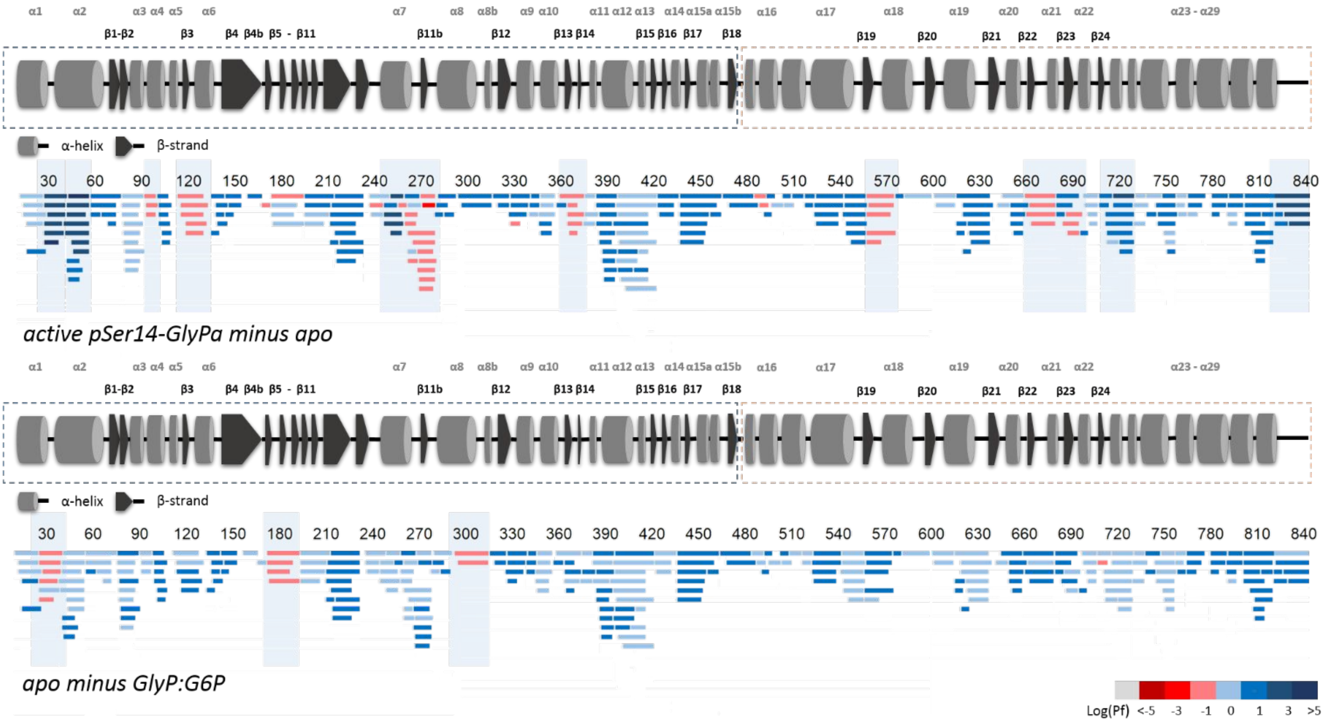

Figure S6. Difference in protection factors between GlyP in active R-state and apo T-state (top) and between inactive inhibitor-bound state and apo T-state (bottom). Each bar under the sequence number represents a peptide fragment monitored during the labeling experiments. Peptides colored according to the measured protection factors, where more positive value indicates more protection against hydrogen-exchange in apo GlyP. Regions of interest with coherent HDX difference are highlighted in shaded boxes. Secondary structure assignments from 1GPB.pdb are shown above. Note that these may not be precisely located where the x-axis is for assigned peptides, not linear amino acid sequence.

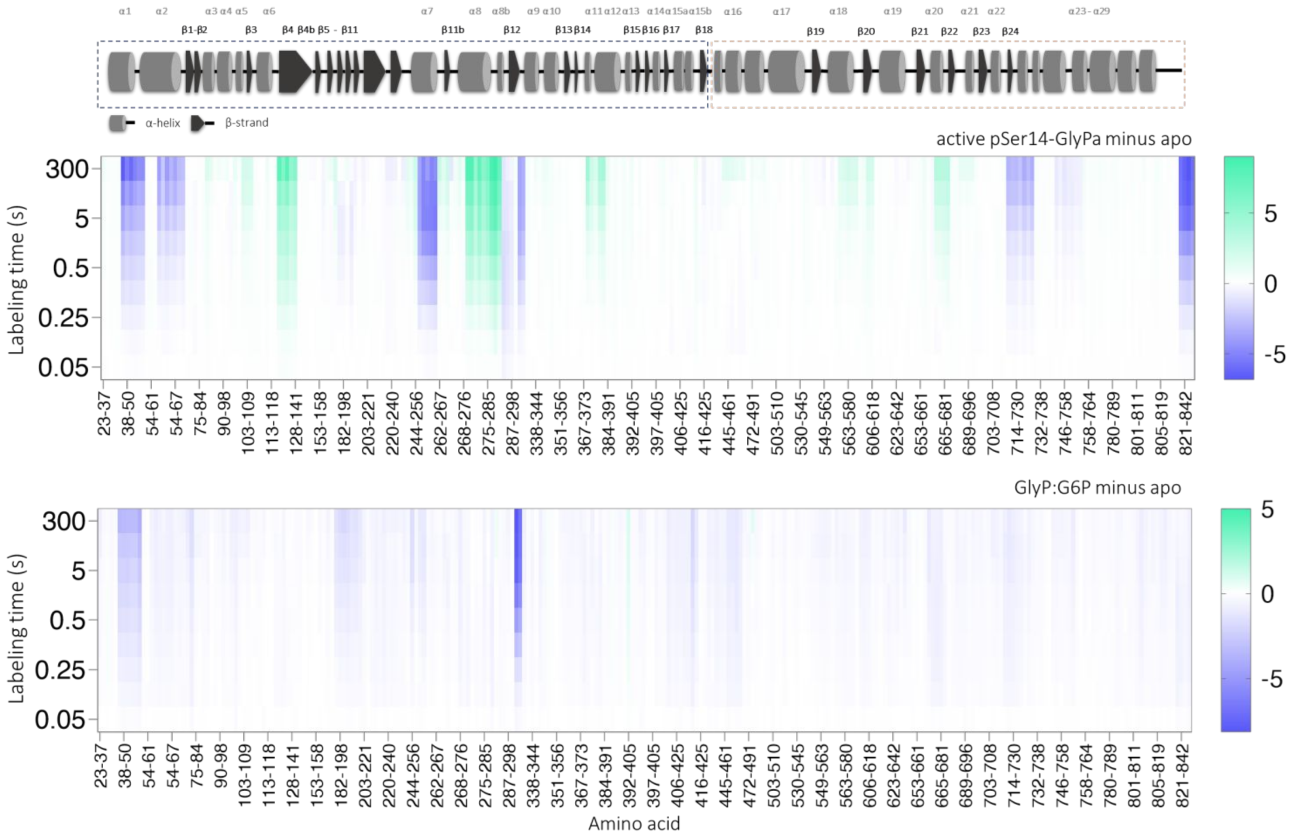

Figure S7. Heat maps of the difference in HDX labeling between different states: active pSer14-GlyPa minus apo (top) and GlyP:G6P minus apo (bottom). The relative deuteration level summed up at each subsequent time point per peptide is color coded shown on the scale below; note different normalisation. Secondary structure assignments from 1GPB.pdb are shown above. Note that these may not be precisely located where the x-axis is for assigned peptides, not linear amino acid sequence.

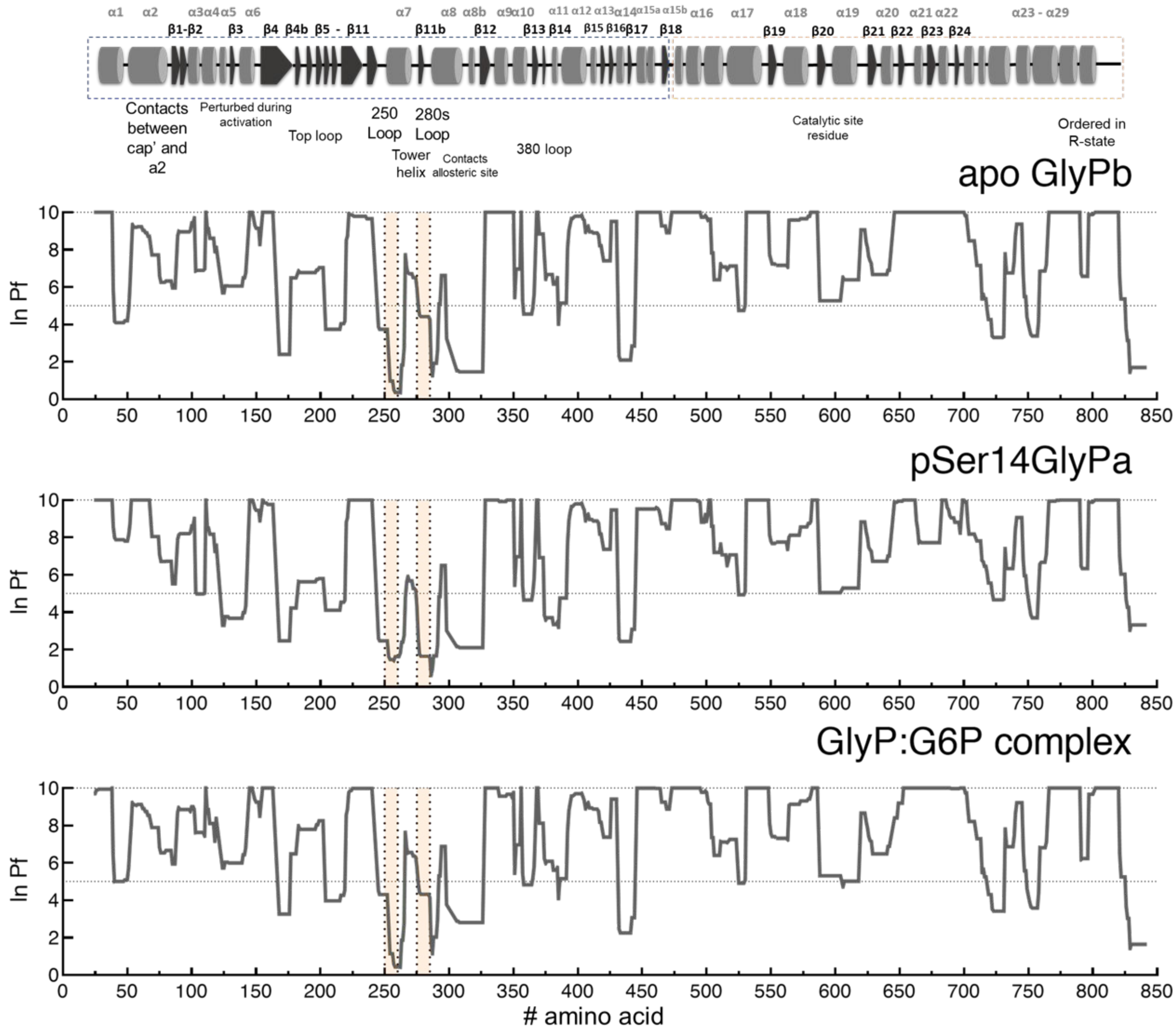

Figure S8. Protection factors calculated per peptide and plotted per amino acid (from mean average of overlapping peptide segments) for GlyP in three states: apo T GlyP (top), pSer14 GlyP (middle) and GlyP:G6P inactive complex (bottom). Values calculated from fitted exchange rate constants using Equation S4. Regions of interest with coherent HDX difference are highlighted in shaded boxes. Secondary structure assignments from 1GPB.pdb are shown above. Data shown is unfiltered per significance.

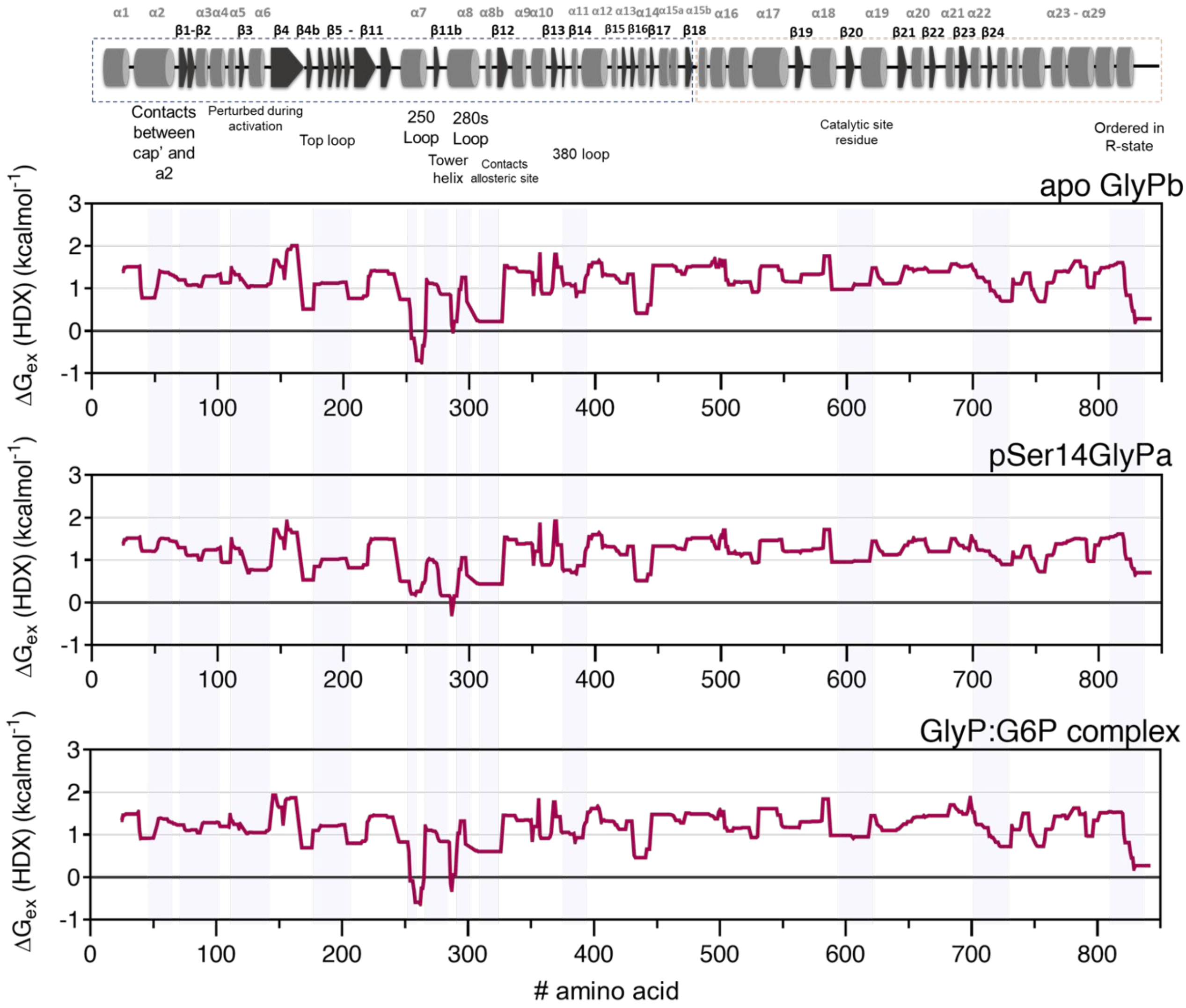

Figure S9. Estimates of the Gibbs free energy of stability ΔG_{ex}(HDX) calculated per peptide and plotted per amino acid (from mean average of overlapping peptide segments) in GlyP in three states: apo T-state (top), pSer14 GlyPa (middle) and GlyP:G6P inactive complex (bottom). Values calculated from fitted exchange rate constants using Equation S6. 2 kcal/mol/peptide is the upper limit of quantitation, given the very slow HDX rates of strongly protected amide protons. Regions of interest with coherent HDX difference are highlighted in shaded boxes. Secondary structure assignments from 1GPB.pdb are shown above. Data shown is unfiltered per significance.

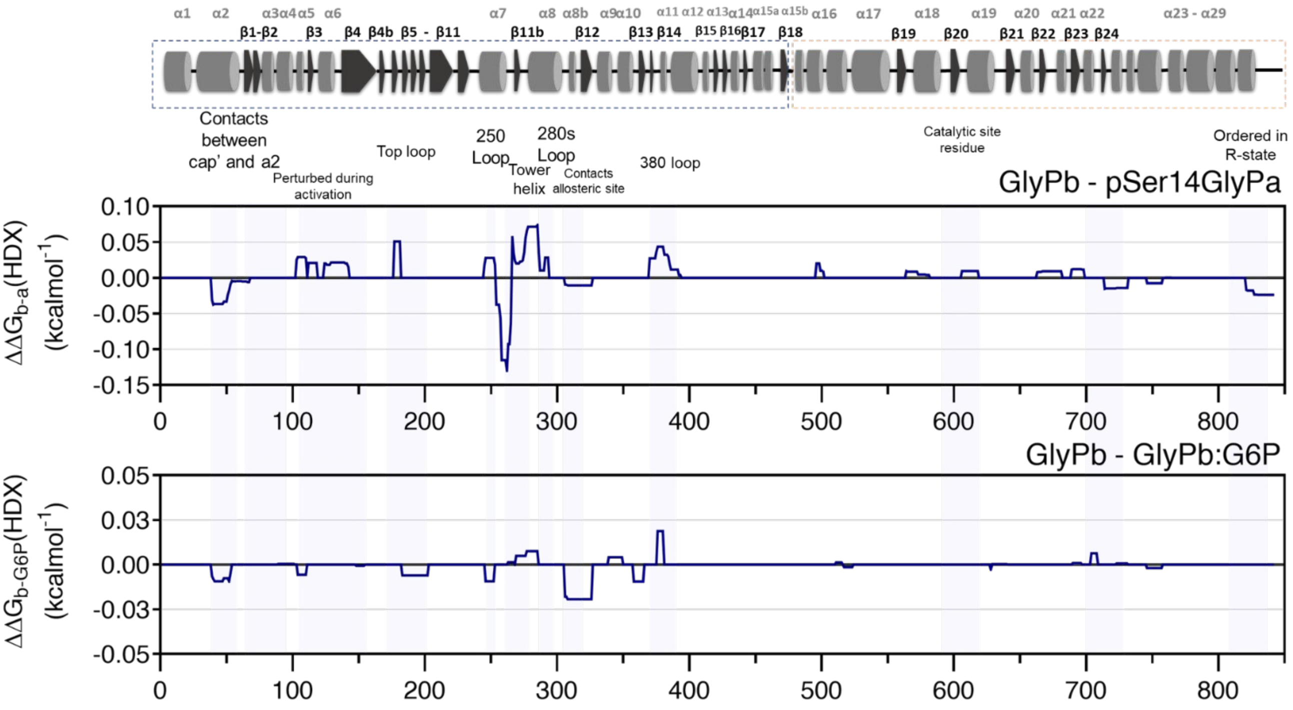

Figure S10. Estimates of the change in free energy of stability $\Delta\Delta G_{ex}$(HDX) per amino acid in GlyP upon activation by phosphorylation (top) and inhibition by G6P (bottom). Regions of interest with coherent HDX difference are highlighted in shaded boxes. Secondary structure assignments from 1GPB.pdb are shown above. Data shown is filtered per significance.

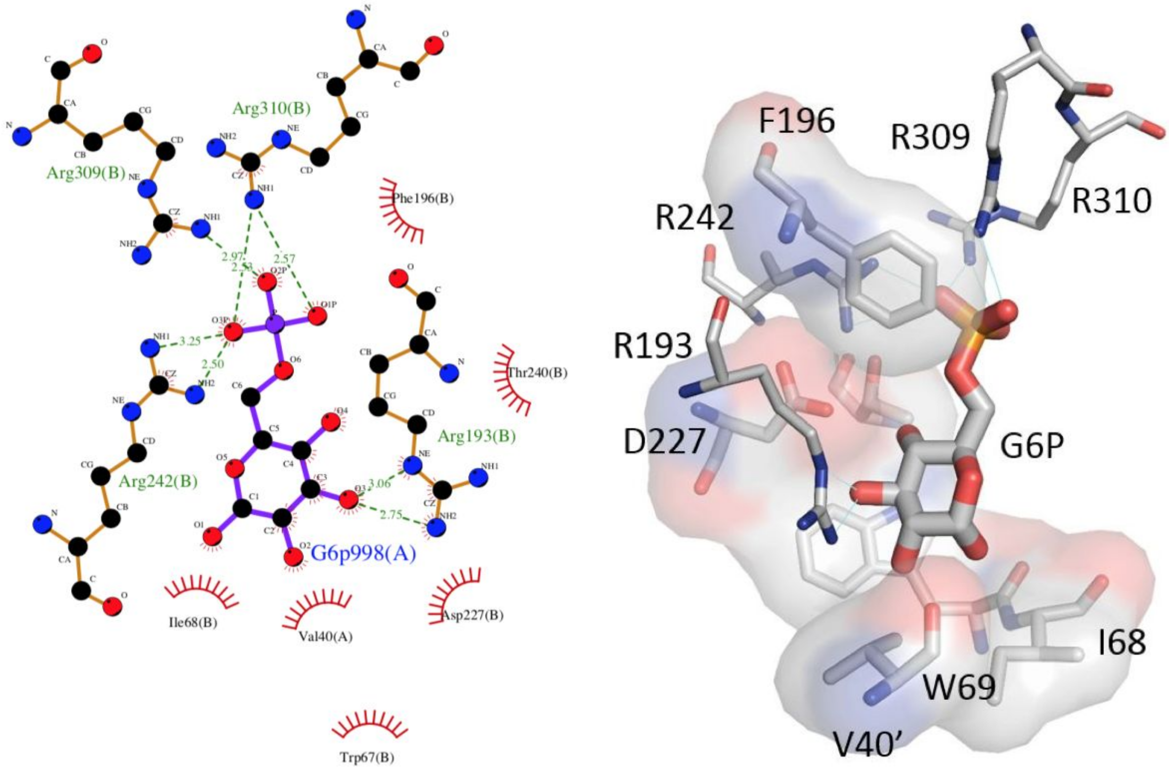

Figure S11. Interactions of G6P inhibitor with GlyP, from 1GPY.pdb. Created with Ligplot+ v2.1 (left) and Pymol (right - Schrodinger). The majority of interactions between G6P and GlyP are with the monomer that forms the nucleotide site and a single van der Waals interaction is identified between the G6P and the opposing monomer at Val40'.

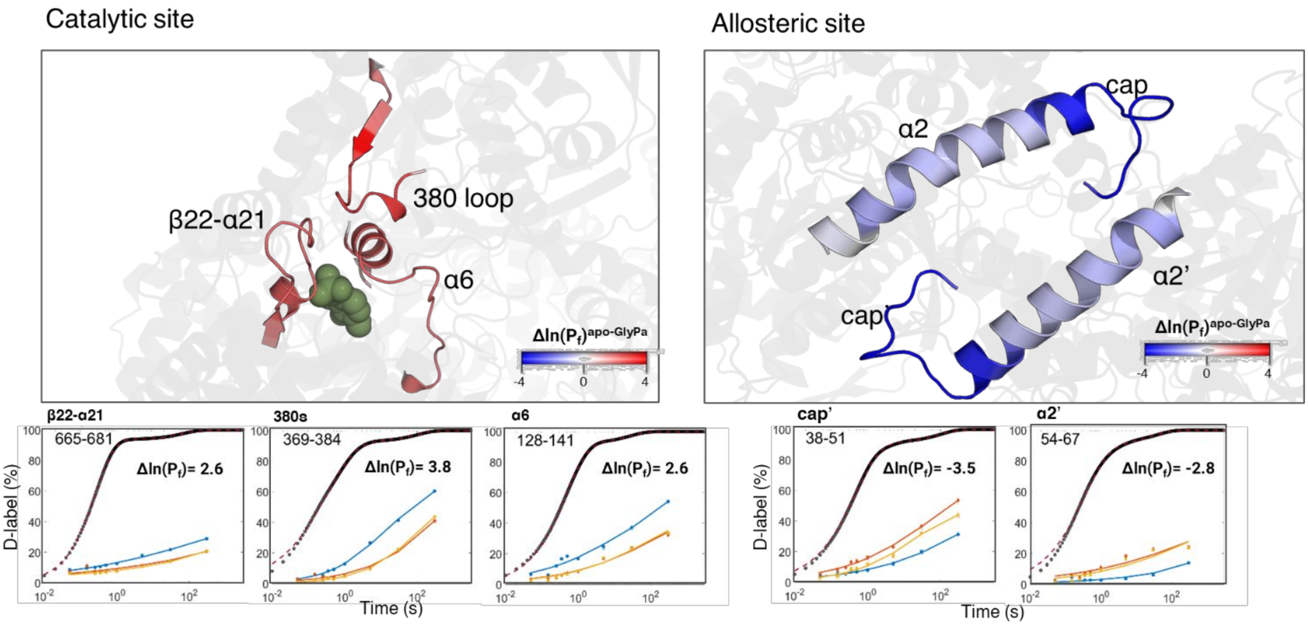

Figure S12. The catalytic site (left) is extensively remodelled upon transition to the R-state by phosphorylation at Ser14. Perturbations in HDX-MS measured rates were detected in several of the loops that contribute to catalytic activity and bind PLP (green). Stabilization was observed in the allosteric site (right), specifically the cap' and α2 interface. All values are represented in $\Delta\ln(Pf)_{\text{peptide}}$, estimated from HDX-MS rates (see Supplementary Information). Uptake plots: apo-GlyPb (red); pSer14-GlyPa (blue); GlyPb:G6P (yellow); theoretical maximum HDX rate for unstructured polypeptide (black traces), derived automatically from the in-house developed code.

Figure S13. HDX analysis of GlyP per peptide. Back exchange corrected data for three protein states shown (red – apo GlyPb; yellow – G6P-bound inhibited GlyPb; blue – pSer14 activated GlyPa) against theoretical intrinsic exchange rates, calculated from [1]. Peptide sequence given above the plot, amino acid number in top-left. Y-axis shows absolute % deuteration at labeling time (s) on X-axis (labels omitted for space).

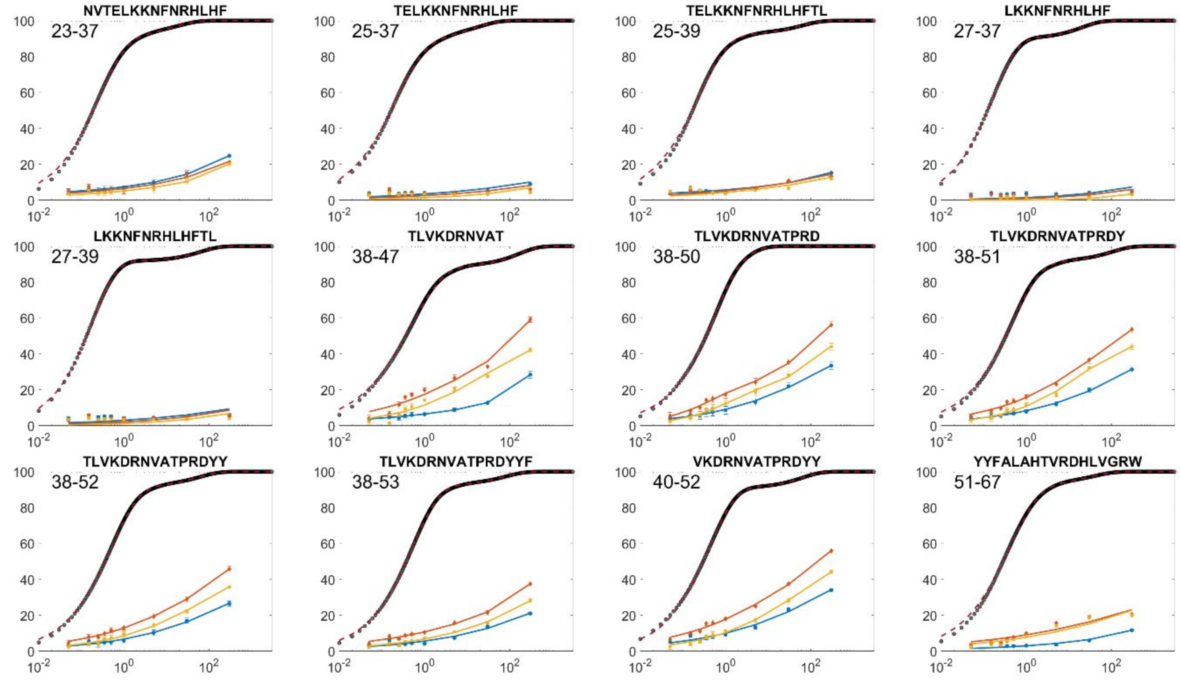

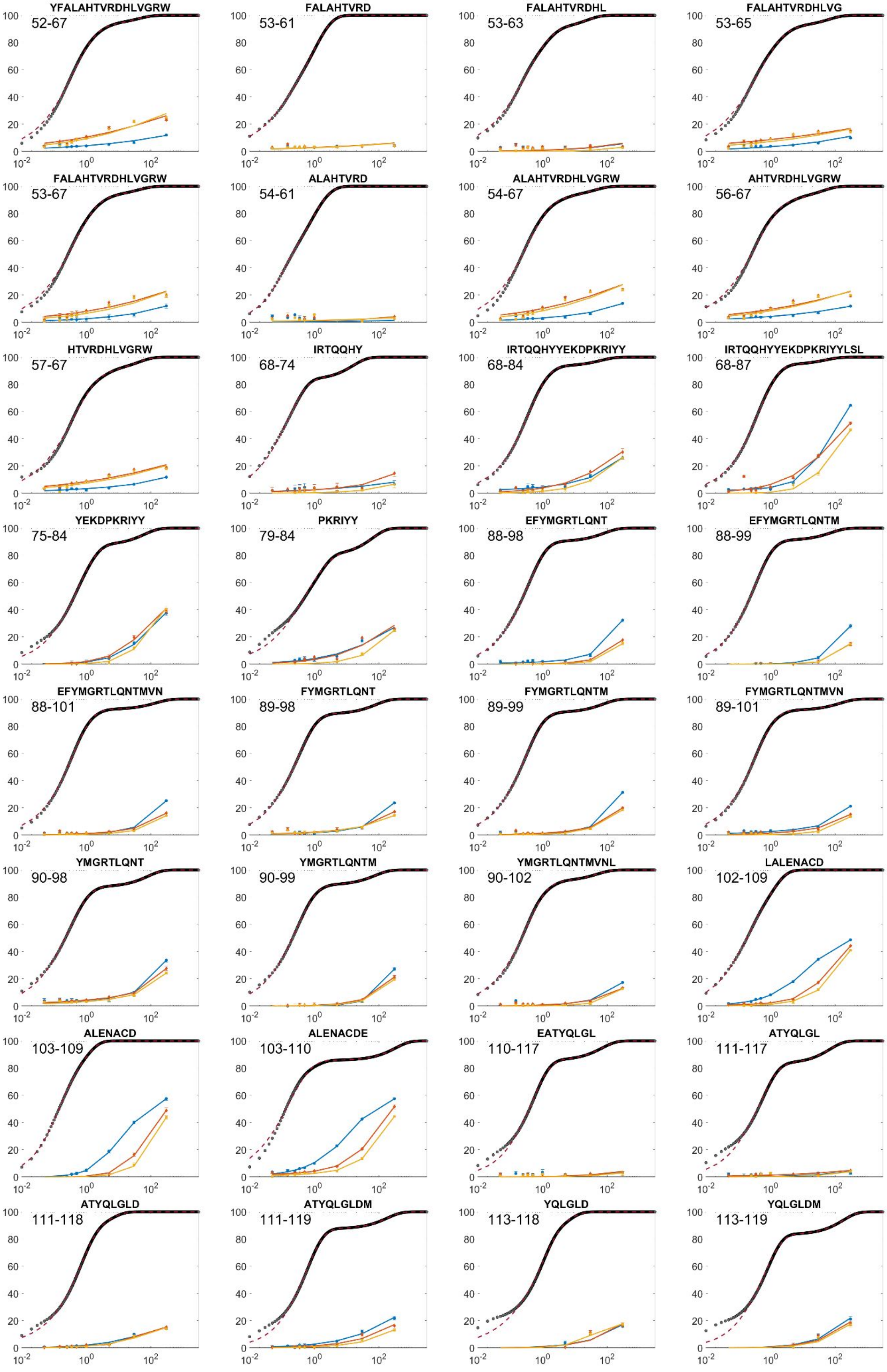
YFALAHTVRDHLVGRW
52-67
FALAHTVRD
53-61
FALAHTVRDHL
53-63
FALAHTVRDHLVG
53-65
FALAHTVRDHLVGRW
53-67
ALAHTVRD
54-61
ALAHTVRDHLVGRW
54-67
AHTVRDHLVGRW
56-67
HTVRDHLVGRW
57-67
IRTQQHY
68-74
IRTQQHYYEKDPKRIYY
68-84
IRTQQHYYEKDPKRIYYLSL
68-87
YEKDPKRIYY
75-84
PKRIYY
79-84
EFYMGRTLQNT
88-98
EFYMGRTLQNTM
88-99
EFYMGRTLQNTMVN
88-101
FYMGRTLQNT
89-98
FYMGRTLQNTM
89-99
FYMGRTLQNTMVN
89-101
YMGRTLQNT
90-98
YMGRTLQNTM
90-99
YMGRTLQNTMVNL
90-102
LALENACD
102-109
ALENACD
103-109
ALENACDE
103-110
EATYQLGL
110-117
ATYQLGL
111-117
ATYQLGLD
111-118
ATYQLGLDM
111-119
YQLGLD
113-118
YQLGLDM
113-119

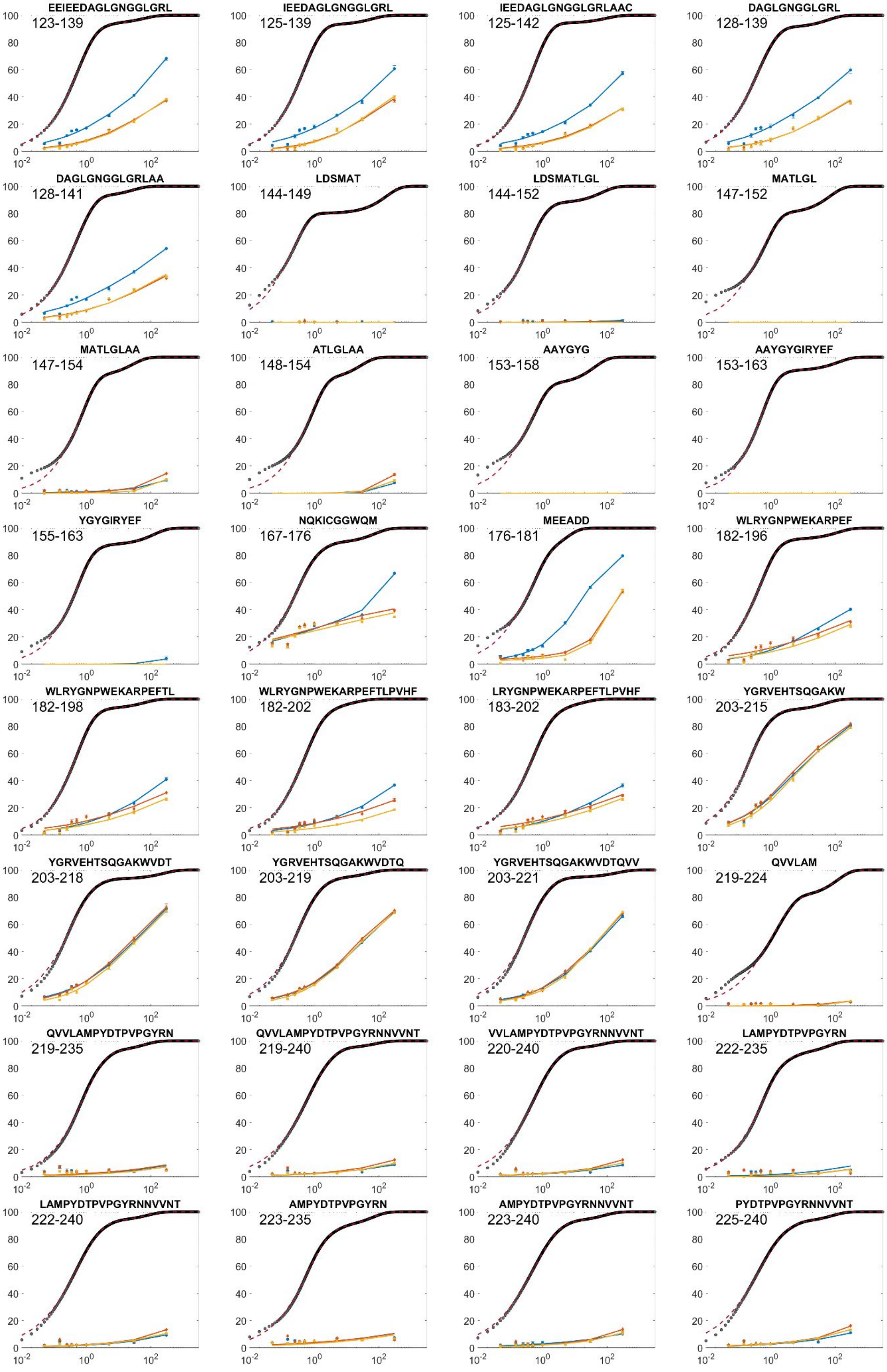
EEIEEDAGLGNGGLGRL
123-139
IEEDAGLGNGGLGRL
125-139
IEEDAGLGNGGLGRLAAC
125-142
DAGLGNGGLGRL
128-139
DAGLGNGGLGRLAA
128-141
LDSMAT
144-149
LDSMATLGL
144-152
MATLGL
147-152
MATLGLAA
147-154
ATLGLAA
148-154
AAYGYG
153-158
AAYGYGIRYEF
153-163
YGYGIRYEF
155-163
NQKICGGWQM
167-176
MEEADD
176-181
WLRYGNPWEKARPEF
182-196
WLRYGNPWEKARPEFTL
182-198
WLRYGNPWEKARPEFTLPVHF
182-202
LRYGNPWEKARPEFTLPVHF
183-202
YGRVEHTSQGAKW
203-215
YGRVEHTSQGAKWVDT
203-218
YGRVEHTSQGAKWVDTQ
203-219
YGRVEHTSQGAKWVDTQVV
203-221
QVVLAM
219-224
QVVLAMPYDTPVPGYRN
219-235
QVVLAMPYDTPVPGYRNNVVNT
219-240
VVLAMPYDTPVPGYRNNVVNT
220-240
LAMPYDTPVPGYRN
222-235
LAMPYDTPVPGYRNNVVNT
222-240
AMPYDTPVPGYRN
223-235
AMPYDTPVPGYRNNVVNT
223-240
PYDTPVPGYRNNVVNT
225-240

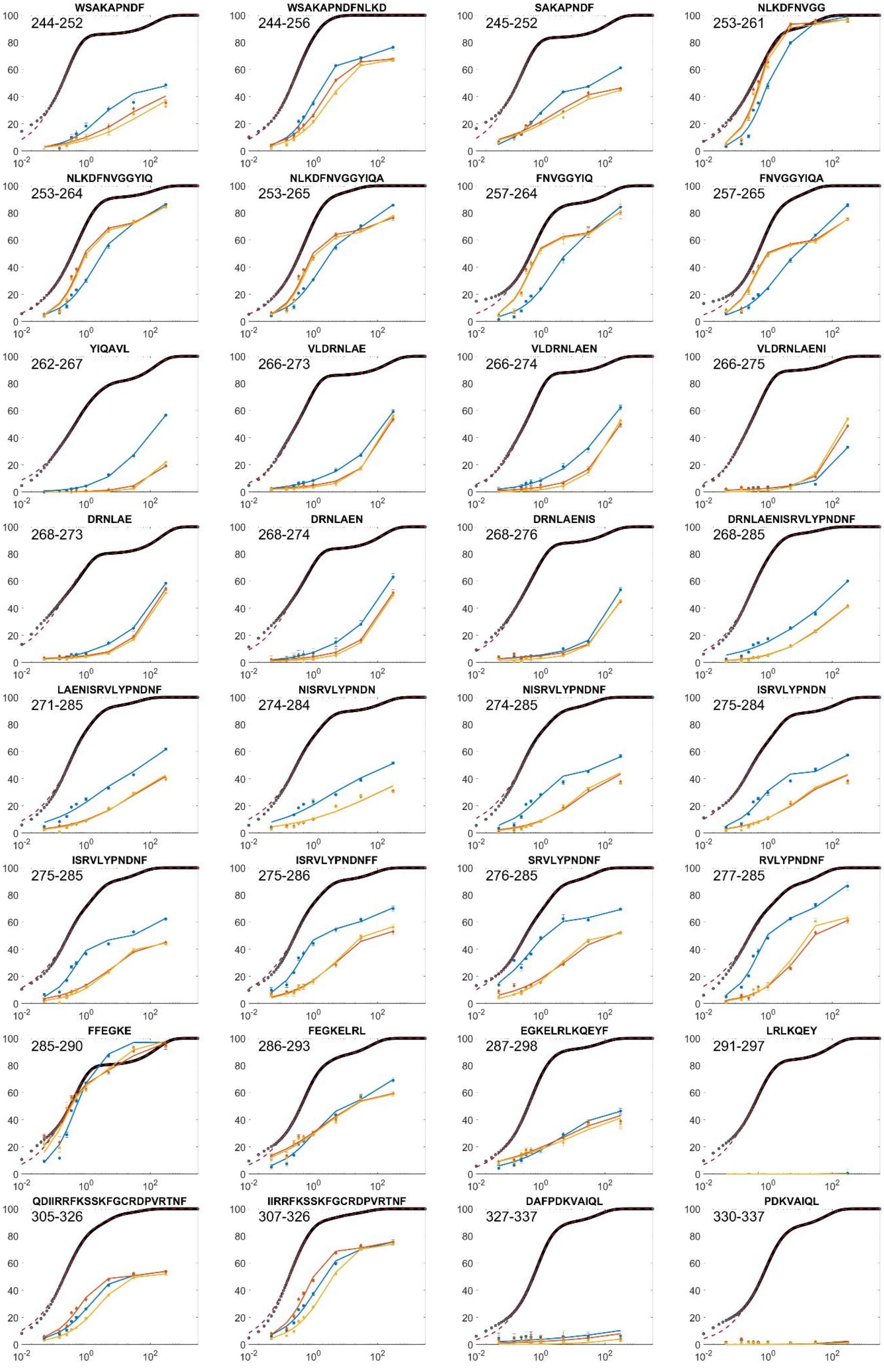
WSAKAPNDF
244-252
WSAKAPNDFNLKD
244-256
SAKAPNDF
245-252
NLKDFNVGG
253-261
NLKDFNVGGYIQ
253-264
NLKDFNVGGYIQA
253-265
FNVGGYIQ
257-264
FNVGGYIQA
257-265
YIQAVL
262-267
VLDRNLAE
266-273
VLDRNLAEN
266-274
VLDRNLAENI
266-275
DRNLAE
268-273
DRNLAEN
268-274
DRNLAENIS
268-276
DRNLAENISRVLYPNDNF
268-285
LAENISRVLYPNDNF
271-285
NISRVLYPNDN
274-284
NISRVLYPNDNF
274-285
ISRVLYPNDN
275-284
ISRVLYPNDNF
275-285
ISRVLYPNDNFF
275-286
SRVLYPNDNF
276-285
RVLYPNDNF
277-285
FFEGKE
285-290
FEGKELRL
286-293
EGKELRLKQEYF
287-298
LRLKQEY
291-297
QDIIRRFKSSKFGCRDPVRTNF
305-326
IIRRFKSSKFGCRDPVRTNF
307-326
DAFPDKVAIQL
327-337
PDKVAIQL
330-337

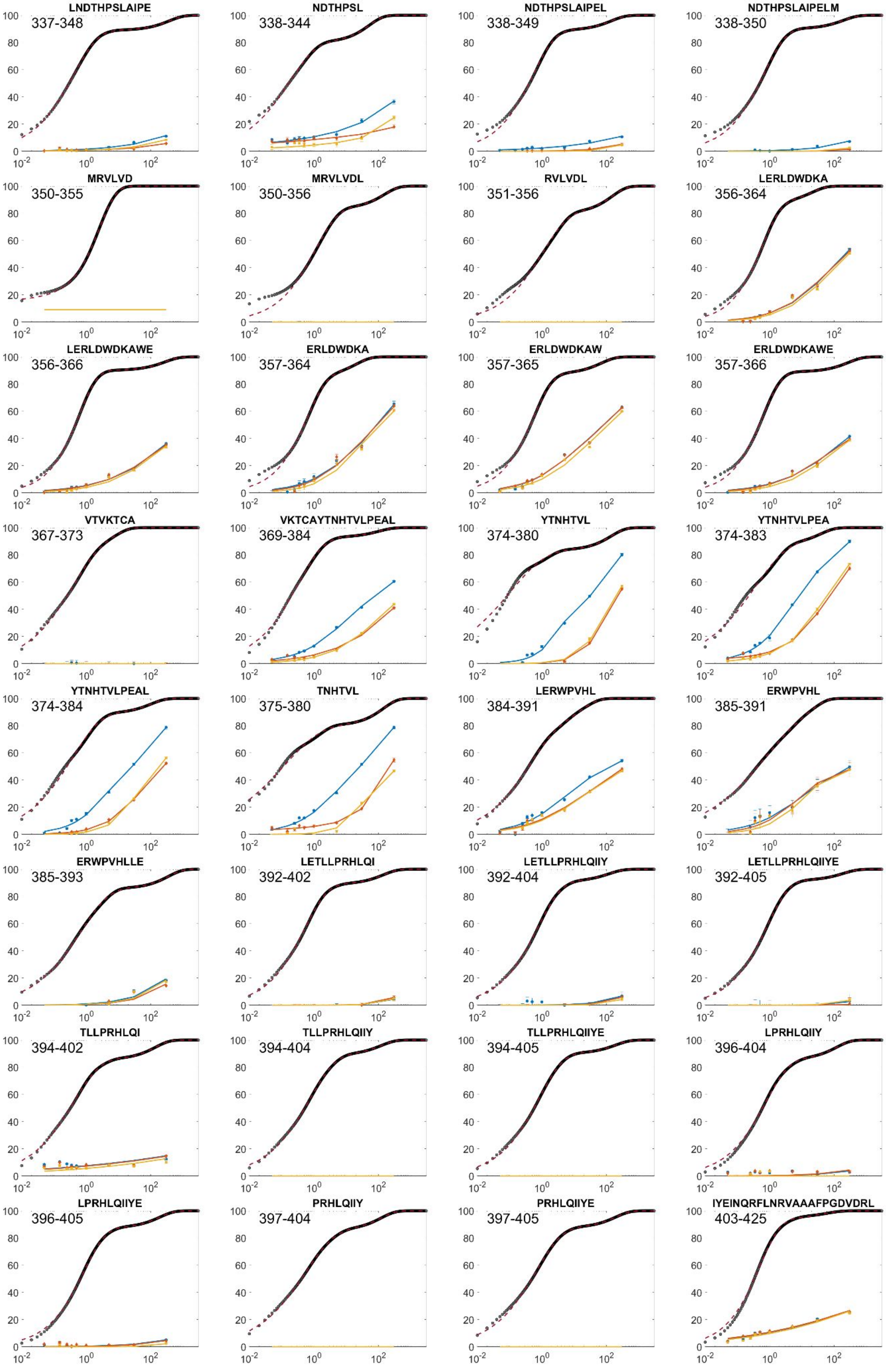
LNDTHPSLAIPE
337-348
NDTHPSL
338-344
NDTHPSLAIPEL
338-349
NDTHPSLAIPELM
338-350
MRVLVD
350-355
MRVLVDL
350-356
RVLVDL
351-356
LERLDWDKA
356-364
LERLDWDKAWE
356-366
ERLDWDKA
357-364
ERLDWDKAW
357-365
ERLDWDKAWE
357-366
VTVKTCA
367-373
VKTCAYTNHTVLPEAL
369-384
YTNHTVL
374-380
YTNHTVLPEA
374-383
YTNHTVLPEAL
374-384
TNHTVL
375-380
LERWPVHL
384-391
ERWPVHL
385-391
ERWPVHLLE
385-393
LETLLPRHLQI
392-402
LETLLPRHLQIIY
392-404
LETLLPRHLQIIYE
392-405
TLLPRHLQI
394-402
TLLPRHLQIIY
394-404
TLLPRHLQIIYE
394-405
LPRHLQIIY
396-404
LPRHLQIIYE
396-405
PRHLQIIY
397-404
PRHLQIIYE
397-405
IYEINQRFLNRVAAAFPGDVDRL
403-425

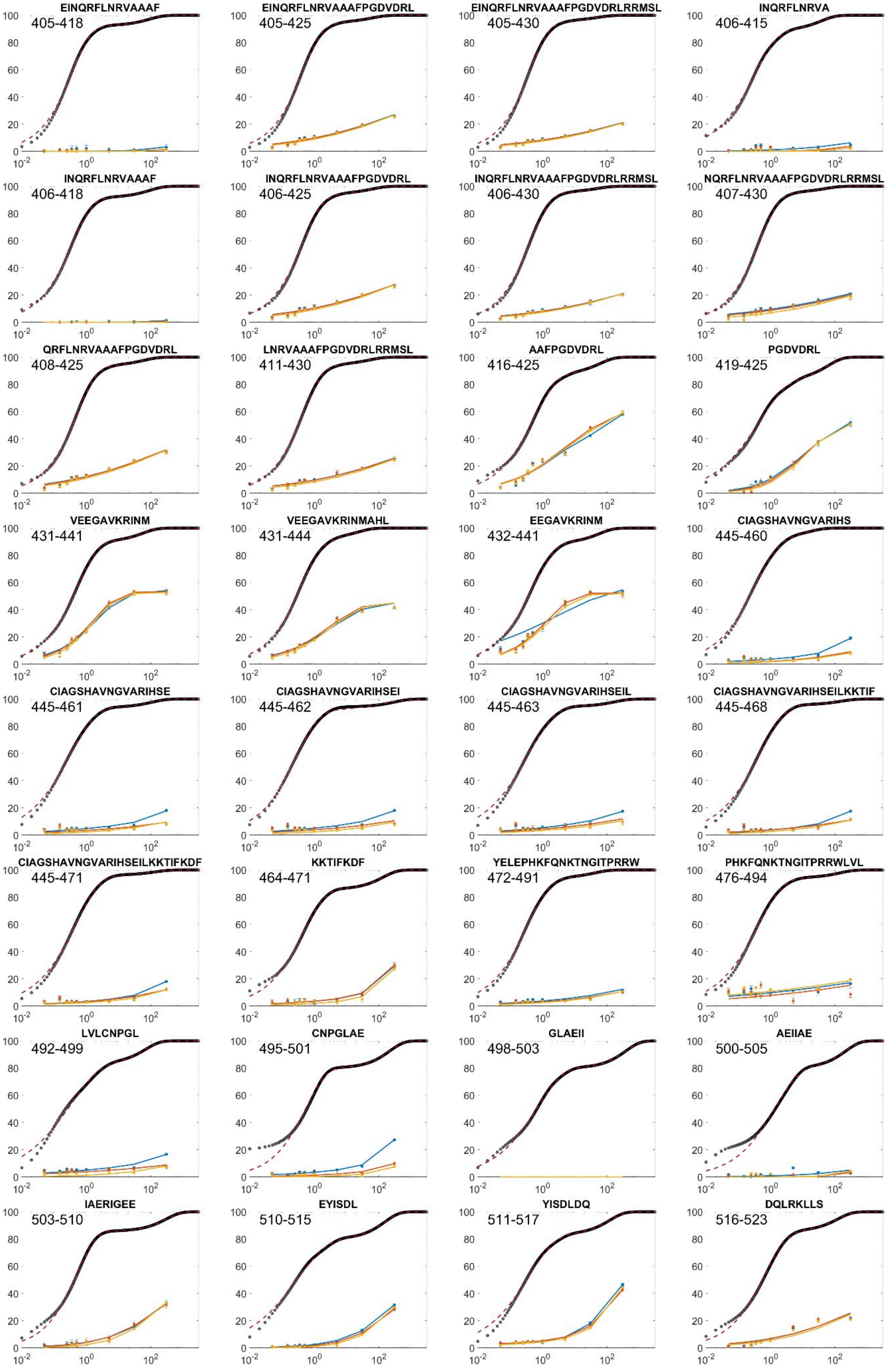
EINQRFLNRVAAAF
405-418
EINQRFLNRVAAAFPGDVDRL
405-425
EINQRFLNRVAAAFPGDVDRLRRMSL
405-430
INQRFLNRVA
406-415
INQRFLNRVAAAF
406-418
INQRFLNRVAAAFPGDVDRL
406-425
INQRFLNRVAAAFPGDVDRLRRMSL
406-430
NQRFLNRVAAAFPGDVDRLRRMSL
407-430
QRFLNRVAAAFPGDVDRL
408-425
LNRVAAAFPGDVDRLRRMSL
411-430
AAFPGDVDRL
416-425
PGDVDRL
419-425
VEEGAVKRINM
431-441
VEEGAVKRINMAHL
431-444
EEGAVKRINM
432-441
CIAGSHAVNGVARIHS
445-460
CIAGSHAVNGVARIHSE
445-461
CIAGSHAVNGVARIHSEI
445-462
CIAGSHAVNGVARIHSEIL
445-463
CIAGSHAVNGVARIHSEILKKTIF
445-468
CIAGSHAVNGVARIHSEILKKTIFKDF
445-471
KKTIFKDF
464-471
YELEPHKFQNKTNGITPRRW
472-491
PHKFQNKTNGITPRRWLVL
476-494
LVLCNPGL
492-499
CNPGLAE
495-501
GLAEII
498-503
AEIIAE
500-505
IAERIGEE
503-510
EYISDL
510-515
YISDLDQ
511-517
DQLRKLLS
516-523

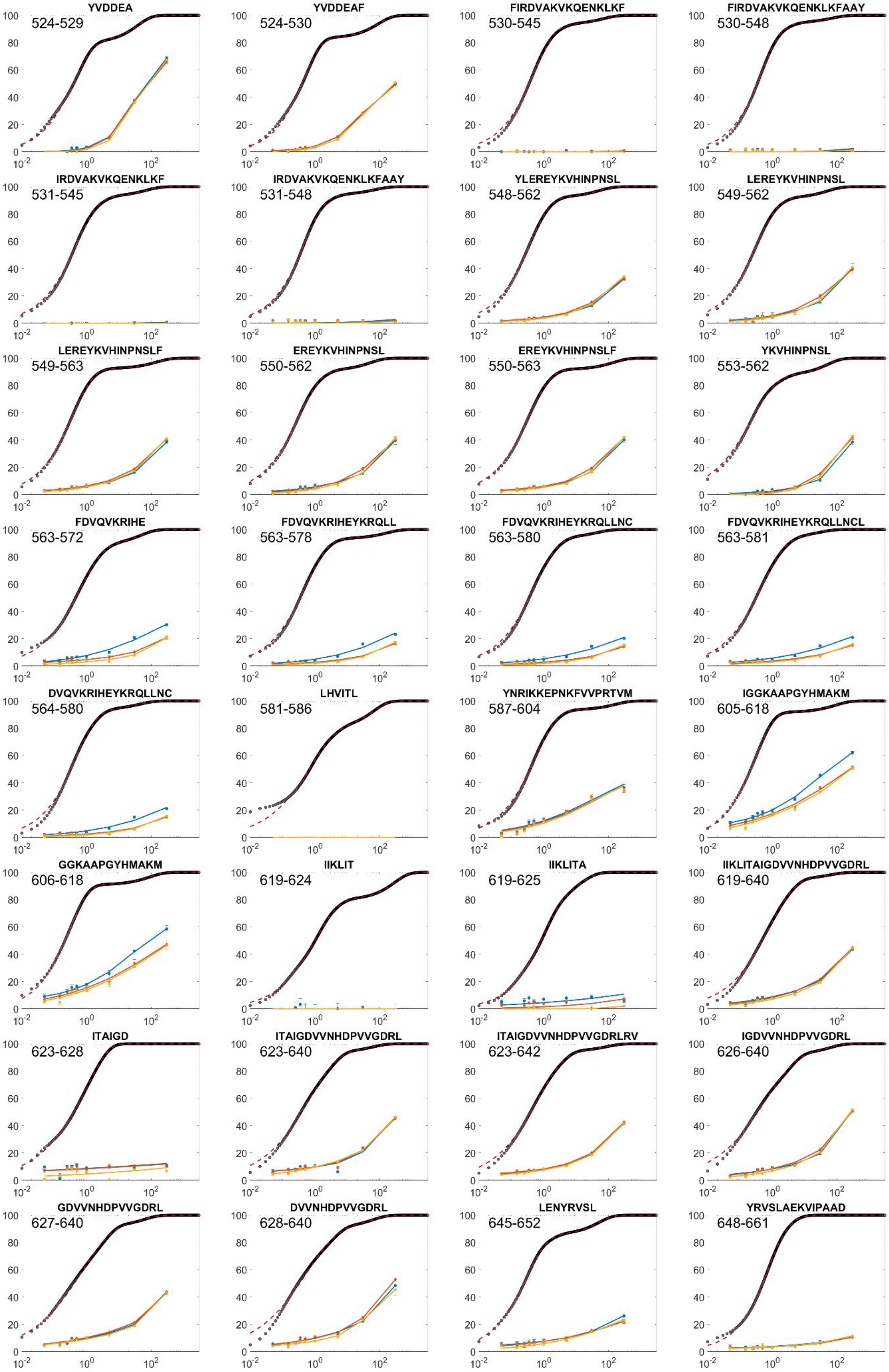
YVDDEA
524-529
YVDDEAF
524-530
FIRDVAKVKQENKLKF
530-545
FIRDVAKVKQENKLKFAAY
530-548
IRDVAKVKQENKLKF
531-545
IRDVAKVKQENKLKFAAY
531-548
YLEREYKVHINPNSL
548-562
LEREYKVHINPNSL
549-562
LEREYKVHINPNSLF
549-563
EREYKVHINPNSL
550-562
EREYKVHINPNSLF
550-563
YKVHINPNSL
553-562
FDVQVKRIHE
563-572
FDVQVKRIHEYKRQLL
563-578
FDVQVKRIHEYKRQLLNC
563-580
FDVQVKRIHEYKRQLLNCL
563-581
DVQVKRIHEYKRQLLNC
564-580
LHVITL
581-586
YNRIKKEPNKFVVPRTVM
587-604
IGGKAAPGYHMAKM
605-618
GGKAAPGYHMAKM
606-618
IIKLIT
619-624
IIKLITA
619-625
IIKLITAIGDVVNHDPVVGDRL
619-640
ITAIGD
623-628
ITAIGDVVNHDPVVGDRL
623-640
ITAIGDVVNHDPVVGDRLRV
623-642
IGDVVNHDPVVGDRL
626-640
GDVVNHDPVVGDRL
627-640
DVVNHDPVVGDRL
628-640
LENYRVSL
645-652
YRVSLAEKVIPAAD
648-661

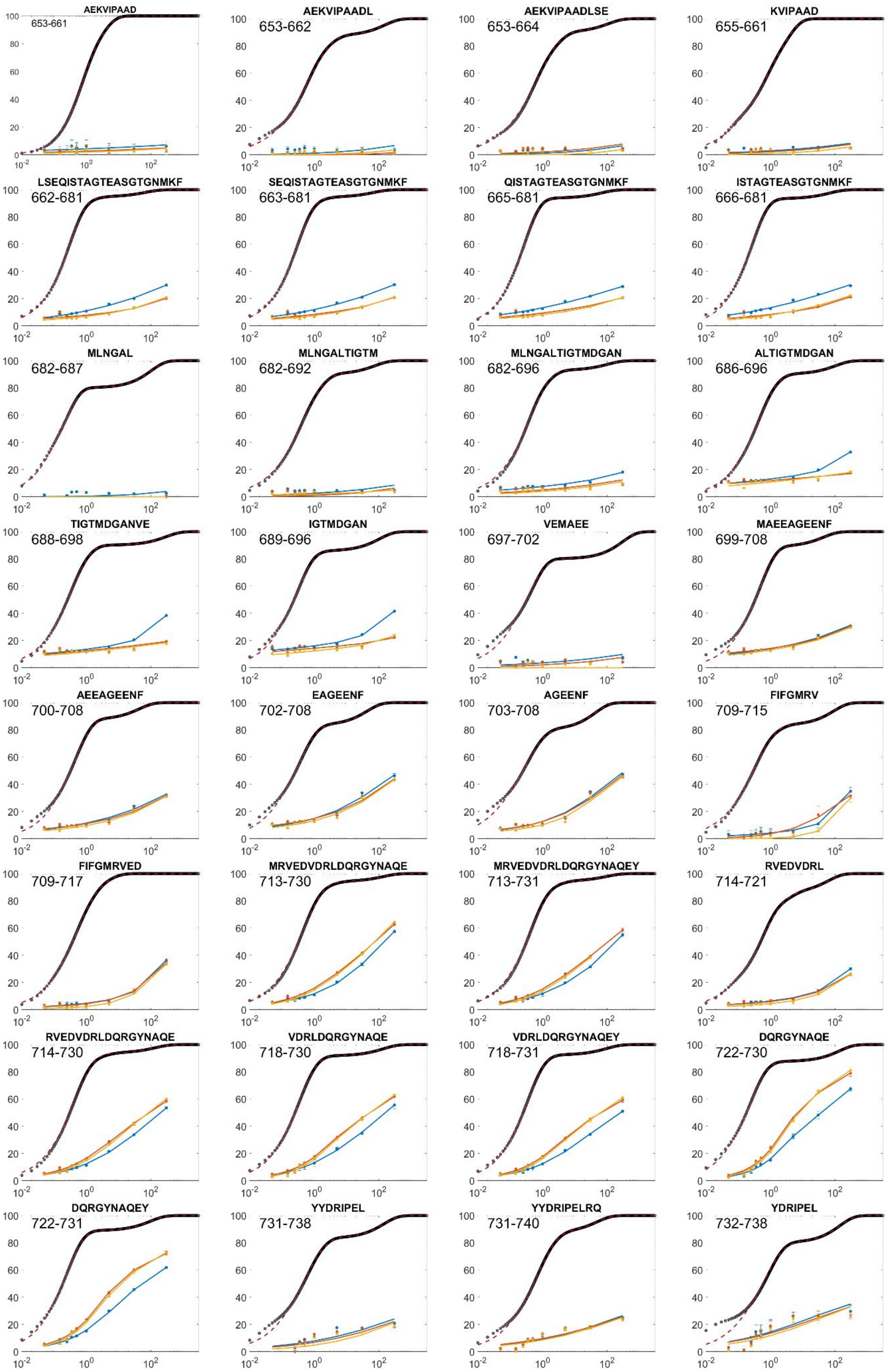
AEKVIPAAD
653-661
AEKVIPAADL
653-662
AEKVIPAADLSE
653-664
KVIPAAD
655-661
LSEQISTAGTEASGTGNMKF
662-681
SEQISTAGTEASGTGNMKF
663-681
QISTAGTEASGTGNMKF
665-681
ISTAGTEASGTGNMKF
666-681
MLNGAL
682-687
MLNGALTIGTM
682-692
MLNGALTIGTMDGAN
682-696
ALTIGTMDGAN
686-696
TIGTMDGANVE
688-698
IGTMDGAN
689-696
VEMAEE
697-702
MAEEAGEENF
699-708
AEEAGEENF
700-708
EAGEENF
702-708
AGEENF
703-708
FIFGMRV
709-715
FIFGMRVED
709-717
MRVEDVDRLDQRGYNAQE
713-730
MRVEDVDRLDQRGYNAQEY
713-731
RVEDVDRL
714-721
RVEDVDRLDQRGYNAQE
714-730
VDRLDQRGYNAQE
718-730
VDRLDQRGYNAQEY
718-731
DQRGYNAQE
722-730
DQRGYNAQEY
722-731
YYDRIPEL
731-738
YYDRIPELRQ
731-740
YDRIPEL
732-738

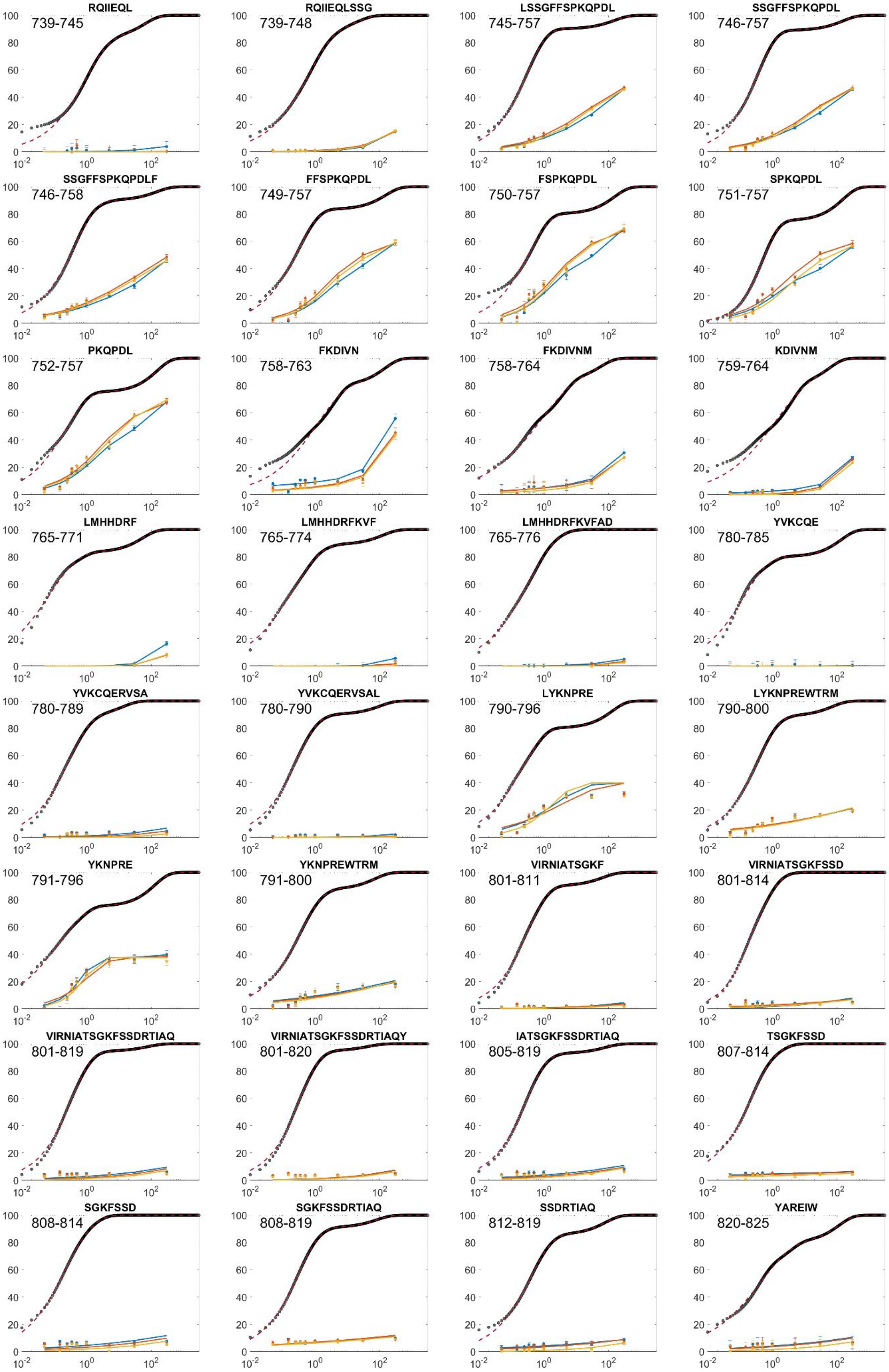
RQIIEQL
739-745
RQIIEQLSSG
739-748
LSSGFFSPKQPDL
745-757
SSGFFSPKQPDL
746-757
SSGFFSPKQPDLF
746-758
FFSPKQPDL
749-757
FSPKQPDL
750-757
SPKQPDL
751-757
PKQPDL
752-757
FKDIVN
758-763
FKDIVNM
758-764
KDIVNM
759-764
LMHHDRF
765-771
LMHHDRFKVF
765-774
LMHHDRFKVFAD
765-776
YVKCQE
780-785
YVKCQERVSA
780-789
YVKCQERVSAL
780-790
LYKNPRE
790-796
LYKNPREWTRM
790-800
YKNPRE
791-796
YKNPREWTRM
791-800
VIRNIATSGKF
801-811
VIRNIATSGKFSSD
801-814
VIRNIATSGKFSSDRTIAQ
801-819
VIRNIATSGKFSSDRTIAQY
801-820
IATSGKFSSDRTIAQ
805-819
TSGKFSSD
807-814
SGKFSSD
808-814
SGKFSSDRTIAQ
808-819
SSDRTIAQ
812-819
YAREIW
820-825

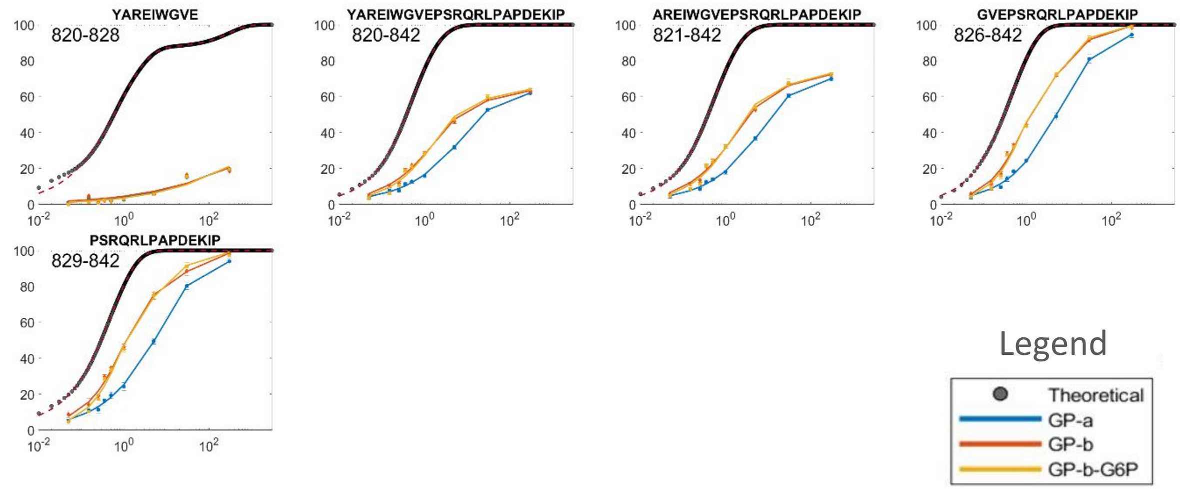
YAREIWGVE
820-828
YAREIWGVEPSRQRLPAPDEKIP
820-842
AREIWGVEPSRQRLPAPDEKIP
821-842
GVEPSRQRLPAPDEKIP
826-842
PSRQRLPAPDEKIP
829-842
Legend
Theoretical
GP-a
GP-b
GP-b-G6P

Table S1 Fitting parameters to multi-phase stretched exponential for hydrogen/deuterium-exchange mass spectrometry of GlyP in apo, activated and inactivated enzyme. Significantly different peptides at 3 time points are shown in the GlyPa vs GlyPb and GlyPb vs GlyP:G6P columns as red asterisk.

				Intrinsic fits						GlyPa'						GlyPb'						GlyPb-G6P'						Significantly different peptides	
Start	End	Sequence	Residues	k_{int1}	β_1	k_{int2}	N_1	β_2	N_2	k_{exp1}	β_1	k_{exp2}	N_1	β_2	N_2	k_{exp1}	β_1	k_{exp2}	N_1	β_2	N_2	k_{exp1}	β_1	k_{exp2}	N_1	β_2	N_2	GlyPa vs GlyPb	GlyPb vs GlyP:G6P
23	37	'NVTELKKNFNRHLHF'	23 - 37	3.0	0.7	0.1	12.4	0.7	1.6	4E-03	1.0	2E-14	6.2	0.5	7.8	3E-03	1.0	2E-14	6.2	0.5	7.8	2E-03	1.0	4E-14	6.2	0.5	7.8		
25	37	'TELKKNFNRHLHF'	25 - 37	3.8	0.7	0.1	10.3	0.7	1.7	3E-04	1.0	9E-14	5.1	0.5	6.9	1E-04	1.0	2E-14	5.1	0.5	6.9	8E-05	1.0	2E-14	5.1	0.5	6.9		
25	39	'TELKKNFNRHLHFTL'	25 - 39	3.5	0.9	0.0	12.8	0.7	1.2	9E-04	1.0	2E-14	6.4	0.5	7.6	7E-04	1.0	2E-14	6.4	0.5	7.6	5E-04	1.0	1E-13	6.4	0.5	7.6	*	*
27	37	'LKKNFNRHLHF'	27 - 37	4.4	0.9	0.1	8.9	0.8	1.1	8E-05	1.0	3E-13	4.5	0.5	5.5	6E-05	1.0	2E-14	4.5	0.5	5.5	1E-04	1.0	4E-14	4.5	0.7	5.5		
27	39	'LKKNFNRHLHFTL'	27 - 39	4.2	1.0	0.0	11.0	0.8	1.0	1E-04	1.0	9E-13	5.5	0.5	6.5	1E-04	1.0	2E-14	5.5	0.5	6.5	6E-05	1.0	2E-14	5.5	0.5	6.5		
38	47	'TLVKDRNVAT'	38 - 47	0.0	0.7	1.5	1.0	1.0	8.0	3E-14	0.5	5E-03	5.0	1.0	4.0	9E-04	0.5	2E-01	5.0	1.0	4.0	6E-14	0.5	6E-02	5.0	1.0	4.0	*	*
38	50	'TLVKDRNVATPRD'	38 - 50	1.9	0.9	0.4	8.6	0.8	2.4	3E-02	1.0	6E-14	4.3	0.5	6.7	3E-01	1.0	1E-03	4.3	0.5	6.7	7E-02	0.5	2E-14	4.8	0.5	6.2	*	
38	51	'TLVKDRNVATPRDY'	38 - 51	0.0	0.7	1.7	1.1	0.9	10.9	2E-14	0.5	1E-02	6.5	1.0	5.5	5E-04	0.5	2E-01	6.5	1.0	5.5	4E-14	0.5	6E-02	6.5	1.0	5.5	*	*
38	52	'TLVKDRNVATPRDYY'	38 - 52	1.6	0.9	0.0	11.8	0.7	1.2	5E-03	1.0	4E-14	5.9	0.5	7.1	7E-02	1.0	2E-05	5.9	0.5	7.1	2E-02	1.0	2E-14	5.9	0.5	7.1	*	*
38	53	'TLVKDRNVATPRDYYF'	38 - 53	1.6	0.9	0.0	12.9	0.7	1.1	2E-03	1.0	2E-14	6.4	0.5	7.6	2E-02	1.0	3E-14	6.4	0.5	7.6	5E-03	1.0	2E-14	6.4	0.5	7.6	*	*
40	52	'VKDRNVATPRDYY'	40 - 52	0.0	0.8	2.0	1.0	1.0	10.0	2E-14	0.5	2E-02	6.0	1.0	5.0	7E-04	0.5	2E-01	6.0	1.0	5.0	4E-14	0.5	5E-02	6.0	1.0	5.0	*	*
51	67	'YYFALAHTVRDHLVGRW'	51 - 67	2.1	0.8	0.0	14.6	0.7	1.4	4E-04	1.0	3E-13	7.3	0.5	8.7	4E-03	1.0	2E-14	7.3	0.5	8.7	3E-03	1.0	4E-14	7.3	0.5	8.7	*	
52	67	'YFALAHTVRDHLVGRW'	52 - 67	2.1	0.8	0.0	13.6	0.7	1.4	5E-04	1.0	3E-14	6.8	0.5	8.2	9E-03	1.0	2E-14	6.8	0.5	8.2	9E-03	1.0	2E-14	6.8	0.5	8.2	*	
53	61	'FALAHTVRD'	53 - 61	5.9	0.7	1.1	1.8	1.0	6.2	4E-03	1.0	2E-14	0.9	0.5	7.1	3E-03	1.0	2E-14	0.9	0.5	7.1	3E-03	1.0	2E-14	0.9	0.5	7.1		
53	63	'FALAHTVRDHL'	53 - 63	2.5	0.9	0.1	8.8	0.7	1.2	4E-05	1.0	2E-14	4.4	0.5	5.6	5E-05	1.0	2E-14	4.4	0.5	5.6	4E-05	1.0	4E-14	4.4	0.6	5.6		
53	65	'FALAHTVRDHLVG'	53 - 65	2.0	0.9	0.0	10.9	0.6	1.1	3E-04	1.0	2E-14	5.4	0.5	6.6	2E-03	1.0	2E-14	5.4	0.5	6.6	1E-03	1.0	2E-14	5.4	0.5	6.6	*	
53	67	'FALAHTVRDHLVGRW'	53 - 67	2.2	0.8	0.0	12.6	0.7	1.4	4E-04	1.0	2E-14	6.3	0.5	7.7	4E-03	1.0	2E-14	6.3	0.5	7.7	3E-03	1.0	2E-14	6.3	0.5	7.7	*	
54	61	'ALAHTVRD'	54 - 61	7.8	0.8	1.0	1.6	1.0	5.4	9E-09	1.0	9E-13	0.8	1.0	6.2	5E-04	0.7	4E-07	0.8	0.5	6.2	4E-04	1.0	2E-12	0.8	0.5	6.2	*	
54	67	'ALAHTVRDHLVGRW'	54 - 67	2.1	0.8	0.0	11.7	0.6	1.3	6E-04	1.0	5E-13	5.8	0.5	7.2	1E-02	1.0	2E-14	5.8	0.5	7.2	8E-03	1.0	2E-14	5.8	0.5	7.2	*	
56	67	'AHTVRDHLVGRW'	56 - 67	2.8	0.7	0.0	9.5	0.6	1.5	5E-04	1.0	3E-13	4.8	0.5	6.2	5E-03	1.0	4E-14	4.8	0.5	6.2	5E-03	1.0	2E-14	4.8	0.5	6.2	*	
57	67	'HTVRDHLVGRW'	57 - 67	2.4	0.8	0.0	8.6	0.7	1.4	5E-04	1.0	2E-14	4.3	0.5	5.7	4E-03	1.0	2E-14	4.3	0.5	5.7	3E-03	1.0	2E-14	4.3	0.5	5.7	*	
68	74	'IRTQQHY'	68 - 74	3.9	1.0	0.1	5.0	0.8	1.0	2E-04	1.0	3E-14	2.5	0.5	3.5	7E-04	1.0	2E-14	2.5	0.5	3.5	1E-04	1.0	6E-07	2.5	0.5	3.5		
68	84	'IRTQQHYYEKDPKRIYY'	68 - 84	2.3	1.0	0.0	14.0	0.7	1.0	3E-03	1.0	4E-14	7.0	0.5	8.0	4E-03	1.0	3E-14	7.0	0.5	8.0	2E-03	1.0	2E-09	7.0	0.6	8.0		

				Intrinsic fits						GlyPa'						GlyPb'						GlyPb-G6P'						Significantly different peptides	
Start	End	Sequence	Residues	k_{int1}	β_1	k_{int2}	N_1	β_2	N_2	k_{exp1}	β_1	k_{exp2}	N_1	β_2	N_2	k_{exp1}	β_1	k_{exp2}	N_1	β_2	N_2	k_{exp1}	β_1	k_{exp2}	N_1	β_2	N_2	GlyPa vs GlyPb	GlyPb vs GlyP:G6P
68	87	'IRTQQHYYEKDPKRIYYLSL'	68 - 87	2.1	0.9	0.0	16.9	0.8	1.1	5E-03	1.0	5E-11	16.3	0.6	1.7	1E-02	0.8	3E-07	11.0	0.5	7.0	1E-02	1.0	8E-05	8.5	0.9	9.5		
75	84	'YEKDPKRIYY'	75 - 84	0.0	0.8	1.5	1.0	1.0	7.0	2E-13	0.7	9E-03	4.5	1.0	3.5	2E-14	0.7	1E-02	4.5	1.0	3.5	4E-14	1.0	1E-02	4.5	1.0	3.5		
79	84	'PKRIYY'	79 - 84	0.0	0.8	1.1	1.0	1.0	4.0	5E-13	0.5	3E-03	2.0	1.0	2.0	4E-14	0.5	3E-03	2.0	1.0	2.0	7E-12	0.7	2E-03	2.0	1.0	2.0		
88	98	'EFYMGRTLQNT'	88 - 98	0.0	0.8	2.2	1.0	1.0	9.0	1E-03	0.5	6E-04	5.5	1.0	4.5	4E-07	0.8	1E-03	5.5	1.0	4.5	2E-14	1.0	1E-03	5.5	1.0	4.5		
88	99	'EFYMGRTLQNTM'	88 - 99	0.0	0.8	2.3	1.0	1.0	10.0	8E-06	1.0	3E-03	6.0	1.0	5.0	3E-04	1.0	8E-04	6.0	1.0	5.0	4E-04	1.0	7E-04	6.0	1.0	5.0		
88	101	'EFYMGRTLQNTMVN'	88 - 101	0.0	0.7	2.1	1.0	1.0	12.0	9E-04	0.5	3E-04	7.0	1.0	6.0	2E-04	0.5	4E-04	7.0	1.0	6.0	3E-04	0.6	3E-04	7.0	1.0	6.0		*
89	98	'FYMGRTLQNT'	89 - 98	0.0	0.8	2.5	1.0	1.0	8.0	5E-04	0.5	7E-04	5.0	1.0	4.0	2E-14	0.5	8E-04	5.0	1.0	4.0	2E-14	0.5	6E-04	5.0	1.0	4.0		
89	99	'FYMGRTLQNTM'	89 - 99	0.0	0.8	2.6	1.0	1.0	9.0	1E-03	0.6	8E-04	5.5	1.0	4.5	3E-04	0.5	5E-04	5.5	1.0	4.5	4E-04	0.5	3E-04	5.5	1.0	4.5		
89	101	'FYMGRTLQNTMVN'	89 - 101	0.0	0.8	2.3	1.0	1.0	11.0	9E-05	0.5	1E-03	6.5	1.0	5.5	2E-05	0.5	5E-04	6.5	1.0	5.5	4E-04	0.6	2E-04	6.5	1.0	5.5		
90	98	'YMGRTLQNT'	90 - 98	0.0	0.8	2.7	1.0	1.0	7.0	5E-04	0.5	3E-03	4.5	1.0	3.5	4E-06	0.5	3E-03	4.5	1.0	3.5	5E-05	0.5	2E-03	4.5	1.0	3.5		
90	99	'YMGRTLQNTM'	90 - 99	0.0	0.8	2.8	1.0	1.0	8.0	5E-04	0.9	2E-03	5.0	1.0	4.0	6E-04	0.6	5E-04	4.9	1.0	4.1	2E-14	0.8	2E-03	5.0	0.5	4.0		
90	102	'YMGRTLQNTMVNL'	90 - 102	2.7	0.9	0.0	10.9	0.8	1.1	3E-04	1.0	4E-04	5.4	0.5	6.6	3E-04	1.0	1E-04	5.5	0.5	6.5	9E-04	1.0	6E-07	5.4	0.8	6.6		
102	109	'LALENACD'	102 - 109	1.2	1.0	6.3	4.8	0.8	2.2	1E-04	0.6	8E-02	4.4	0.5	2.6	2E-07	0.6	7E-03	3.1	0.8	3.9	2E-04	0.7	4E-03	3.2	0.9	3.8	*	
103	109	'ALENACD'	103 - 109	1.1	1.0	6.5	3.6	0.8	2.4	1E-03	1.0	1E-01	3.7	1.0	2.3	2E-09	1.0	1E-02	3.0	1.0	3.0	1E-08	1.0	5E-03	2.6	1.0	3.4	*	
103	110	'ALENACDE'	103 - 110	0.0	0.6	3.1	1.0	1.0	6.0	6E-05	0.6	6E-02	3.1	0.8	3.9	1E-03	0.5	1E-02	3.9	1.0	3.1	1E-03	0.5	3E-03	4.0	1.0	3.0	*	*
110	117	'EATYQLGL'	110 - 117	0.0	0.9	1.7	1.0	1.0	6.0	2E-14	0.6	4E-05	4.0	1.0	3.0	3E-07	0.5	3E-05	4.0	1.0	3.0	2E-06	0.7	6E-05	4.0	1.0	3.0		
111	117	'ATYQLGL'	111 - 117	0.0	0.9	1.7	1.0	1.0	5.0	2E-14	0.5	4E-05	3.5	1.0	2.5	6E-12	0.5	6E-05	3.5	1.0	2.5	3E-06	0.7	1E-04	3.5	1.0	2.5		
111	118	'ATYQLGLD'	111 - 118	0.3	0.9	1.6	1.6	0.8	5.4	2E-14	0.5	9E-04	4.3	1.0	2.7	3E-14	0.5	9E-04	4.3	1.0	2.7	8E-12	0.5	8E-04	4.3	1.0	2.7	*	
111	119	'ATYQLGLDM'	111 - 119	0.0	0.9	1.5	1.0	1.0	7.0	2E-14	0.5	2E-03	4.5	1.0	3.5	3E-14	0.5	9E-04	4.5	1.0	3.5	4E-14	0.5	5E-04	4.5	1.0	3.5		
113	118	'YQLGLD'	113 - 118	0.2	0.9	1.4	1.3	0.9	3.7	3E-12	0.6	1E-03	3.1	1.0	1.9	1E-10	0.6	2E-03	3.1	1.0	1.9	4E-14	0.6	2E-03	3.1	1.0	1.9		
113	119	'YQLGLDM'	113 - 119	0.0	0.9	1.4	1.0	1.0	5.0	2E-14	0.6	2E-03	3.5	1.0	2.5	4E-14	0.6	2E-03	3.5	1.0	2.5	2E-14	0.7	1E-03	3.5	1.0	2.5		
123	139	'EEIEEDAGLGNGGLGRL'	123 - 139	0.0	0.8	1.6	1.2	0.9	14.8	2E-03	0.5	2E-01	8.6	1.0	7.4	9E-14	0.5	2E-02	8.6	1.0	7.4	3E-14	0.5	2E-02	8.6	1.0	7.4	*	
125	139	'IEEDAGLGNGGLGRL'	125 - 139	1.8	1.0	0.0	13.0	0.8	1.0	2E-01	1.0	1E-03	6.5	0.5	7.5	2E-02	1.0	2E-14	6.5	0.5	7.5	2E-02	1.0	1E-12	6.5	0.5	7.5	*	
125	142	'IEEDAGLGNGGLGRLAAC'	125 - 142	0.0	0.8	1.8	1.0	1.0	16.0	7E-04	0.5	9E-02	9.0	1.0	8.0	3E-14	0.5	7E-03	9.0	1.0	8.0	2E-14	0.5	6E-03	9.0	1.0	8.0	*	
128	139	'DAGLGNGGLGRL'	128 - 139	2.0	1.0	0.0	10.0	0.8	1.0	2E-01	1.0	1E-03	5.0	0.5	6.0	2E-02	1.0	2E-14	5.0	0.5	6.0	2E-02	1.0	2E-14	5.0	0.5	6.0	*	
128	141	'DAGLGNGGLGRLAA'	128 - 141	1.8	1.0	0.0	12.0	0.8	1.0	2E-01	1.0	5E-04	6.0	0.5	7.0	2E-02	1.0	2E-14	6.0	0.5	7.0	2E-02	1.0	2E-14	6.0	0.5	7.0	*	
144	149	'LDSMAT'	144 - 149	0.0	0.9	3.0	1.0	1.0	4.0	3E-14	1.0	3E-08	3.0	1.0	2.0	3E-14	1.0	3E-09	3.0	1.0	2.0	2E-14	1.0	4E-12	3.0	1.0	2.0		
144	152	'LDSMATLGL'	144 - 152	0.0	0.9	2.1	1.0	1.0	7.0	6E-07	0.7	2E-05	4.5	1.0	3.5	1E-08	0.8	6E-07	4.5	1.0	3.5	2E-14	1.0	2E-10	4.5	1.0	3.5		

				Intrinsic fits						GlyPa'						GlyPb'						GlyPb-G6P'						Significantly different peptides	
Start	End	Sequence	Residues	k_{int1}	β_1	k_{int2}	N_1	β_2	N_2	k_{exp1}	β_1	k_{exp2}	N_1	β_2	N_2	k_{exp1}	β_1	k_{exp2}	N_1	β_2	N_2	k_{exp1}	β_1	k_{exp2}	N_1	β_2	N_2	GlyPa vs GlyPb	GlyPb vs GlyP:G6P
147	152	'MATLGL'	147 - 152	0.0	0.9	1.6	1.0	1.0	4.0	3E-14	1.0	2E-10	3.0	1.0	2.0	2E-14	1.0	6E-11	3.0	1.0	2.0	2E-14	1.0	6E-11	3.0	1.0	2.0		
147	154	'MATLGLAA'	147 - 154	1.4	1.0	0.0	6.0	0.9	1.0	2E-04	1.0	2E-14	3.0	0.5	4.0	3E-04	1.0	2E-04	3.0	0.5	4.0	1E-04	1.0	3E-04	3.0	0.6	4.0		*
148	154	'ATLGLAA'	148 - 154	0.0	1.0	1.3	1.0	1.0	5.0	2E-04	1.0	3E-04	3.5	1.0	2.5	5E-04	1.0	4E-04	3.5	1.0	2.5	5E-04	1.0	1E-04	3.5	1.0	2.5		
153	158	'AAYGYG'	153 - 158	0.0	1.0	1.9	1.0	1.0	4.0	3E-13	1.0	4E-09	3.0	1.0	2.0	4E-14	1.0	3E-10	3.0	1.0	2.0	3E-14	1.0	2E-10	3.0	1.0	2.0		
153	163	'AAYGYGIRYEF'	153 - 163	0.0	0.9	1.7	1.0	1.0	9.0	2E-14	1.0	4E-14	5.5	1.0	4.5	2E-14	1.0	9E-13	5.5	1.0	4.5	2E-14	1.0	8E-11	5.5	1.0	4.5		
155	163	'YGYGIRYEF'	155 - 163	0.0	0.9	1.7	1.0	1.0	7.0	3E-05	1.0	3E-04	4.5	1.0	3.5	2E-14	1.0	6E-10	4.5	1.0	3.5	2E-14	1.0	3E-11	4.5	1.0	3.5		
167	176	'NQKICGGWQM'	167 - 176	0.0	0.7	2.7	1.1	0.9	7.9	2E-03	0.5	1E+00	5.0	1.0	4.0	3E-14	0.5	2E+00	5.0	1.0	4.0	2E-14	0.5	1E+00	5.0	1.0	4.0		
176	181	'MEEADD'	176 - 181	0.2	1.0	2.0	1.1	0.9	3.9	2E-07	0.5	5E-02	0.9	0.5	4.1	1E-03	0.5	1E-02	3.1	1.0	1.9	2E-03	0.5	6E-03	3.1	1.0	1.9	*	
182	196	'WLRYGNPWEKARPEF'	182 - 196	0.0	0.7	1.6	1.0	1.0	11.0	3E-14	0.5	4E-02	6.5	1.0	5.5	2E-14	0.5	3E-02	6.5	1.0	5.5	2E-14	0.5	9E-03	6.5	1.0	5.5		
182	198	'WLRYGNPWEKARPEFTL'	182 - 198	1.6	0.9	0.0	12.9	0.8	1.1	3E-02	1.0	3E-14	6.5	0.5	7.5	1E-02	1.0	2E-14	6.5	0.5	7.5	5E-03	1.0	3E-14	6.5	0.5	7.5		
182	202	'WLRYGNPWEKARPEFTLPVHF'	182 - 202	1.7	0.6	0.2	14.1	0.8	2.9	3E-02	1.0	2E-13	7.0	0.5	10.0	8E-03	1.0	2E-14	7.0	0.5	10.0	2E-03	1.0	4E-14	7.0	0.5	10.0		*
183	202	'LRYGNPWEKARPEFTLPVHF'	183 - 202	1.8	0.6	0.1	13.5	0.8	2.5	4E-02	1.0	1E-13	6.7	0.5	9.3	3E-02	1.0	2E-14	6.7	0.5	9.3	1E-02	1.0	2E-14	6.7	0.5	9.3		
203	215	'YGRVEHTSQGAKW'	203 - 215	3.2	0.9	0.0	10.9	0.8	1.1	6E-01	0.5	3E-03	5.7	0.5	6.3	5E-01	0.5	3E-03	6.3	0.5	5.7	4E-01	0.5	2E-03	6.2	0.5	5.8		
203	218	'YGRVEHTSQGAKWVDT'	203 - 218	2.4	1.0	0.0	14.0	0.7	1.0	3E-01	1.0	2E-03	7.0	0.5	8.0	2E-01	0.8	2E-03	7.0	0.5	8.0	1E-01	1.0	2E-03	7.4	0.5	7.6		
203	219	'YGRVEHTSQGAKWVDTQ'	203 - 219	0.0	0.7	2.4	1.2	0.8	14.8	2E-03	0.5	2E-01	8.6	0.9	7.4	2E-03	0.5	2E-01	8.6	0.7	7.4	1E-03	0.5	1E-01	8.6	0.6	7.4		
203	221	'YGRVEHTSQGAKWVDTQVV'	203 - 221	0.0	0.7	2.2	1.0	1.0	17.0	1E-03	0.5	1E-01	9.5	1.0	8.5	2E-03	0.5	1E-01	9.5	1.0	8.5	1E-03	0.5	7E-02	8.8	0.9	9.2		
219	224	'QVVLAM'	219 - 224	0.0	0.9	0.6	1.0	1.0	4.0	1E-11	0.5	3E-05	3.0	1.0	2.0	2E-14	0.7	1E-04	3.0	1.0	2.0	6E-07	0.8	2E-04	3.0	1.0	2.0		
219	235	'QVVLAMPYDTPVPGYRN'	219 - 235	0.0	0.7	1.0	1.2	0.9	11.8	4E-14	0.5	9E-05	7.1	1.0	5.9	2E-14	0.5	1E-04	7.1	1.0	5.9	3E-14	0.5	8E-05	7.1	1.0	5.9		
219	240	'QVVLAMPYDTPVPGYRNNVVNT'	219 - 240	0.0	0.6	1.2	1.1	0.9	16.9	2E-14	0.5	2E-04	9.5	1.0	8.5	4E-14	0.5	4E-04	9.5	1.0	8.5	4E-14	0.5	2E-04	9.5	1.0	8.5		
220	240	'VVLAMPYDTPVPGYRNNVVNT'	220 - 240	0.0	0.6	1.3	1.1	0.9	15.9	2E-14	0.5	2E-04	9.0	1.0	8.0	2E-14	0.5	4E-04	9.0	1.0	8.0	3E-14	0.5	3E-04	9.0	1.0	8.0		
222	235	'LAMPYDTPVPGYRN'	222 - 235	0.0	0.8	1.4	1.2	0.8	8.8	3E-14	0.5	1E-04	5.6	1.0	4.4	4E-14	0.5	4E-05	5.6	1.0	4.4	2E-14	0.5	6E-05	5.6	1.0	4.4		
222	240	'LAMPYDTPVPGYRNNVVNT'	222 - 240	0.0	0.6	1.6	1.1	0.9	13.9	2E-14	0.5	2E-04	8.1	1.0	6.9	3E-14	0.5	4E-04	8.1	1.0	6.9	1E-12	0.5	3E-04	8.1	1.0	6.9		
223	235	'AMPYDTPVPGYRN'	223 - 235	0.0	0.8	1.4	1.2	0.8	7.8	4E-14	0.5	3E-04	5.1	1.0	3.9	2E-14	0.5	2E-04	5.1	1.0	3.9	3E-14	0.5	2E-04	5.1	1.0	3.9		
223	240	'AMPYDTPVPGYRNNVVNT'	223 - 240	1.6	0.9	0.0	12.9	0.6	1.1	3E-04	1.0	3E-14	6.4	0.5	7.6	5E-04	1.0	2E-13	6.4	0.5	7.6	3E-04	1.0	2E-14	6.4	0.5	7.6		
225	240	'PYDTPVPGYRNNVVNT'	225 - 240	0.0	0.6	1.6	1.1	0.9	11.9	2E-14	0.5	3E-04	6.0	1.0	6.0	2E-14	0.5	6E-04	6.0	1.0	6.0	3E-14	0.5	4E-04	6.0	1.0	6.0		
244	252	'WSAKAPNDF'	244 - 252	0.0	0.9	3.7	1.0	1.0	6.0	3E-04	0.6	3E-01	4.0	1.0	3.0	3E-13	0.5	6E-02	4.0	1.0	3.0	2E-14	0.5	3E-02	4.0	1.0	3.0		
244	256	'WSAKAPNDFNLKD'	244 - 256	3.8	0.7	1.1	6.1	1.0	4.9	8E-01	0.6	7E-04	7.1	0.8	3.9	4E-01	0.5	5E-06	7.3	0.7	3.7	2E-01	0.6	3E-05	7.1	0.6	3.9	*	
245	252	'SAKAPNDF'	245 - 252	0.0	0.9	3.7	1.0	1.0	5.0	1E-03	0.7	1E+00	3.3	0.9	2.7	3E-04	0.5	6E-01	3.5	1.0	2.5	2E-04	0.5	4E-01	3.5	1.0	2.5	*	*

				Intrinsic fits						GlyPa'						GlyPb'						GlyPb-G6P'						Significantly different peptides	
Start	End	Sequence	Residues	k_{int1}	β_1	k_{int2}	N_1	β_2	N_2	k_{exp1}	β_1	k_{exp2}	N_1	β_2	N_2	k_{exp1}	β_1	k_{exp2}	N_1	β_2	N_2	k_{exp1}	β_1	k_{exp2}	N_1	β_2	N_2	GlyPa vs GlyPb	GlyPb vs GlyP:G6P
253	261	'NLKDFNVGG'	253 - 261	1.6	1.0	0.0	7.0	0.8	1.0	1E+00	1.0	6E-02	5.9	1.0	2.1	2E+00	1.0	3E-01	6.2	1.0	1.7	1E+00	0.7	5E-02	6.9	1.0	1.0	*	
253	264	'NLKDFNVGGYIQ'	253 - 264	0.0	0.8	1.6	1.0	1.0	10.0	3E-03	0.7	5E-01	4.1	0.5	6.9	2E-03	1.0	1E+00	3.7	0.6	7.3	3E-03	1.0	1E+00	4.0	0.5	7.0	*	
253	265	'NLKDFNVGGYIQA'	253 - 265	0.1	0.8	1.6	1.3	0.9	10.7	4E-03	0.7	6E-01	5.2	0.5	6.8	8E-04	1.0	2E+00	4.6	0.5	7.4	1E-03	1.0	2E+00	4.9	0.5	7.1	*	
257	264	'FNVGGYIQ'	257 - 264	0.0	0.7	1.6	1.0	1.0	6.0	4E-03	0.7	4E-01	3.2	0.6	3.8	2E-03	1.0	2E+00	2.7	0.8	4.3	2E-03	1.0	2E+00	2.7	1.0	4.3	*	
257	265	'FNVGGYIQA'	257 - 265	1.7	0.9	0.1	6.8	0.8	1.2	5E-01	0.5	6E-03	3.5	0.6	4.5	2E+00	0.7	2E-03	4.5	1.0	3.5	2E+00	0.9	2E-03	4.4	1.0	3.6	*	
262	267	'YIQAVL'	262 - 267	0.0	0.7	0.9	1.0	1.0	4.0	1E-03	0.6	3E-02	3.0	1.0	2.0	6E-06	0.8	2E-03	3.0	1.0	2.0	1E-04	0.9	2E-03	3.0	1.0	2.0	*	*
266	273	'VLDRNLAE'	266 - 273	0.0	0.7	1.5	1.0	1.0	6.0	1E-03	0.5	4E-02	4.0	1.0	3.0	1E-03	0.5	8E-03	4.0	1.0	3.0	2E-03	0.5	6E-03	4.0	1.0	3.0	*	
266	274	'VLDRNLAEN'	266 - 274	0.0	0.8	1.6	1.0	1.0	7.0	1E-03	0.5	5E-02	4.5	1.0	3.5	1E-03	0.5	6E-03	4.5	1.0	3.5	2E-03	0.6	4E-03	4.5	0.9	3.5	*	
266	275	'VLDRNLAENI'	266 - 275	0.0	0.7	1.8	1.0	1.0	8.0	9E-04	0.5	1E-03	5.0	1.0	4.0	2E-03	0.5	2E-03	5.0	1.0	4.0	2E-03	0.7	5E-03	5.0	0.9	4.0		
268	273	'DRNLAE'	268 - 273	0.0	0.8	2.1	1.0	1.0	4.0	1E-03	0.5	3E-02	3.0	1.0	2.0	1E-03	0.5	1E-02	3.0	1.0	2.0	1E-03	0.5	8E-03	3.0	1.0	2.0		
268	274	'DRNLAEN'	268 - 274	0.0	0.8	2.0	1.0	1.0	5.0	2E-03	0.5	3E-02	3.5	1.0	2.5	1E-03	0.5	7E-03	3.5	1.0	2.5	2E-03	0.5	4E-03	3.5	1.0	2.5		
268	276	'DRNLAENIS'	268 - 276	0.0	0.8	2.0	1.0	1.0	7.0	1E-03	0.5	7E-03	4.5	1.0	3.5	1E-03	0.5	5E-03	4.5	1.0	3.5	1E-03	0.5	3E-03	4.5	1.0	3.5		*
268	285	'DRNLAENISRVLYPNDNF'	268 - 285	0.0	0.7	2.0	1.1	0.9	14.9	9E-04	0.5	1E-01	8.6	1.0	7.4	9E-06	0.5	2E-02	8.6	1.0	7.4	1E-13	0.5	2E-02	8.6	1.0	7.4	*	
271	285	'LAENISRVLYPNDNF'	271 - 285	1.9	0.9	0.0	11.9	0.7	1.1	5E-01	1.0	1E-03	6.0	0.5	7.1	4E-02	1.0	2E-13	6.0	0.5	7.0	4E-02	1.0	3E-13	6.0	0.5	7.0	*	
274	284	'NISRVLYPNDN'	274 - 284	0.0	0.7	1.6	1.0	1.0	8.0	4E-04	0.5	4E-01	5.0	1.0	4.0	2E-14	0.5	4E-02	5.0	1.0	4.0	2E-14	0.5	4E-02	5.0	1.0	4.0	*	
274	285	'NISRVLYPNDNF'	274 - 285	0.0	0.6	1.8	1.0	1.0	9.0	8E-04	0.6	9E-01	5.5	1.0	4.5	3E-14	0.5	5E-02	5.5	1.0	4.5	2E-14	0.5	6E-02	5.5	1.0	4.5	*	
275	284	'ISRVLYPNDN'	275 - 284	0.0	0.6	1.7	1.0	1.0	7.0	9E-04	0.8	1E+00	4.5	1.0	3.5	2E-14	0.5	8E-02	4.5	1.0	3.5	3E-14	0.5	8E-02	4.5	1.0	3.5	*	
275	285	'ISRVLYPNDNF'	275 - 285	0.0	0.6	2.0	1.1	0.9	7.9	6E-04	1.0	2E+00	5.0	0.5	4.0	6E-05	0.5	1E-01	5.0	1.0	4.0	7E-12	0.6	1E-01	5.0	1.0	4.0	*	
275	286	'ISRVLYPNDNFF'	275 - 286	0.0	0.6	2.1	1.0	1.0	9.0	7E-04	0.8	2E+00	4.7	0.5	5.3	2E-04	0.5	2E-01	5.0	0.9	5.0	4E-07	0.5	1E-01	4.3	0.8	5.7	*	
276	285	'SRVLYPNDNF'	276 - 285	0.0	0.6	1.8	1.0	1.0	7.0	7E-04	0.5	2E+00	3.0	1.0	5.0	5E-05	0.5	2E-01	4.3	0.5	3.7	8E-05	0.5	2E-01	4.0	1.0	4.0	*	*
277	285	'RVLYPNDNF'	277 - 285	0.0	0.6	1.5	1.0	1.0	6.0	5E-03	1.0	2E+00	3.1	0.5	3.9	3E-07	0.6	9E-02	2.7	0.5	4.3	7E-07	0.7	1E-01	2.6	0.7	4.4	*	
285	290	'FFEGKE'	285 - 290	0.0	1.0	1.9	1.0	1.0	4.0	5E-03	1.0	2E+00	0.7	0.5	4.3	8E-02	0.7	5E+00	2.1	0.5	2.8	1E-01	1.0	5E+00	2.4	0.5	2.5	*	
286	293	'FEGKELRL'	286 - 293	0.0	0.8	1.6	1.1	0.9	5.9	9E-04	0.6	7E-01	3.6	0.5	3.4	3E-04	0.5	1E+00	3.7	0.5	3.3	3E-04	0.5	1E+00	3.8	0.5	3.2	*	
287	298	'EGKELRLKQEYF'	287 - 298	0.0	0.8	1.5	1.1	0.9	9.9	5E-05	0.5	2E-01	6.0	1.0	5.0	2E-14	0.5	3E-01	6.0	1.0	5.0	3E-14	0.5	2E-01	6.0	1.0	5.0		
291	297	'LRLKQEY'	291 - 297	0.0	0.9	1.8	1.0	1.0	5.0	1E-05	1.0	1E-05	3.5	1.0	2.5	2E-14	1.0	5E-10	3.5	1.0	2.5	2E-14	1.0	2E-10	3.5	1.0	2.5		
305	326	'QDIIRRFKSSKFGCRDPVRTNF'	305 - 326	0.4	0.8	3.4	6.5	0.4	13.5	3E-05	0.7	6E-01	10.2	0.5	9.8	3E-05	0.8	1E+00	10.2	0.5	9.8	2E-05	0.7	3E-01	10.2	0.6	9.8	*	*
307	326	'IIRRFKSSKFGCRDPVRTNF'	307 - 326	0.1	0.7	3.7	3.4	0.5	14.6	3E-04	0.7	7E-01	5.9	0.5	12.1	2E-04	0.8	1E+00	5.6	0.5	12.4	1E-04	0.7	3E-01	5.5	0.5	12.5	*	*
327	337	'DAFPDKVAIQL'	327 - 337	0.0	0.9	1.2	1.1	0.9	7.9	4E-14	0.5	2E-04	5.0	1.0	4.0	2E-14	0.5	9E-05	5.0	1.0	4.0	1E-12	0.5	3E-05	5.0	1.0	4.0		

				Intrinsic fits						GlyPa'						GlyPb'						GlyPb-G6P'						Significantly different peptides	
Start	End	Sequence	Residues	k_{int1}	β_1	k_{int2}	N_1	β_2	N_2	k_{exp1}	β_1	k_{exp2}	N_1	β_2	N_2	k_{exp1}	β_1	k_{exp2}	N_1	β_2	N_2	k_{exp1}	β_1	k_{exp2}	N_1	β_2	N_2	GlyPa vs GlyPb	GlyPb vs GlyP:G6P
330	337	'PDKVAIQL'	330 - 337	0.0	0.8	1.1	1.1	0.9	5.9	3E-07	0.7	4E-05	3.0	1.0	3.0	6E-07	0.7	4E-05	3.0	1.0	3.0	4E-14	0.9	4E-05	3.0	1.0	3.0		
337	348	'LNDTHPSLAIPE'	337 - 348	0.0	0.6	2.1	1.0	1.0	8.0	2E-14	0.5	3E-04	5.0	1.0	4.0	4E-07	0.5	7E-05	5.0	1.0	4.0	8E-12	0.5	2E-04	5.0	1.0	4.0	*	
338	344	'NDTHPSL'	338 - 344	0.0	0.7	4.0	1.0	1.0	4.0	2E-14	0.5	4E-02	3.0	1.0	2.0	2E-14	0.5	2E-03	3.0	1.0	2.0	4E-14	0.5	3E-03	3.0	1.0	2.0		
338	349	'NDTHPSLAIPEL'	338 - 349	0.0	0.7	1.7	1.0	1.0	8.0	4E-14	0.5	4E-04	5.0	1.0	4.0	2E-06	0.5	7E-05	5.0	1.0	4.0	5E-05	1.0	3E-04	5.0	1.0	4.0	*	*
338	350	'NDTHPSLAIPELM'	338 - 350	0.0	0.7	1.4	1.0	1.0	9.0	3E-14	0.5	1E-04	5.5	1.0	4.5	2E-07	1.0	1E-04	5.5	1.0	4.5	1E-04	1.0	5E-05	5.5	1.0	4.5	*	
350	355	'MRVLVD'	350 - 355	3.0	0.8	0.4	0.6	1.0	4.4	2E-14	1.0	2E-14	0.3	1.0	4.7	2E-14	1.0	2E-14	0.4	1.0	4.6	4E-14	1.0	2E-14	0.3	1.0	4.7		
350	356	'MRVLVDL'	350 - 356	0.0	0.7	0.8	1.0	1.0	5.0	3E-14	1.0	1E-10	3.5	1.0	2.5	2E-14	1.0	5E-11	3.5	1.0	2.5	4E-14	1.0	8E-10	3.5	1.0	2.5		
351	356	'RVLVDL'	351 - 356	0.0	0.8	0.6	1.0	1.0	4.0	9E-14	1.0	2E-10	3.0	1.0	2.0	3E-13	1.0	4E-10	3.0	1.0	2.0	4E-13	1.0	5E-10	3.0	1.0	2.0		
356	364	'LERLDWDKA'	356 - 364	1.3	0.9	0.1	6.9	0.9	1.1	3E-02	1.0	8E-04	3.4	0.5	4.6	4E-02	1.0	6E-04	3.4	0.6	4.6	3E-02	1.0	6E-04	3.4	0.6	4.6		
356	366	'LERLDWDKAWE'	356 - 366	0.0	0.9	1.4	1.0	1.0	9.0	3E-14	0.5	1E-02	5.5	1.0	4.5	2E-14	0.5	1E-02	5.5	1.0	4.5	2E-13	0.5	7E-03	5.5	1.0	4.5		
357	364	'ERLDWDKA'	357 - 364	1.4	0.9	0.1	5.9	0.9	1.1	9E-02	1.0	2E-03	2.9	0.5	4.1	1E-01	1.0	2E-03	2.9	0.6	4.1	7E-02	1.0	1E-03	2.9	0.7	4.1		
357	365	'ERLDWDKAW'	357 - 365	0.0	0.9	1.5	1.0	1.0	7.0	1E-03	0.5	1E-01	4.5	1.0	3.5	1E-03	0.5	1E-01	4.5	1.0	3.5	1E-03	0.5	8E-02	4.5	1.0	3.5		*
357	366	'ERLDWDKAWE'	357 - 366	0.0	0.9	1.4	1.0	1.0	8.0	4E-05	0.5	2E-02	5.0	1.0	4.0	2E-14	0.5	2E-02	5.0	1.0	4.0	1E-13	0.5	1E-02	5.0	1.0	4.0		
367	373	'VTVKTCA'	367 - 373	0.1	0.7	1.9	1.0	1.0	5.0	4E-14	1.0	5E-10	3.5	1.0	2.5	3E-14	1.0	9E-11	3.5	1.0	2.5	6E-14	1.0	1E-09	3.5	1.0	2.5		
369	384	'VKTCAYTNHTVLPEAL'	369 - 384	2.4	1.0	0.0	13.0	0.6	1.0	1E-01	1.0	1E-03	6.5	0.5	7.5	1E-02	1.0	9E-06	6.5	0.5	7.5	1E-02	1.0	3E-07	7.3	0.5	6.7	*	
374	380	'YTNHTVL'	374 - 380	0.0	0.5	4.4	1.0	1.0	5.0	4E-03	0.9	2E-01	3.5	0.9	2.5	2E-08	1.0	1E-02	2.5	1.0	3.5	2E-09	1.0	1E-02	2.4	1.0	3.6	*	
374	383	'YTNHTVLPEA'	374 - 383	0.0	0.5	2.1	1.0	1.0	7.0	4E-03	0.6	2E-01	2.6	1.0	5.4	2E-03	0.5	3E-02	3.5	1.0	4.5	1E-07	0.6	2E-02	1.6	0.6	6.4	*	
374	384	'YTNHTVLPEAL'	374 - 384	0.0	0.6	2.0	1.0	1.0	8.0	3E-03	0.7	2E-01	5.0	0.8	4.0	6E-04	0.7	3E-02	5.0	1.0	4.0	8E-04	0.9	3E-02	5.0	1.0	4.0	*	
375	380	'TNHTVL'	375 - 380	0.0	0.4	5.9	1.0	1.0	4.0	4E-03	0.6	3E-01	3.0	0.6	2.0	1E-03	0.5	1E-02	3.0	1.0	2.0	3E-11	1.0	2E-02	2.7	1.0	2.3	*	*
384	391	'LERWPVHL'	384 - 391	0.1	0.7	1.7	1.7	0.7	4.3	3E-04	0.5	2E-01	3.7	0.5	2.3	7E-04	0.5	1E-01	3.9	1.0	2.1	6E-04	0.5	1E-01	3.9	1.0	2.1	*	
385	391	'ERWPVHL'	385 - 391	2.0	0.7	0.1	3.2	0.7	1.8	3E-01	0.7	6E-04	1.6	0.6	3.4	2E-01	0.6	3E-04	1.7	0.7	3.3	2E-01	0.7	5E-04	1.6	0.7	3.4		
385	393	'ERWPVHLLE'	385 - 393	0.0	0.6	1.2	1.0	1.0	6.0	3E-14	0.6	1E-03	4.0	1.0	3.0	5E-11	0.6	9E-04	4.0	1.0	3.0	2E-11	0.6	1E-03	4.0	1.0	3.0	*	
392	402	'LETLLPRHLQI'	392 - 402	0.0	0.7	1.5	1.0	1.0	8.0	4E-07	1.0	3E-04	5.0	1.0	4.0	9E-07	1.0	4E-04	5.0	1.0	4.0	3E-06	1.0	4E-04	5.0	1.0	4.0		
392	404	'LETLLPRHLQIIY'	392 - 404	0.0	0.7	1.1	1.1	0.9	9.9	3E-07	0.7	3E-04	6.0	1.0	5.0	4E-07	0.9	4E-04	6.0	1.0	5.0	2E-04	1.0	6E-05	6.0	1.0	5.0		
392	405	'LETLLPRHLQIIYE'	392 - 405	0.0	0.7	1.0	1.0	1.0	11.0	9E-05	1.0	8E-05	6.5	1.0	5.5	2E-05	1.0	1E-05	6.5	1.0	5.5	1E-04	1.0	1E-04	6.5	1.0	5.5		
394	402	'TLLPRHLQI'	394 - 402	0.0	0.7	1.5	1.0	1.0	6.0	3E-14	0.5	8E-04	4.0	1.0	3.0	1E-13	0.5	1E-03	4.0	1.0	3.0	2E-14	0.5	5E-04	4.0	1.0	3.0		
394	404	'TLLPRHLQIIY'	394 - 404	0.0	0.6	1.0	1.0	1.0	8.0	2E-14	1.0	4E-11	5.0	1.0	4.0	3E-14	1.0	2E-10	5.0	1.0	4.0	2E-14	1.0	5E-11	5.0	1.0	4.0		
394	405	'TLLPRHLQIIYE'	394 - 405	0.0	0.7	1.0	1.0	1.0	9.0	3E-14	1.0	2E-09	5.5	1.0	4.5	2E-14	1.0	7E-14	5.5	1.0	4.5	3E-14	1.0	4E-10	5.5	1.0	4.5		

				Intrinsic fits						GlyPa'						GlyPb'						GlyPb-G6P'						Significantly different peptides	
Start	End	Sequence	Residues	k_{int1}	β_1	k_{int2}	N_1	β_2	N_2	k_{exp1}	β_1	k_{exp2}	N_1	β_2	N_2	k_{exp1}	β_1	k_{exp2}	N_1	β_2	N_2	k_{exp1}	β_1	k_{exp2}	N_1	β_2	N_2	GlyPa vs GlyPb	GlyPb vs GlyP:G6P
396	404	'LPRHLQIIY'	396 - 404	0.0	0.6	1.3	1.0	1.0	6.0	3E-12	0.5	3E-05	4.0	1.0	3.0	2E-05	0.5	6E-08	4.0	0.5	3.0	2E-14	1.0	2E-11	4.0	1.0	3.0		
396	405	'LPRHLQIIYE'	396 - 405	0.0	0.6	1.2	1.0	1.0	7.0	4E-06	0.5	7E-05	4.5	1.0	3.5	4E-11	0.5	4E-05	4.5	1.0	3.5	4E-06	1.0	2E-04	4.5	1.0	3.5		
397	404	'PRHLQIIY'	397 - 404	0.0	0.6	1.3	1.0	1.0	6.0	2E-14	1.0	1E-10	3.0	1.0	3.0	2E-14	1.0	4E-11	3.0	1.0	3.0	6E-14	1.0	4E-09	3.0	1.0	3.0		
397	405	'PRHLQIIYE'	397 - 405	0.0	0.6	1.2	1.0	1.0	7.0	2E-14	1.0	1E-11	3.5	1.0	3.5	2E-14	1.0	2E-11	3.5	1.0	3.5	2E-14	1.0	4E-14	3.5	1.0	3.5		
403	425	'IYEINQRFLNRVAAAFPGDVDRL'	403 - 425	1.8	0.7	0.0	19.5	0.7	1.5	8E-03	1.0	2E-14	9.8	0.5	11.2	8E-03	1.0	2E-14	9.8	0.5	11.2	6E-03	1.0	2E-14	9.8	0.5	11.2		
405	418	'EINQRFLNRVAAAF'	405 - 418	2.1	0.9	0.0	11.9	0.7	1.1	1E-04	1.0	2E-14	5.9	0.8	7.1	7E-05	1.0	4E-07	5.9	0.9	7.1	7E-05	1.0	6E-08	5.9	0.9	7.1		
405	425	'EINQRFLNRVAAAFPGDVDRL'	405 - 425	0.0	0.7	1.9	1.5	0.7	17.5	2E-14	0.5	7E-03	10.2	1.0	8.8	2E-14	0.5	7E-03	10.2	1.0	8.8	2E-14	0.5	6E-03	10.2	1.0	8.8		
405	430	'EINQRFLNRVAAAFPGDVDRLRRMSL'	405 - 430	2.1	0.6	0.0	22.1	0.7	1.9	3E-03	1.0	2E-14	11.1	0.5	12.9	2E-03	1.0	2E-14	11.1	0.5	12.9	2E-03	1.0	2E-14	11.1	0.5	12.9		
406	415	'INQRFLNRVA'	406 - 415	2.7	0.9	0.0	7.9	0.7	1.1	7E-05	1.0	2E-14	3.9	0.5	5.1	5E-05	1.0	2E-14	3.9	0.6	5.1	3E-05	1.0	7E-08	3.9	0.6	5.1		
406	418	'INQRFLNRVAAAF'	406 - 418	0.0	0.8	2.4	1.0	1.0	11.0	9E-08	1.0	9E-05	6.5	1.0	5.5	3E-08	1.0	3E-05	6.5	1.0	5.5	3E-12	1.0	1E-06	6.5	1.0	5.5		
406	425	'INQRFLNRVAAAFPGDVDRL'	406 - 425	2.0	0.8	0.0	16.7	0.7	1.3	9E-03	1.0	2E-14	8.4	0.5	9.6	9E-03	1.0	2E-14	8.4	0.5	9.6	7E-03	1.0	2E-14	8.4	0.5	9.6		
406	430	'INQRFLNRVAAAFPGDVDRLRRMSL'	406 - 430	2.2	0.7	0.0	21.6	0.8	1.4	2E-03	1.0	2E-14	10.8	0.5	12.2	2E-03	1.0	3E-14	10.8	0.5	12.2	2E-03	1.0	2E-14	10.8	0.5	12.2		
407	430	'NQRFLNRVAAAFPGDVDRLRRMSL'	407 - 430	2.1	0.7	0.0	20.6	0.8	1.4	3E-03	1.0	2E-14	10.3	0.5	11.7	2E-03	1.0	4E-14	10.3	0.5	11.7	2E-03	1.0	2E-14	10.3	0.5	11.7		
408	425	'QRFLNRVAAAFPGDVDRL'	408 - 425	0.0	0.8	1.8	1.2	0.8	14.8	2E-14	0.5	3E-02	8.6	1.0	7.4	2E-14	0.5	3E-02	8.6	1.0	7.4	2E-14	0.5	2E-02	8.6	1.0	7.4		
411	430	'LNRVAAAFPGDVDRLRRMSL'	411 - 430	0.0	0.8	2.1	1.3	0.8	16.7	2E-14	0.5	6E-03	9.7	1.0	8.3	2E-14	0.5	6E-03	9.7	1.0	8.3	2E-14	0.5	4E-03	9.7	1.0	8.3		
416	425	'AAFPGDVDRL'	416 - 425	0.0	0.8	1.6	1.2	0.9	6.8	1E-03	0.5	4E-01	4.6	1.0	3.4	5E-04	0.5	4E-01	4.6	0.6	3.4	7E-04	0.5	4E-01	4.6	0.7	3.4		
419	425	'PGDVDRL'	419 - 425	0.0	0.7	1.5	1.1	0.9	4.9	2E-04	0.5	7E-02	2.6	1.0	2.4	2E-04	0.6	6E-02	2.6	1.0	2.4	2E-04	0.6	6E-02	2.6	1.0	2.4		
431	441	'VEEGAVKRINM'	431 - 441	0.0	0.8	1.5	1.1	0.9	8.9	2E-05	0.5	4E-01	4.6	1.0	5.4	2E-12	0.6	5E-01	4.7	1.0	5.3	2E-06	0.6	5E-01	4.8	0.5	5.2		
431	444	'VEEGAVKRINMAHL'	431 - 444	0.1	0.8	2.1	1.4	0.8	11.6	2E-14	0.5	3E-01	7.2	1.0	5.8	2E-14	0.5	3E-01	7.2	1.0	5.8	2E-14	0.5	3E-01	7.2	1.0	5.8		
432	441	'EEGAVKRINM'	432 - 441	0.0	0.8	1.6	1.1	0.9	7.9	7E-05	0.5	6E-01	4.3	1.0	4.7	3E-14	0.6	7E-01	4.3	1.0	4.7	1E-06	0.5	6E-01	4.4	0.5	4.6		
445	460	'CIAGSHAVNGVARIHS'	445 - 460	2.5	0.7	0.1	13.4	0.7	1.6	1E-03	1.0	2E-14	6.7	0.5	8.3	2E-04	1.0	2E-14	6.7	0.5	8.3	2E-04	1.0	2E-14	6.7	0.5	8.3		
445	461	'CIAGSHAVNGVARIHSE'	445 - 461	0.0	0.6	2.6	1.0	1.0	15.0	4E-14	0.5	1E-03	8.5	1.0	7.5	2E-14	0.5	2E-04	8.5	1.0	7.5	3E-14	0.5	2E-04	8.5	1.0	7.5		
445	462	'CIAGSHAVNGVARIHSEI'	445 - 462	0.0	0.6	2.7	1.0	1.0	16.0	3E-14	0.5	1E-03	9.0	1.0	8.0	4E-14	0.5	2E-04	9.0	1.0	8.0	3E-14	0.5	2E-04	9.0	1.0	8.0		
445	463	'CIAGSHAVNGVARIHSEIL'	445 - 463	0.0	0.6	2.3	1.0	1.0	17.0	2E-14	0.5	1E-03	9.5	1.0	8.5	4E-14	0.5	3E-04	9.5	1.0	8.5	4E-14	0.5	3E-04	9.5	1.0	8.5		
445	468	'CIAGSHAVNGVARIHSEILKKTIF'	445 - 468	0.0	0.6	1.9	1.0	1.0	22.0	4E-14	0.5	8E-04	12.0	1.0	11.0	4E-14	0.5	3E-04	12.0	1.0	11.0	4E-14	0.5	3E-04	12.0	1.0	11.0		
445	471	'CIAGSHAVNGVARIHSEILKKTIFKDF'	445 - 471	1.9	0.9	0.0	24.9	0.6	1.1	8E-04	1.0	8E-14	12.5	0.5	13.5	4E-04	1.0	3E-14	12.5	0.5	13.5	3E-04	1.0	2E-14	12.5	0.5	13.5		
464	471	'KKTIFKDF'	464 - 471	0.0	0.8	1.7	1.0	1.0	6.0	3E-04	0.5	2E-03	4.0	1.0	3.0	3E-04	0.5	3E-03	4.0	1.0	3.0	8E-04	0.5	7E-04	4.0	1.0	3.0		
472	491	'YELEPHKFQNKTNGITPRRW'	472 - 491	2.5	0.8	0.0	15.8	0.7	1.2	4E-04	1.0	2E-14	7.9	0.5	9.1	3E-04	1.0	2E-14	7.9	0.5	9.1	3E-04	1.0	2E-14	7.9	0.5	9.1		

Start	End	Sequence	Residues	Intrinsic fits						GlyPa'						GlyPb'						GlyPb-G6P'						Significantly different peptides	
				k_{int1}	β_1	k_{int2}	N_1	β_2	N_2	k_{exp1}	β_1	k_{exp2}	N_1	β_2	N_2	k_{exp1}	β_1	k_{exp2}	N_1	β_2	N_2	k_{exp1}	β_1	k_{exp2}	N_1	β_2	N_2	GlyPa vs GlyPb	GlyPb vs GlyP:G6P
476	494	'PHKFQNKTNGITPRRWLVL'	476 - 494	0.0	0.6	2.3	1.1	0.9	15.9	3E-14	0.5	1E-03	8.0	1.0	8.0	2E-14	0.5	4E-04	8.0	1.0	8.0	2E-14	0.5	2E-03	8.0	1.0	8.0		
492	499	'LVLCNPGL'	492 - 499	0.0	0.5	1.2	1.0	1.0	5.0	3E-13	0.5	1E-03	3.5	1.0	2.5	2E-14	0.5	3E-04	3.5	1.0	2.5	4E-14	0.5	1E-04	3.5	1.0	2.5		
495	501	'CNPGLAE'	495 - 501	0.0	0.7	1.8	1.0	1.0	4.0	2E-04	0.5	3E-03	3.0	1.0	2.0	2E-14	0.5	3E-04	3.0	1.0	2.0	1E-04	0.5	7E-05	3.0	1.0	2.0	*	
498	503	'GLAEII'	498 - 503	0.0	0.8	0.9	1.0	1.0	4.0	3E-14	1.0	2E-09	3.0	1.0	2.0	3E-14	1.0	9E-10	3.0	1.0	2.0	2E-14	1.0	4E-11	3.0	1.0	2.0	*	
500	505	'AEIIAE'	500 - 505	0.0	0.7	0.6	1.0	1.0	4.0	2E-14	0.5	4E-05	3.0	0.5	2.0	1E-06	0.8	1E-04	3.0	1.0	2.0	3E-06	1.0	3E-04	3.0	1.0	2.0		
503	510	'IAERIGEE'	503 - 510	0.0	0.9	1.5	1.0	1.0	6.0	2E-14	0.5	7E-03	4.0	1.0	3.0	2E-14	0.5	7E-03	4.0	1.0	3.0	4E-14	0.6	7E-03	4.0	1.0	3.0		
510	515	'EYISDL'	510 - 515	0.0	0.6	1.5	1.0	1.0	4.0	9E-07	0.5	2E-03	2.2	0.7	2.8	1E-06	0.6	5E-03	3.0	1.0	2.0	7E-11	0.7	5E-03	3.0	1.0	2.0		*
511	517	'YISDLDQ'	511 - 517	0.0	0.6	1.3	1.0	1.0	5.0	8E-04	0.5	1E-02	3.5	1.0	2.5	5E-04	0.5	9E-03	3.5	1.0	2.5	9E-04	0.5	6E-03	3.5	1.0	2.5		
516	523	'DQLRKLLS'	516 - 523	0.0	0.8	1.1	1.0	1.0	6.0	2E-14	0.5	6E-03	4.0	1.0	3.0	2E-14	0.5	6E-03	4.0	1.0	3.0	2E-14	0.5	4E-03	4.0	1.0	3.0		*
524	529	'YVDDEA'	524 - 529	0.0	0.8	1.3	1.1	0.9	3.9	1E-06	0.8	3E-02	1.6	0.5	3.4	2E-03	1.0	5E-02	2.9	1.0	2.1	6E-04	1.0	3E-02	1.9	1.0	3.1		
524	530	'YVDDEAF'	524 - 530	0.0	0.9	1.3	1.0	1.0	5.0	6E-05	0.7	2E-02	3.0	0.9	3.0	2E-07	0.7	3E-02	3.0	0.6	3.0	2E-07	0.7	2E-02	2.9	0.8	3.1		
530	545	'FIRDVAKVKQENKLKF'	530 - 545	1.6	0.9	0.0	13.8	0.7	1.2	4E-05	1.0	2E-07	6.9	0.9	8.1	3E-05	0.9	3E-07	6.9	0.9	8.1	2E-10	1.0	2E-14	6.9	1.0	8.1		
530	548	'FIRDVAKVKQENKLKFAAY'	530 - 548	0.0	0.7	1.7	1.2	0.8	16.8	2E-14	0.8	8E-05	9.6	1.0	8.4	2E-14	0.8	4E-05	9.6	1.0	8.4	4E-14	0.8	4E-05	9.6	1.0	8.4		
531	545	'IRDVAKVKQENKLKF'	531 - 545	1.7	0.9	0.0	12.8	0.7	1.2	3E-05	1.0	3E-07	6.4	0.9	7.6	3E-10	1.0	6E-14	6.4	1.0	7.6	3E-10	1.0	3E-14	6.4	1.0	7.6		
531	548	'IRDVAKVKQENKLKFAAY'	531 - 548	0.0	0.8	1.8	1.2	0.8	15.8	2E-14	0.7	4E-05	9.1	1.0	7.9	1E-06	0.7	4E-05	9.1	1.0	7.9	2E-14	0.8	5E-05	9.1	1.0	7.9		
548	562	'YLEREYKVHINPNSL'	548 - 562	2.0	1.0	0.0	12.0	0.7	1.0	5E-03	1.0	2E-05	6.0	0.5	7.0	6E-03	1.0	2E-14	6.0	0.5	7.0	5E-03	1.0	2E-04	6.0	0.5	7.0		
549	562	'LEREYKVHINPNSL'	549 - 562	2.3	0.9	0.0	10.9	0.7	1.1	8E-03	1.0	3E-04	5.5	0.5	6.5	1E-02	1.0	6E-05	5.5	0.5	6.5	7E-03	1.0	4E-04	5.5	0.5	6.5		
549	563	'LEREYKVHINPNSLF'	549 - 563	0.0	0.7	2.1	1.0	1.0	12.0	9E-05	0.5	9E-03	7.0	1.0	6.0	9E-05	0.5	1E-02	7.0	1.0	6.0	3E-04	0.5	9E-03	7.0	1.0	6.0		
550	562	'EREYKVHINPNSL'	550 - 562	2.5	0.9	0.0	9.9	0.7	1.1	8E-03	1.0	2E-04	5.0	0.5	6.0	1E-02	1.0	2E-04	5.0	0.5	6.0	6E-03	1.0	5E-04	5.0	0.5	6.0		
550	563	'EREYKVHINPNSLF'	550 - 563	0.0	0.7	2.3	1.0	1.0	11.0	2E-04	0.5	9E-03	6.5	1.0	5.5	2E-04	0.5	1E-02	6.5	1.0	5.5	4E-04	0.5	8E-03	6.5	1.0	5.5		
553	562	'YKVHINPNSL'	553 - 562	0.0	0.7	3.1	1.1	0.9	6.9	9E-04	0.5	2E-03	4.5	1.0	3.5	2E-04	0.7	9E-03	4.5	1.0	3.5	3E-04	0.8	9E-03	4.5	1.0	3.5		
563	572	'FDVQVKRIHE'	563 - 572	1.5	0.9	0.0	7.9	0.7	1.1	1E-02	1.0	2E-14	3.9	0.5	5.1	2E-03	1.0	2E-14	3.9	0.5	5.1	1E-03	1.0	1E-13	3.9	0.5	5.1	*	
563	578	'FDVQVKRIHEYKRQLL'	563 - 578	1.8	1.0	0.0	14.0	0.7	1.0	3E-03	1.0	2E-14	7.0	0.5	8.0	7E-04	1.0	2E-14	7.0	0.5	8.0	8E-04	1.0	1E-11	7.0	0.5	8.0	*	
563	580	'FDVQVKRIHEYKRQLLNC'	563 - 580	1.7	0.8	0.0	15.7	0.7	1.3	2E-03	1.0	2E-14	7.8	0.5	9.2	6E-04	1.0	2E-14	7.8	0.5	9.2	6E-04	1.0	2E-14	7.8	0.5	9.2	*	
563	581	'FDVQVKRIHEYKRQLLNCL'	563 - 581	0.1	0.7	2.0	1.9	0.7	16.1	2E-14	0.5	2E-03	9.9	1.0	8.1	2E-14	0.5	7E-04	9.9	1.0	8.1	3E-14	0.5	8E-04	9.8	1.0	8.1	*	
564	580	'DVQVKRIHEYKRQLLNC'	564 - 580	0.0	0.7	1.6	1.3	0.8	14.7	2E-14	0.5	2E-03	8.6	1.0	7.4	3E-14	0.5	6E-04	8.6	1.0	7.4	2E-14	0.5	6E-04	8.6	1.0	7.4	*	
581	586	'LHVITL'	581 - 586	0.0	0.6	1.3	1.0	1.0	4.0	2E-12	1.0	4E-08	3.0	1.0	2.0	3E-14	1.0	9E-11	3.0	1.0	2.0	8E-14	1.0	2E-09	3.0	1.0	2.0		
587	604	'YNRIKKEPNKFVVPRTVM'	587 - 604	0.0	0.7	1.7	1.0	1.0	14.0	2E-14	0.5	5E-02	8.0	1.0	7.0	2E-14	0.5	5E-02	8.0	1.0	7.0	2E-14	0.5	4E-02	8.0	1.0	7.0		

Start	End	Sequence	Residues	Intrinsic fits						GlyPa'						GlyPb'						GlyPb-G6P'						Significantly different peptides	
				k_{int1}	β_1	k_{int2}	N_1	β_2	N_2	k_{exp1}	β_1	k_{exp2}	N_1	β_2	N_2	k_{exp1}	β_1	k_{exp2}	N_1	β_2	N_2	k_{exp1}	β_1	k_{exp2}	N_1	β_2	N_2	GlyPa vs GlyPb	GlyPb vs GlyP:G6P
605	618	'IGGKAAPGYHMAKM'	605 - 618	0.0	0.8	2.7	1.0	1.0	11.0	1E-03	0.5	4E-01	6.5	1.0	5.5	3E-04	0.5	2E-01	6.5	1.0	5.5	3E-04	0.5	1E-01	6.5	1.0	5.5	*	
606	618	'GGKAAPGYHMAKM'	606 - 618	0.0	0.8	2.9	1.0	1.0	10.0	9E-04	0.5	3E-01	6.0	1.0	5.0	7E-05	0.5	1E-01	6.0	1.0	5.0	4E-05	0.5	9E-02	6.0	1.0	5.0		
619	624	'IIKLIT'	619 - 624	0.0	0.8	0.5	1.0	1.0	4.0	3E-14	1.0	2E-10	3.0	1.0	2.0	3E-13	1.0	3E-09	3.0	1.0	2.0	6E-13	1.0	2E-09	3.0	1.0	2.0		
619	625	'IIKLITA'	619 - 625	0.2	0.9	0.8	2.8	0.7	3.2	6E-13	0.5	9E-04	4.4	1.0	1.6	3E-14	0.5	4E-04	4.4	1.0	1.6	6E-05	1.0	6E-05	4.4	1.0	1.6		
619	640	'IIKLITAIGDVVNHDPVVGDRL'	619 - 640	0.0	0.6	1.0	0.9	1.0	19.1	2E-05	0.5	2E-02	10.5	1.0	9.5	1E-04	0.5	2E-02	10.5	1.0	9.5	2E-04	0.5	1E-02	10.5	1.0	9.5		
623	628	'ITAIGD'	623 - 628	0.6	1.0	2.2	3.3	0.9	1.7	3E-14	0.5	1E+00	4.2	1.0	0.8	2E-14	0.5	1E+00	4.2	1.0	0.8	3E-14	0.5	4E-03	4.2	1.0	0.8		
623	640	'ITAIGDVVNHDPVVGDRL'	623 - 640	1.3	1.0	0.0	15.0	0.5	1.0	2E-02	1.0	2E-04	7.5	0.5	8.5	2E-02	1.0	8E-05	7.5	0.5	8.5	2E-02	1.0	1E-04	7.5	0.5	8.5		
623	642	'ITAIGDVVNHDPVVGDRLRV'	623 - 642	0.0	0.6	1.3	1.0	1.0	17.0	2E-14	0.5	1E-02	9.5	1.0	8.5	1E-08	0.5	2E-02	9.5	1.0	8.5	4E-05	0.5	1E-02	9.5	1.0	8.5		
626	640	'IGDVVNHDPVVGDRL'	626 - 640	1.3	1.0	0.0	12.1	0.5	0.9	1E-02	1.0	7E-04	6.0	0.5	7.0	2E-02	1.0	5E-04	6.0	0.5	7.0	1E-02	1.0	8E-04	6.0	0.5	7.0		
627	640	'GDVVNHDPVVGDRL'	627 - 640	1.3	1.0	0.0	11.1	0.5	0.9	2E-02	1.0	4E-05	5.5	0.5	6.5	2E-02	1.0	8E-12	5.5	0.5	6.5	2E-02	1.0	8E-05	5.5	0.5	6.5		*
628	640	'DVVNHDPVVGDRL'	628 - 640	0.0	0.5	1.1	0.9	1.0	10.1	3E-04	0.5	3E-02	5.9	1.0	5.1	6E-04	0.5	3E-02	5.9	1.0	5.1	2E-04	0.5	2E-02	5.9	1.0	5.1		*
645	652	'LENYRVSL'	645 - 652	0.0	0.8	2.2	1.0	1.0	6.0	5E-14	0.5	6E-03	4.0	1.0	3.0	3E-14	0.5	4E-03	4.0	1.0	3.0	3E-14	0.5	3E-03	4.0	1.0	3.0		
648	661	'YRVSLAEKVIPAAD'	648 - 661	0.5	1.0	1.8	4.9	0.8	7.1	3E-14	0.5	1E-03	8.5	1.0	3.5	3E-13	0.5	1E-03	8.5	1.0	3.5	2E-14	0.5	1E-03	8.5	1.0	3.5		
653	661	'AEKVIPAAD'	653 - 661	0.4	1.0	1.7	3.3	0.9	3.7	4E-14	0.5	5E-04	5.2	1.0	1.8	2E-14	0.5	2E-04	5.2	0.6	1.8	4E-14	0.5	1E-04	5.2	1.0	1.8		
653	662	'AEKVIPAADL'	653 - 662	0.0	0.7	1.2	1.0	1.0	7.0	4E-14	0.5	6E-05	4.5	1.0	3.5	4E-14	0.8	5E-05	4.5	1.0	3.5	4E-07	0.5	1E-05	4.5	0.5	3.5		
653	664	'AEKVIPAADLSE'	653 - 664	0.0	0.7	1.2	1.1	0.9	8.9	2E-14	0.5	6E-05	5.5	1.0	4.5	3E-14	0.5	9E-05	5.5	1.0	4.5	3E-12	0.5	3E-05	5.5	1.0	4.5		*
655	661	'KVIPAAD'	655 - 661	0.4	1.0	1.7	3.3	0.9	1.7	3E-14	0.5	2E-03	4.1	1.0	0.9	4E-14	0.5	1E-03	4.1	1.0	0.9	9E-12	0.5	6E-04	4.1	1.0	0.9		
662	681	'LSEQISTAGTEASGTGNMKF'	662 - 681	3.0	0.9	0.0	17.9	0.9	1.1	1E-02	1.0	2E-14	9.0	0.5	10.0	2E-03	1.0	2E-13	9.0	0.5	10.0	2E-03	1.0	2E-14	9.0	0.5	10.0	*	*
663	681	'SEQISTAGTEASGTGNMKF'	663 - 681	3.0	0.9	0.0	16.9	0.9	1.1	2E-02	1.0	2E-14	8.5	0.5	9.5	2E-03	1.0	2E-14	8.5	0.5	9.5	2E-03	1.0	2E-14	8.5	0.5	9.5	*	
665	681	'QISTAGTEASGTGNMKF'	665 - 681	3.1	0.9	0.0	14.9	0.8	1.1	2E-02	1.0	2E-14	7.5	0.5	8.5	3E-03	1.0	2E-14	7.5	0.5	8.5	2E-03	1.0	2E-14	7.5	0.5	8.5	*	*
666	681	'ISTAGTEASGTGNMKF'	666 - 681	3.4	1.0	0.0	14.0	0.9	1.0	3E-02	1.0	2E-14	7.0	0.5	8.0	2E-03	1.0	4E-14	7.0	0.5	8.0	3E-03	1.0	2E-14	7.0	0.5	8.0	*	
682	687	'MLNGAL'	682 - 687	0.0	0.7	2.6	1.0	1.0	4.0	2E-06	0.5	4E-05	3.0	1.0	2.0	3E-13	1.0	1E-07	3.0	1.0	2.0	3E-14	1.0	2E-09	3.0	1.0	2.0		
682	692	'MLNGALTIGTM'	682 - 692	0.0	0.7	1.5	1.0	1.0	9.0	2E-14	0.5	1E-04	5.5	1.0	4.5	2E-14	0.5	5E-05	5.5	1.0	4.5	2E-14	0.5	4E-05	5.5	1.0	4.5		
682	696	'MLNGALTIGTMDGAN'	682 - 696	1.9	1.0	0.0	13.0	0.8	1.0	1E-03	1.0	3E-14	6.5	0.5	7.5	3E-04	1.0	3E-14	6.5	0.5	7.5	3E-04	1.0	2E-14	6.5	0.5	7.5		
686	696	'ALTIGTMDGAN'	686 - 696	0.0	0.8	1.6	1.0	1.0	9.0	2E-14	0.5	2E-02	5.5	1.0	4.5	2E-14	0.5	2E-03	5.5	1.0	4.5	3E-14	0.5	2E-03	5.5	1.0	4.5		
688	698	'TIGTMDGANVE'	688 - 698	0.0	0.8	2.0	1.0	1.0	9.0	2E-14	0.5	4E-02	5.5	1.0	4.5	2E-14	0.5	3E-03	5.5	1.0	4.5	2E-14	0.5	2E-03	5.5	1.0	4.5	*	
689	696	'IGTMDGAN'	689 - 696	0.0	0.9	2.4	1.0	1.0	6.0	2E-14	0.5	1E-01	4.0	1.0	3.0	2E-14	0.5	9E-03	4.0	1.0	3.0	2E-14	0.5	7E-03	4.0	1.0	3.0	*	*
697	702	'VEMAEE'	697 - 702	0.0	0.9	1.8	1.0	1.0	4.0	4E-14	0.5	2E-04	3.0	1.0	2.0	4E-14	0.5	1E-04	3.0	1.0	2.0	2E-14	1.0	6E-10	3.0	1.0	2.0		

				Intrinsic fits						GlyPa'						GlyPb'						GlyPb-G6P'						Significantly different peptides	
Start	End	Sequence	Residues	k_{int1}	β_1	k_{int2}	N_1	β_2	N_2	k_{exp1}	β_1	k_{exp2}	N_1	β_2	N_2	k_{exp1}	β_1	k_{exp2}	N_1	β_2	N_2	k_{exp1}	β_1	k_{exp2}	N_1	β_2	N_2	GlyPa vs GlyPb	GlyPb vs GlyP:G6P
699	708	'MAEEAGEENF'	699 - 708	2.1	1.0	0.0	8.0	0.9	1.0	5E-02	1.0	2E-14	4.0	0.5	5.0	5E-02	1.0	2E-14	4.0	0.5	5.0	3E-02	1.0	2E-14	4.0	0.5	5.0		
700	708	'AEEAGEENF'	700 - 708	0.0	0.9	2.0	1.0	1.0	7.0	2E-14	0.5	3E-02	4.5	1.0	3.5	2E-14	0.5	2E-02	4.5	1.0	3.5	2E-14	0.5	1E-02	4.5	1.0	3.5		
702	708	'EAGEENF'	702 - 708	0.0	0.9	2.2	1.0	1.0	5.0	3E-04	0.5	2E-01	3.5	1.0	2.5	7E-05	0.5	1E-01	3.5	1.0	2.5	7E-05	0.5	1E-01	3.5	1.0	2.5		
703	708	'AGEENF'	703 - 708	0.0	0.9	2.4	1.0	1.0	4.0	4E-04	0.5	1E-01	3.0	1.0	2.0	3E-04	0.5	1E-01	3.0	1.0	2.0	4E-04	0.5	7E-02	3.0	1.0	2.0		*
709	715	'FIFGMRV'	709 - 715	0.0	0.7	1.5	1.0	1.0	5.0	4E-04	0.5	4E-03	3.5	1.0	2.5	3E-11	0.5	8E-03	3.5	1.0	2.5	7E-04	0.7	2E-03	3.5	1.0	2.5		
709	717	'FIFGMRVED'	709 - 717	1.9	0.7	0.6	3.9	0.9	4.1	2E-02	1.0	7E-04	1.9	0.5	6.1	2E-02	1.0	6E-04	1.9	0.5	6.1	1E-02	1.0	6E-04	1.9	0.6	6.1		
713	730	'MRVEDVDRLDQRGYNAQE'	713 - 730	0.0	0.8	2.0	1.1	0.9	15.9	8E-04	0.5	7E-02	9.1	1.0	8.0	1E-03	0.5	2E-01	9.1	1.0	8.0	1E-03	0.5	1E-01	9.1	1.0	8.0	*	
713	731	'MRVEDVDRLDQRGYNAQEY'	713 - 731	0.0	0.8	2.0	1.2	0.9	16.8	6E-04	0.5	6E-02	9.6	1.0	8.4	8E-04	0.5	1E-01	9.6	1.0	8.4	2E-14	0.5	1E-01	9.6	1.0	8.4	*	
714	721	'RVEDVDRL'	714 - 721	0.0	0.8	1.2	1.1	0.9	5.9	2E-14	0.5	7E-03	4.1	1.0	2.9	3E-14	0.5	5E-03	4.1	1.0	2.9	2E-14	0.5	4E-03	4.1	1.0	2.9		
714	730	'RVEDVDRLDQRGYNAQE'	714 - 730	0.0	0.8	1.9	1.1	0.9	14.9	5E-04	0.5	7E-02	8.5	1.0	7.5	8E-04	0.5	2E-01	8.5	1.0	7.5	1E-03	0.5	1E-01	8.5	1.0	7.5	*	
718	730	'VDRLDQRGYNAQE'	718 - 730	0.0	0.9	2.5	1.0	1.0	11.0	7E-04	0.5	9E-02	6.5	1.0	5.5	1E-03	0.6	3E-01	6.5	0.9	5.5	1E-03	0.6	2E-01	6.5	1.0	5.5		
718	731	'VDRLDQRGYNAQEY'	718 - 731	2.5	1.0	0.0	12.0	0.9	1.0	8E-02	1.0	3E-04	6.0	0.5	7.0	2E-01	1.0	9E-04	6.1	0.5	6.9	2E-01	1.0	1E-03	6.0	0.5	7.0	*	
722	730	'DQRGYNAQE'	722 - 730	0.0	0.9	3.3	1.0	1.0	7.0	2E-03	0.6	2E-01	4.2	1.0	3.8	2E-03	0.7	3E-01	3.4	0.6	4.6	2E-03	0.7	3E-01	3.4	0.6	4.6	*	
722	731	'DQRGYNAQEY'	722 - 731	0.0	0.9	3.2	1.0	1.0	8.0	1E-03	0.5	2E-01	4.6	1.0	4.4	8E-04	0.6	3E-01	4.1	0.5	4.9	1E-03	0.6	3E-01	4.3	0.5	4.7	*	*
731	738	'YYDRIPEL'	731 - 738	0.0	0.9	1.4	1.0	1.0	5.0	2E-14	0.5	6E-03	3.5	1.0	2.5	2E-14	0.5	4E-03	3.5	1.0	2.5	2E-14	0.5	3E-03	3.5	1.0	2.5		
731	740	'YYDRIPELRQ'	731 - 740	0.0	0.8	1.2	1.1	0.9	6.9	2E-14	0.5	9E-03	4.5	1.0	3.5	2E-14	0.5	8E-03	4.5	1.0	3.5	2E-14	0.5	6E-03	4.5	1.0	3.5		
732	738	'YDRIPEL'	732 - 738	0.0	0.8	1.4	1.0	1.0	4.0	2E-13	0.5	2E-01	3.0	1.0	2.0	2E-14	0.5	2E-01	3.0	1.0	2.0	2E-14	0.5	1E-01	3.0	1.0	2.0		
739	745	'RQIIEQL'	739 - 745	0.0	0.7	1.0	1.1	0.9	4.9	2E-07	0.5	4E-05	3.5	1.0	2.5	3E-08	1.0	2E-05	3.5	1.0	2.5	7E-13	1.0	5E-09	3.5	1.0	2.5		
739	748	'RQIIEQLSSG'	739 - 748	1.7	0.8	0.1	7.4	0.7	1.6	3E-04	1.0	4E-04	3.7	0.6	5.3	6E-04	1.0	8E-05	3.7	0.5	5.3	1E-03	1.0	3E-07	3.7	0.7	5.3		
745	757	'LSSGFFSPKQPDL'	745 - 757	0.0	0.8	2.8	1.0	1.0	9.0	1E-04	0.5	4E-02	5.5	1.0	4.5	1E-04	0.5	8E-02	5.5	1.0	4.5	9E-05	0.5	5E-02	5.5	1.0	4.5	*	*
746	757	'SSGFFSPKQPDL'	746 - 757	0.0	0.8	2.8	1.0	1.0	8.0	1E-04	0.5	5E-02	5.0	1.0	4.0	1E-04	0.5	8E-02	5.0	1.0	4.0	1E-04	0.5	7E-02	5.0	1.0	4.0		
746	758	'SSGFFSPKQPDLF'	746 - 758	0.0	0.7	2.2	1.0	1.0	9.0	8E-05	0.5	7E-02	5.5	1.0	4.5	2E-04	0.5	1E-01	5.5	1.0	4.5	5E-05	0.5	1E-01	5.5	1.0	4.5		
749	757	'FFSPKQPDL'	749 - 757	0.0	0.8	2.2	1.0	1.0	5.0	1E-03	0.6	3E-01	3.5	1.0	2.5	2E-04	0.6	3E-01	3.2	0.5	2.8	5E-04	0.6	3E-01	3.5	0.5	2.5		
750	757	'FSPKQPDL'	750 - 757	0.0	0.8	2.4	1.0	1.0	4.0	2E-03	0.6	6E-01	3.0	0.6	2.0	4E-04	0.5	4E-01	2.2	0.5	2.8	7E-04	0.6	4E-01	2.4	0.5	2.6		
751	757	'SPKQPDL'	751 - 757	0.0	0.9	1.9	1.0	1.0	3.0	1E-03	0.5	5E-01	2.5	0.9	1.5	1E-04	0.5	3E-01	2.0	0.5	2.0	3E-04	0.6	3E-01	2.3	0.5	1.7		
752	757	'PKQPDL'	752 - 757	0.0	0.9	1.9	1.0	1.0	3.0	1E-03	0.5	3E-01	1.5	1.0	1.5	3E-04	0.5	3E-01	1.3	0.5	1.7	5E-04	0.5	3E-01	1.4	0.5	1.6		
758	763	'FKDIVN'	758 - 763	0.0	0.6	0.7	1.0	1.0	4.0	1E-03	0.5	2E-02	3.0	1.0	2.0	9E-04	0.5	7E-03	3.0	1.0	2.0	1E-03	0.5	4E-03	3.0	1.0	2.0		
758	764	'FKDIVNM'	758 - 764	0.0	0.5	1.0	0.9	1.0	5.1	5E-05	0.5	5E-03	3.5	1.0	2.5	2E-14	0.5	4E-03	3.5	1.0	2.5	2E-04	0.5	2E-03	3.5	1.0	2.5		

				Intrinsic fits						GlyPa'						GlyPb'						GlyPb-G6P'						Significantly different peptides	
Start	End	Sequence	Residues	k_{int1}	β_1	k_{int2}	N_1	β_2	N_2	k_{exp1}	β_1	k_{exp2}	N_1	β_2	N_2	k_{exp1}	β_1	k_{exp2}	N_1	β_2	N_2	k_{exp1}	β_1	k_{exp2}	N_1	β_2	N_2	GlyPa vs GlyPb	GlyPb vs GlyP:G6P
759	764	'KDIVNM'	759 - 764	0.0	0.5	0.8	0.9	1.0	4.1	5E-04	0.5	1E-03	3.0	1.0	2.0	8E-04	0.6	6E-04	3.0	1.0	2.0	4E-04	0.8	2E-03	3.0	1.0	2.0	*	
765	771	'LMHHDRF'	765 - 771	0.0	0.5	5.6	1.0	1.0	5.0	4E-04	1.0	9E-04	3.5	1.0	2.5	3E-04	1.0	2E-04	3.5	1.0	2.5	3E-04	1.0	3E-04	3.5	1.0	2.5		
765	774	'LMHHDRFKVF'	765 - 774	0.0	0.6	3.0	1.0	1.0	8.0	3E-04	1.0	7E-05	5.0	1.0	4.0	6E-07	1.0	1E-04	5.0	1.0	4.0	5E-11	1.0	1E-05	5.0	1.0	4.0		
765	776	'LMHHDRFKVFAD'	765 - 776	2.5	1.0	0.3	10.1	0.6	0.9	7E-05	1.0	3E-06	5.1	0.6	5.9	2E-04	1.0	2E-09	5.1	0.9	5.9	2E-04	1.0	1E-05	5.1	1.0	5.9		
780	785	'YVKCQE'	780 - 785	0.0	0.5	2.5	1.0	1.0	4.0	1E-07	0.9	2E-05	3.0	1.0	2.0	3E-14	1.0	2E-09	3.0	1.0	2.0	3E-14	1.0	5E-09	3.0	1.0	2.0		
780	789	'YVKCQERVSA'	780 - 789	2.4	0.8	0.1	7.6	0.7	1.4	8E-05	1.0	2E-14	3.8	0.5	5.2	4E-05	1.0	2E-14	3.8	0.5	5.2	7E-05	1.0	4E-14	3.8	0.7	5.2		
780	790	'YVKCQERVSAL'	780 - 790	0.0	0.6	2.4	1.0	1.0	9.0	2E-14	0.7	5E-05	5.5	1.0	4.5	6E-07	0.8	3E-05	5.5	1.0	4.5	2E-07	0.9	2E-05	5.5	1.0	4.5		
790	796	'LYKNPRE'	790 - 796	0.0	0.7	2.0	1.0	1.0	4.0	7E-14	0.5	4E-01	3.0	1.0	2.0	3E-14	0.5	4E-01	3.0	1.0	2.0	3E-14	0.7	5E-01	3.0	1.0	2.0		
790	800	'LYKNPREWTRM'	790 - 800	0.0	0.7	1.9	1.0	1.0	8.0	3E-14	0.5	3E-03	5.0	1.0	4.0	2E-14	0.5	4E-03	5.0	1.0	4.0	3E-14	0.5	3E-03	5.0	1.0	4.0		
791	796	'YKNPRE'	791 - 796	0.0	0.8	3.0	1.0	1.0	3.0	1E-04	1.0	1E+00	2.5	1.0	1.5	4E-05	1.0	1E+00	2.5	1.0	1.5	4E-14	1.0	1E+00	2.5	1.0	1.5		
791	800	'YKNPREWTRM'	791 - 800	0.0	0.8	2.2	1.0	1.0	7.0	3E-14	0.5	3E-03	4.5	1.0	3.5	2E-14	0.5	3E-03	4.5	1.0	3.5	2E-14	0.5	3E-03	4.5	1.0	3.5		
801	811	'VIRNIATSGKF'	801 - 811	0.0	0.6	2.1	1.1	0.9	8.9	2E-06	0.5	4E-05	5.6	1.0	4.4	7E-03	1.0	2E-14	0.6	0.5	9.4	3E-14	0.8	7E-05	5.6	1.0	4.4		
801	814	'VIRNIATSGKFSSD'	801 - 814	1.1	0.8	4.2	6.5	0.7	6.5	4E-14	0.5	4E-04	9.8	1.0	3.2	5E-14	0.5	3E-04	9.8	1.0	3.2	2E-14	0.5	2E-04	9.7	1.0	3.2		
801	819	'VIRNIATSGKFSSDRTIAQ'	801 - 819	2.3	0.9	0.0	16.9	0.7	1.1	1E-04	1.0	4E-14	8.4	0.5	9.6	9E-05	1.0	2E-14	8.4	0.5	9.6	7E-05	1.0	2E-14	8.4	0.5	9.6		
801	820	'VIRNIATSGKFSSDRTIAQY'	801 - 820	0.0	0.7	2.4	1.4	0.8	17.6	3E-14	0.5	8E-05	10.2	1.0	8.8	4E-14	0.5	7E-05	10.2	1.0	8.8	2E-14	0.5	5E-05	10.2	1.0	8.8		
805	819	'IATSGKFSSDRTIAQ'	805 - 819	2.7	1.0	0.0	13.0	0.7	1.0	2E-04	1.0	2E-14	6.5	0.5	7.5	1E-04	1.0	6E-13	6.5	0.5	7.5	1E-04	1.0	2E-14	6.5	0.5	7.5		
807	814	'TSGKFSSD'	807 - 814	1.3	0.9	5.8	2.7	0.7	4.3	2E-14	0.5	3E-04	4.8	1.0	2.2	2E-14	0.5	3E-04	4.8	1.0	2.2	2E-14	0.5	2E-04	4.8	1.0	2.2		
808	814	'SGKFSSD'	808 - 814	1.2	0.9	5.1	2.3	0.7	3.7	8E-13	0.5	7E-04	4.2	1.0	1.8	2E-12	0.5	4E-04	4.2	1.0	1.8	2E-14	0.5	2E-04	4.2	1.0	1.8		
808	819	'SGKFSSDRTIAQ'	808 - 819	0.0	0.7	2.8	1.0	1.0	10.0	2E-14	0.5	5E-04	6.0	1.0	5.0	3E-14	0.5	4E-04	6.0	1.0	5.0	3E-14	0.5	4E-04	6.0	1.0	5.0		
812	819	'SSDRTIAQ'	812 - 819	0.0	0.7	2.4	1.0	1.0	6.0	2E-14	0.5	3E-04	4.0	1.0	3.0	4E-14	0.5	2E-04	4.0	1.0	3.0	2E-11	0.5	9E-05	4.0	1.0	3.0		
820	825	'YAREIW'	820 - 825	0.0	0.6	1.6	1.0	1.0	4.0	3E-14	0.5	3E-04	3.0	1.0	2.0	3E-14	0.5	2E-04	3.0	1.0	2.0	2E-14	0.5	1E-04	3.0	1.0	2.0		
820	828	'YAREIWGVE'	820 - 828	0.0	0.7	1.1	1.0	1.0	7.0	2E-13	0.5	2E-03	4.5	1.0	3.5	4E-14	0.5	2E-03	4.5	1.0	3.5	7E-14	0.5	2E-03	4.5	1.0	3.5		
820	842	'YAREIWGVEPSRQRLPAPDEKIP'	820 - 842	2.4	0.8	0.8	10.1	0.9	7.9	1E-01	0.5	7E-10	11.2	0.5	6.8	5E-01	0.5	1E-04	10.0	0.6	8.0	5E-01	0.5	1E-04	10.2	0.7	7.8	*	
821	842	'AREIWGVEPSRQRLPAPDEKIP'	821 - 842	2.4	0.8	0.8	9.2	0.9	7.8	1E-01	0.5	2E-07	11.9	0.5	5.1	5E-01	0.5	3E-04	10.8	0.6	6.2	5E-01	0.5	3E-04	10.9	0.7	6.1	*	
826	842	'GVEPSRQRLPAPDEKIP'	826 - 842	3.2	0.8	1.2	3.9	1.0	8.1	1E+00	0.6	7E-02	2.0	0.8	9.8	1E+00	0.7	8E-02	6.4	0.9	5.5	1E+00	0.7	8E-02	6.4	1.0	5.5	*	
829	842	'PSRQRLPAPDEKIP'	829 - 842	1.2	1.0	3.4	6.1	0.8	3.9	1E-02	0.6	2E-01	3.1	0.5	5.9	5E-02	0.8	1E+00	3.3	0.5	5.6	6E-02	0.9	1E+00	3.3	0.7	5.7	*	

(1) Bai, Y.; Milne, J. S.; Mayne, L.; Englander, S. W. *Proteins* **1994**, *20* (1), 4-14.
(2) Bai, Y.; Milne, J. S.; Mayne, L.; Englander, S. W. *Proteins* **1993**, *17* (1), 75-86.
(3) Hageman, T. S.; Weis, D. D. *Anal Chem* **2019**, *91* (13), 8008-8016.
